# Synthesis of Optically Active *syn*- and *anti*-Chlorohydrins through a Bienzymatic Reductive Cascade

**DOI:** 10.1021/acs.orglett.2c02592

**Published:** 2022-09-26

**Authors:** Jorge González-Rodríguez, Jesús Albarrán-Velo, Raquel G. Soengas, Iván Lavandera, Vicente Gotor-Fernández, Humberto Rodríguez-Solla

**Affiliations:** Organic and Inorganic Chemistry Department, University of Oviedo, Avenida Julián Clavería s/n, 33006 Oviedo, Spain

## Abstract

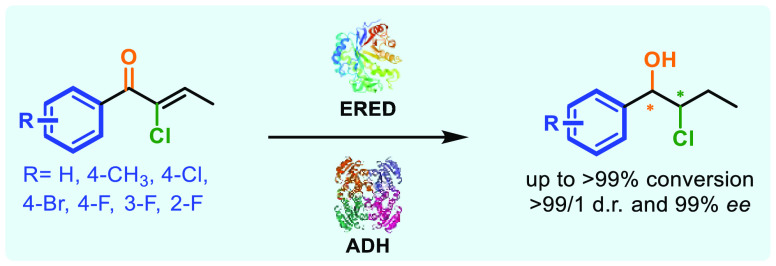

A bienzymatic cascade has been designed and optimized to obtain enantiopure chlorohydrins starting from the corresponding 1-aryl-2-chlorobut-2-en-1-ones. For the synthesis of these α-chloroenones, a two-step sequence was developed consisting of the allylation of the corresponding aldehyde with 3-dichloroprop-1-ene, followed by oxidation and further isomerization. The selective cooperative catalytic system involving ene-reductases (EREDs) and alcohol dehydrogenases (ADHs) afforded the desired optically active chlorohydrins under mild reaction conditions in excellent conversions (up to >99%) and selectivities (up to >99:1 diastereomeric ratio (dr), >99% enantiomeric excess (*ee*)).

Optically active chlorohydrins are valuable intermediates in the synthesis of diverse families of organic compounds,^1^ such as amino alcohols,^1a,1b^ epoxides,^1c−1e^ glycols,^1f^ pyrrolidines,^1g^ cyclopropanes,^1h^ hydroxy nitriles,^1i,1j^ and azides.^1k,1l^ Traditional synthetic methods toward enantiopure chlorohydrins are based on the action of metals, including transition metal catalyzed asymmetric transfer hydrogenation,^2^ Meerwein–Ponndorf–Verley reduction^3^ or hydroboration^4^ of α-chloroketones, and enantioselective addition of chlorinated nucleophiles to carbonyl compounds.^5^ The use of expensive and/or toxic metallic reagents, strict reaction conditions, and volatile organic solvents can hamper the requirements for sustainable chemical processes, so the use of enzymes opens new possibilities in this field. In this regard, several enzymatic methods have been reported to provide access to chlorohydrins with high enantiomeric excess, including biocatalytic hydrogen-transfer reduction of prochiral α-chloroketones^6^ and kinetic resolution^7^ or deracemization^8^ of racemic chlorohydrins.

Nowadays, multienzymatic synthesis and the development of robust cascades is attracting great attention due to the ability of enzymes to work under mild reaction conditions and in an orchestral manner to achieve chemo-, regio-, and stereoselective transformations.^9^ Hence, in the search for an alternative enzymatic synthesis of halohydrins capable of overcoming the inherent limitations of kinetic resolution-based procedures, Gatti and co-workers described the stereoselective synthesis of chiral bromohydrins via a bienzymatic ene-reductase (ERED)–alcohol dehydrogenase (ADH) cascade.^10^ More recently, the same authors reported a methodology based on an ERED–ADH cascade for the preparation of halohydrins from tetrasubstituted cyclic enones.^11^ The aforementioned protocols were applied to both cyclic and aliphatic α-bromo- and α-chloroenones, and the design of sequential cascades is highly dependent on the cross-reactivity and substrate specificity displayed by these families of reductive enzymes. Herein, a straightforward methodology is described for the preparation of enantiopure aromatic chlorohydrins from chloroenones based on a bienzymatic reductive cascade involving the ERED-catalyzed reduction of the C–C double bond followed by the ADH-catalyzed reduction of the carbonyl group (Scheme 1).

**Scheme 1:**
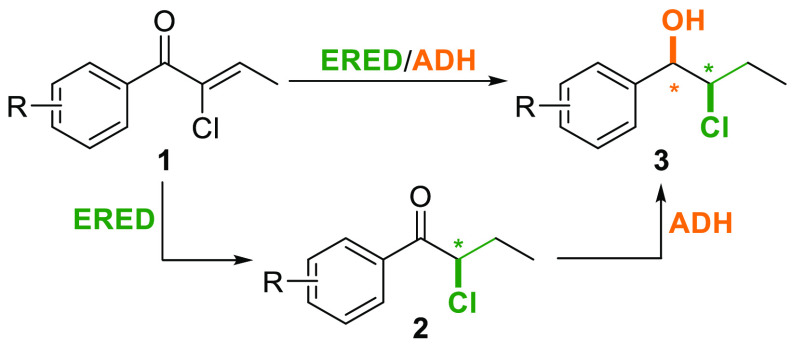
Proposed Bienzymatic Reduction Cascade of 1-Aryl-2-chlorobutan-1-ols

Our proposed enzymatic cascade is based on the use of 1-aryl-2-chlorobut-2-en-1-ones **1** as starting materials. Accordingly, a short and reliable procedure for the synthesis of these derivatives in high yields was needed. Classical synthetic methods for the preparation of α-chloroenones are based on the chlorination of ketimines in carbon tetrachloride^12^ or the intermediacy of organomercury^13^ or organoselenium^14^ derivatives. A procedure involving the reaction of chloroallyllithium with esters, which avoids the use of highly toxic reagents and solvents, was described some years ago.^15^ However, this method gave moderate results in terms of yield and stereoselection. More recently, Sadhukhan and Baire described the halo-Meyer–Schuster rearrangement of propargyl acetates to (*Z*)-haloenones.^16^ Despite the excellent yields, the main drawback is the preparation of the starting propargyl compounds involving a multistep sequence.

We envisioned the preparation of chloroenones by the indium-promoted allylation of aldehydes, followed by oxidation of the resulting racemic chlorohydrins and subsequent isomerization of the double bond to the more stable α,β-unsaturated position, as depicted in Scheme 2. Thus, Barbier allylation of a series of benzaldehydes **4a**–**g** with 1,3-dichloroprop-1-ene (**5**) in the presence of indium powder (1.0 equiv) and sodium iodide (2.0 equiv) afforded the corresponding chlorohydrins **6a**–**g** in good yields (68–88%) and moderate *syn* selectivities (79:21 to 81:19).^17^ After oxidation with Dess–Martin periodinane (DMP), isomerization of the resulting β,γ-unsaturated chloroketones aided by silica gel exclusively afforded the more stable α,β-unsaturated chloroketones **1a**–**g** in good yields (91–98%).

**Scheme 2:**
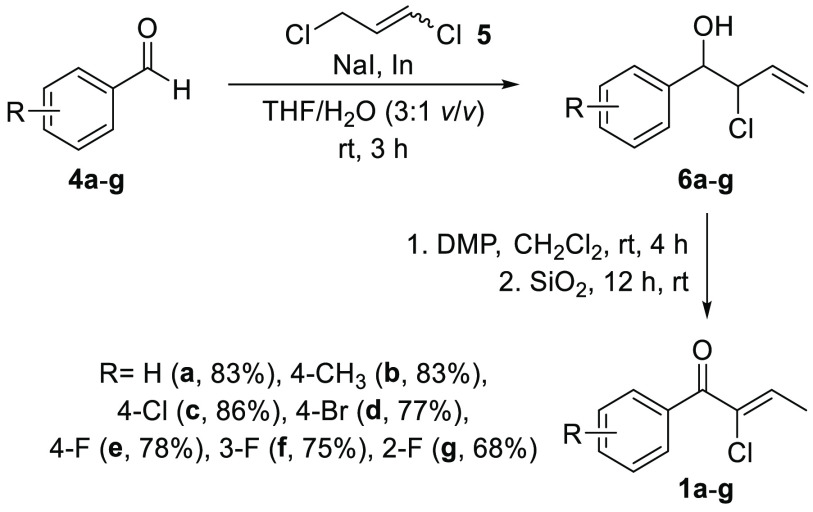
Synthesis of α-Chloroenones **1a**–**g**

Once a reliable procedure for the preparation of the chloroenones was developed, the next step was to explore the viability of the ERED–ADH catalytic system to perform the stereoselective reduction of both the alkene and the ketone moieties. To begin our studies, (*Z*)-2-chloro-1-phenylbut-2-en-1-one (**1a**, 4.3 mg) was selected as the model substrate. First, a screening of commercial EREDs was performed under standard conditions (25 mM of substrate, 30 °C, 24 h, and 250 rpm) with the glucose and glucose dehydrogenase (GDH) system to recycle the nicotinamide cofactor (NADP) using two different buffers: (i) a citrate buffer (pH 5) in which the transformation proceeded sluggishly (see Table S1) and (ii) a phosphate buffer (KPi, pH 7) that led to the highest conversions for all the seven tested EREDs (Table 1). Five EREDs led to (*R*)-α-chloroketone **2a**, up to a remarkable 94% enantiomeric excess (*ee*) and quantitative conversion with ERED–110 (entry 2), while (*S*)-**2a** was obtained in 78% conversion and 85% *ee* using ERED–P1-H09 (entry 7). The absolute configurations of **2a** were assigned by comparison with the examples already described in the literature.^18^

**Table 1:**
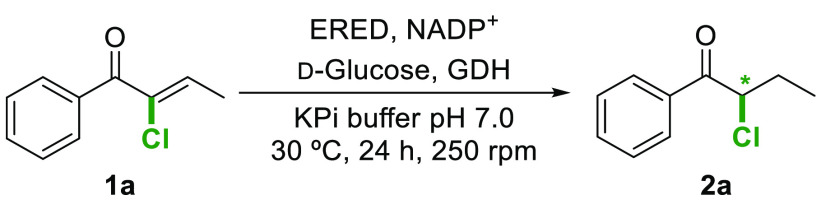
ERED-Catalyzed Bioreduction of Chloroenone **1a**^a^

entry	ERED	*c* (%)^b^	*ee* (%)^c^
1	103	89	88 (*R*)
2	110	99	94 (*R*)
3	112	7	n.d.
4	207	88	92 (*R*)
5	P1-A04	>99	78 (*R*)
6	P1-E01	72	77 (*R*)
7	P1-H09	78	85 (*S*)

a For further details, see Table S1.

b Determined by GC.

c Determined by HPLC. Major enantiomer in parentheses. n.d.: not determined.

ERED–110 was then selected for optimization of the reaction conditions, by analyzing different parameters that could affect the individual action of this catalyst but also its compatibility with ADHs. Thus, the influence of the enzyme loading, substrate concentration, organic cosolvent, temperature, and reaction time was studied (see Tables S2–S8). Hence, it was observed that just 3.0 mg of the lyophilized powder containing ERED–110 (70 wt % regarding substrate **1a**) afforded **2a** with 73% conversion and 96% *ee* after 4 h, so these conditions were selected for the further optimization. Interestingly, the use of short reaction times was beneficial since slow racemization of ketone **2a** was observed within the time.

This fact was demonstrated by incubating enantiopure (*R*)-**2a** under standard reaction conditions for 24 h, lowering the optical purity to 86% *ee*. Increasing the temperature was attempted in order to rise the conversion with low loadings of ERED–110, but conversions remained constant, while the enantiomeric excess slightly decreased (Table S3). A key parameter in enzymatic redox catalysis is the presence of organic cosolvents because their use in low amounts can benefit the development of biotransformations at higher substrate concentrations without inactivating the enzyme. For that reason, a series of solvent systems were attempted (5% vol, Figure 1 and Table S4).

**Figure 1:**
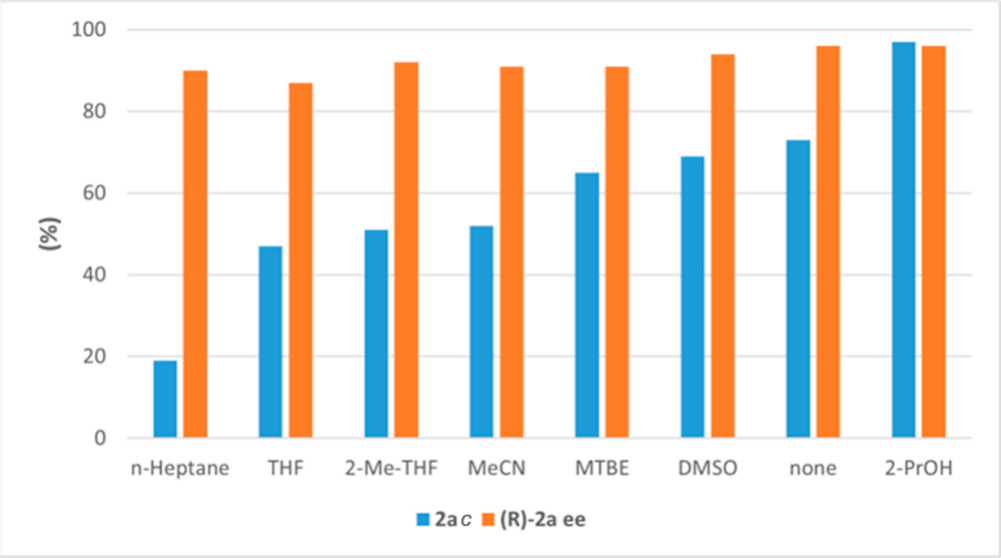
Role of the cosolvent (5% vol) in the ERED–110-catalyzed bioreduction of **1a** (25 mM, KPi buffer, pH 7, 30 °C, 4 h, 250 rpm).

Only 2-propanol (2-PrOH) improved the results in terms of conversion (97%), while maintaining the selectivity (96% *ee*). Remarkably, the use of alcohols such as 2-PrOH can also help for cofactor recycling purposes with ADHs, providing an additional advantage to the overall catalytic system. Gladly, under these conditions, we observed similar results (98% conversion, 96% *ee*) with just 2.0 mg of ERED–110 (46 wt % regarding substrate **1a**, entry 9, Table S4). A range of 2-PrOH percentages (2.5–10% vol, entries 1–4, Table 2) was attempted, and 5% was the optimal (entry 2). Decreasing the temperature from 30 to 25 °C improved both the conversion (97% to >99%) and the enantioselectivity (93% to 97% *ee*) (entry 5). Interestingly, 3 h was found to be the shortest time to reach full conversion (entry 6). Finally, the bioreductions were carried out at different substrate concentrations (25–100 mM), but the initial 25 mM resulted to be superior (Table S7). The best reaction conditions found for the ERED–110 enzyme to produce chloroketone (*R*)-**2a** (Table 2, entry 6) were next applied to the (*S*)-selective ERED–P1-H09 to achieve its antipode (*S*)-**2a**, which was recovered in 99% *ee* but in a modest 28% conversion (Table S8). This result could be improved using 4.0–6.0 mg of the enzyme (46–50%), maintaining the selectivity.

**Table 2:** Optimization of ERED–110-Catalyzed Bioreduction of Chloroenone **1a** to Obtain (*R*)-**2a**

entry^a^	2-PrOH (% vol)	*T* (°C)	*t* (h)	*c* (%)^b^	*ee* (%)^c^
1	2.5	30	4	90	91
2	5.0	30	4	97	93
3	7.5	30	4	83	94
4	10.0	30	4	86	94
5	5.0	25	4	>99	97
6	5.0	25	3	>99	98
7	5.0	25	2	94	95

a For further details, see Tables S5 and S6.

b Determined by GC.

c Determined by HPLC.

The identification of suitable ADHs for carbonyl reduction would provide access to the different clorohydrin **3a**–**g** diastereoisomers, in particular, since they were not able to directly reduce enone **1a** to the corresponding allylic alcohol (*Lb*ADH and *TeS*ADH) or if the (reversible) ADH-catalyzed reduction was significantly slower than the (quasi-irreversible) ERED-catalyzed one (evo.1.1.200, Table S9). In this sense, we envisaged that the thermodynamically favored bioreduction of α-chloroketones **2**(19) in contrast to the disfavored reduction of unsaturated carbonyl compounds could successfully drive this cascade. The selected combinations of ERED and ADH that show the best results in terms of activity and stereoselectivity to obtain **3a** are displayed in Table 3 (for further information on the ADH screening and optimization of the cascade, see Tables S10 and S11, respectively). The use of both in house ADHs heterologously expressed in *E. coli* and commercial ones allowed the synthesis of up to three out of the four possible enantiomers with excellent conversions and selectivities (*ee* and diastereomeric ratio (dr)).

**Table 3:**
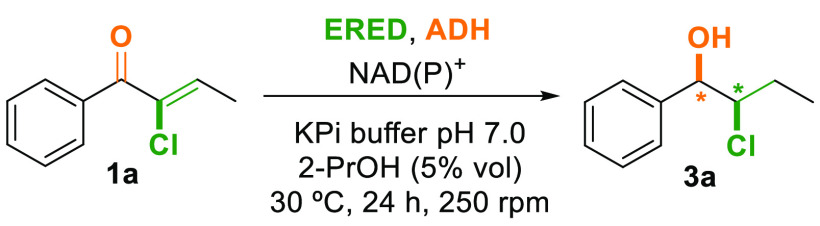
Screening of ADHs Combined with the Selected EREDs for the One-Pot Cascade Bioreduction of **1a**^a^

entry	ERED	ADH	*c* (%)^b^	*syn/anti*^c^	*syn*-**3a** *ee* (%)^c^	*anti*-**3a** *ee* (%)^c^
1	110	*Lb*ADH	>99	99:1	98 (1*R*,2*R*)	n.d.
2	110	*TeS*ADH	98	96:4	98 (1*R*,2*R*)	n.d.
3	110	ADH-T	>99	>99:1	96 (1*R*,2*R*)	n.d.
4	110	ADH-A	>99	98:2	93 (1*R*,2*R*)	n.d.
5	110	evo.1.1.200	>99	<1:>99	n.d.	>99 (1*S*,2*R*)
6	110	KRED-P2-D03	>99	<1:>99	n.d.	99 (1*S*,2*R*)
7	110	KRED-P3-B03	>99	>99:<1	91 (1*S*,2*S*)	n.d.
8	P1-H09	*Lb*ADH	>99	>99:<1	85 (1*S*,2*S*)	n.d.
9	P1-H09	*TeS*ADH	90	>99:<1	92 (1*S*,2*S*)	n.d.

a For further details, see Table S9.

b Determined by GC.

c Determined by HPLC. Major diastereoisomer in parentheses. n.d.: not determined.

For instance, the use of ERED–110 with ADHs from *Lactobacillus brevis* (*Lb*ADH), *Thermoanaerobacter ethanolicus* (*TeS*ADH), *Thermoanaerobacter* species (ADH-T), and *Rhodococcus ruber* (ADH-A) gave access to (1*R*,2*R*)-**3a** with excellent conversions and optical purities (entries 1–4). The same ERED in combination with commercial ADHs with opposite selectivity to the previous enzymes led to (1*S*,2*R*)-**3a** (entries 5 and 6), while (1*S*,2*S*)-**3a** was also obtained with excellent results when the ERED was changed (entries 8 and 9). The viability of the one-pot cascade depends on how the ADH accepts the ketone enantiomer obtained from the ERED-catalyzed reduction. For example, the selectivity achieved toward the model substrate **1a** was the one predicted for ERED–110 and evo.1.1.200 (1*S*,2*R*), as ERED yields the (*R*)-isomer and the ADH, which in this case shows anti-prelog selectivity, affords the (*S*)-isomer. However, the system proved to be complex since it was observed that in some cases a flipped binding of the ketone intermediate **2a** was possible in the active site of the ADH, thus providing an unexpected isomer as in the case of the pair ERED–110 and *Lb*ADH, also an anti-prelog ADH, since the (1*R*,2*R*)-isomer was found to be a unique diastereoisomer. This effect is due to the large size of both substituents of ketone **2a**. In addition, a dynamic process was also observed in some cases, in particular when the enantiomer of the intermediate ketone was not an optimal substrate for the ADH, as for instance (*R*)-**2a** in the case of KRED–P3-B03 (entry 7), giving access to (1*S*,2*S*)-**3a** due to the racemization of (*R*)-**2a**, completely shifting the equilibrium toward the final product.

The treatment of enantiopure **3a**, isolated from the reaction with ERED–110 and evo.1.1.200 (Table 3, entry 5), with 3.0 equiv of KOH led to the corresponding epoxide, and the measurement of the coupling constants in the ^1^H NMR (300 MHz) of the reaction crude allowed one to unambiguously determine the stereochemistry of both chiral centers of the epoxide and, hence, of compound (1*S*,2*R*)-**3a** (see the Supporting Information for further details).

The scope of this bienzymatic methodology was then explored considering aromatic substrates incorporating electron withdrawing groups (F, Cl, Br) at different positions in the aromatic ring and the 4-methyl group as an example of an electron donor moiety (Scheme 3). The proper combination of ERED–110 or ERED–P1-H09 with the ADH from *Ralstonia* sp. (*Ras*ADH)^20^ or commercial evo.1.1.200 afforded several halohydrin stereoisomers in high conversions and selectivities. Overall, multiple synthetically useful possibilities with conversions over 85% and from good to excellent stereoselectivities were achieved (for detailed information, see Tables S10–S15). Finally, the scalability of the biocatalytic cascade was demonstrated in semipreparative scale for the reduction of ketone **1a** (50 mg) to afford enantiomerically pure chlorohydrins (1*S*,2*R*)-**3a**, (1*R*,2*R*)-**3a**, and (1*S*,2*S*)-**3a** (87–91% isolated yield, Table S16) and also at 1 mmol (160 mg) scale for **1a** to obtain (1*S*,2*R*)-**3a** (81% isolated yield).

**Scheme 3:**
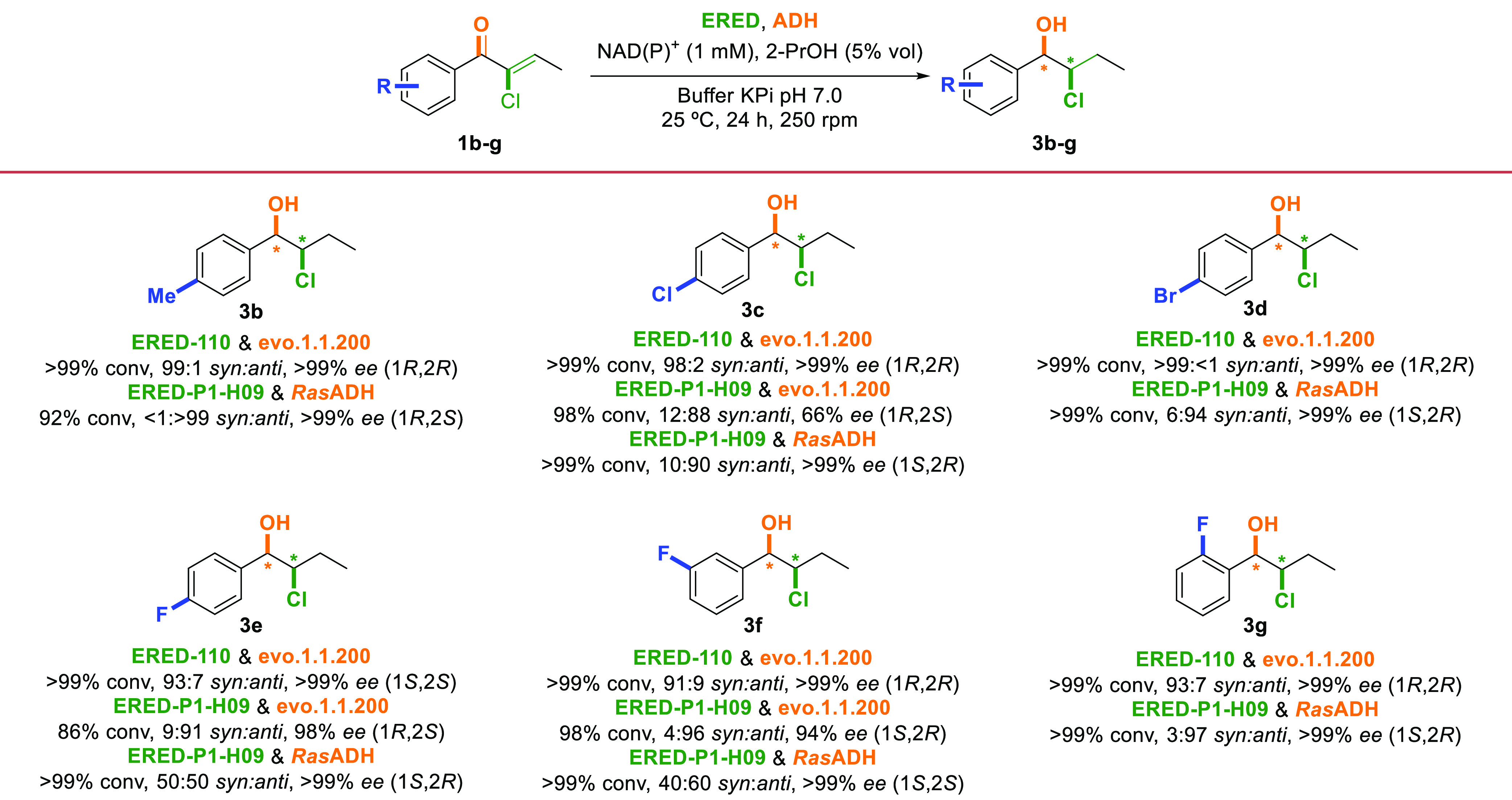
Scope of the Bienzymatic ERED–ADH System towards the Synthesis of Optically Active Chlorohydrins **3b**–**g**

In conclusion, a general methodology has been described for the synthesis of a series of 1-aryl-2-chlorobut-2-en-1-ones, which were later doubly reduced using an ERED–ADH system through a stereodivergent cascade. Optically active aromatic chlorohydrins have been prepared in a selective manner under mild reaction conditions using 2-PrOH as the cofactor recycling system for both steps. Depending on the ERED and ADH of choice, in most cases, up to three out of the four possible enantiomers of chlorohydrins **3a**–**g** were separately obtained with excellent conversions and good selectivities.

## Supporting Information Available

Chromatograms for chiral analyses; ^1^H NMR, ^13^C NMR, and ^19^F NMR spectra; extensive enzyme-catalyzed transformation screenings; optimization of the reaction conditions (PDF)
