# Supplementary material for: Synthesis of Optically Active syn- and anti-Chlorohydrins through a Bienzymatic Reductive Cascade
Source: Org Lett. 2022 Sep 26;24(39):7082–7. doi: 10.1021/acs.orglett.2c02592 (PMC9552227; doi:10.1021/acs.orglett.2c02592)
Supplement: Supplementary file 1 — ol2c02592_si_001.pdf [file ol2c02592_si_001.pdf]

Synthesis of Optically Active *syn*- and *anti*-Chlorohydrins through a Bienzymatic Reductive Cascade

Jorge González-Rodríguez, Jesús Albarrán-Velo, Raquel G. Soengas, Iván Lavandera, Vicente Gotor-Fernández* and Humberto Rodríguez-Solla*

Organic and Inorganic Chemistry Department. University of Oviedo.

Avenida Julián Clavería 8, 33006 Oviedo, Spain.

E-mail: hrsolla@uniovi.es (H.R.-S.); vicgotfer@uniovi.es (V.G.-F.).

Index (Page 1 out of 102)

I. General considerations S3

II. Synthesis of starting chloroenones **1a-g** S4

II.1. Chloroallylation of aldehydes S4

II.2. Oxidation-isomerization of chlorohydrins S5

III. Synthesis of analytical standards S7

*III.1. Synthesis of racemic chloroketones **2a-g*** S7

*III.2. Synthesis of racemic chlorohydrins **3a-g*** S10

IV. Bioreduction of chloroenone **1a** to chloroketone **2a** S11

IV.1. Screening of EREDs S11

IV.2. Optimization of the ERED-catalyzed process S12

IV.3. Carbonyl bioreduction studies S14

V. Cascade bioreduction of chloroenones **1a-g** to chlorohydrins **3a-g** S16

V.1. Screening of EREDs and ADHs S16

V.2. General experimental procedures S19

*V.3. Assignment of the absolute configuration of chlorohydrin **1a*** S21

*V.4. Scope of the cascade bienzymatic reduction of chloroenones **1b-g*** S22

*V.5. Scale-up of the bienzymatic synthesis of chlorohydrin **3a*** S29

VI. Reference section S30
VII. Analytical data S32
VII.1. GC for the determination of the conversions S32
VII.2. HPLC analyses for the determination of enantiomeric excess S33
VII.3. NMR spectra S58

I. General considerations

Chemical reagents and nicotinamide cofactors ($NADP^+$ and NAD^+) were purchased from Sigma-Aldrich. All starting products, namely **1a**-**g**, and standards **2a**-**g** and **3a**-**g** were chemically synthesized (see below synthetic procedures and full characterization). Cascade bienzymatic reactions were performed in 2 mL-Eppendorf tubes. In house ADHs were heterologously expressed in *E. coli*, plated, grown, and lyophilized prior their use as described previously: *Ralstonia* species (*Ras*ADH),[1] *Sphingobium yanoikuyae* (*Sy*ADH),[2] *Thermoanaerobacter* species (ADH-T),[3] *Lactobacillus brevis* (*Lb*ADH),[4] *Thermoanaerobacter ethanolicus* (*TeS*ADH),[5] *Rhodococcus ruber* (ADH-A)[6] and horse liver (HLADH).[7] These enzymatic preparations showed an activity of approximately 0.5–1 U/mg. EREDs and KREDs were received from Codexis Inc. Both commercial and in house enzymes are non-purified lyophilized enzymatic preparations that were lyophilized prior their use.

Gas chromatography (GC) analyses were performed on a Hewlett-Packard 6860 chromatograph equipped with a FID detector using the HP-1 column (30 m x 0.32 mm x 0.25 μm) for the determination of product percentages and conversion values. High performance liquid chromatography (HPLC) analyses were performed on an HPLC chromatograph Agilent 1100 Series equipped with a VIS-UV detector using a Chiralcel OD-H, Chiralcel OJ-H, Chiralpak AD-H and Chiralpak IC columns (25 cm x 4.6 mm, 5 μm particle size) for the measurement of enantiomeric excess value.

NMR spectra were recorded on a Bruker 300 MHz spectrometer including ^{1}H, ^{13}C, and ^{19}F NMR experiments. All chemical shifts (δ) are given in parts per million (ppm) and referenced to the residual solvent signal as internal standard. Melting points were measured in a Gallenkamp apparatus introducing the samples in open capillary tubes and the measurements are uncorrected. IR spectra were recorded in neat form on a Jasco FT/IR-4700 spectrophotometer and, and ν_{max} values are given in cm^{-1} for the main absorption bands. High resolution mass spectra (HRMS) experiments were carried out by electrospray ionization in positive mode (ESI^+) and APCI-DIP-TOF using an ESI-qTOF Bruker Impact II spectrometer and an API400 spectrometer from applied, respectively. Thin-layer chromatography (TLC) was conducted with Silica Gel 60 F254 precoated plates and visualized with a UV lamp, plus either potassium permanganate or

vanillin stains. Column chromatography was performed using silica gel 60 (230-240 mesh).

II. Synthesis of starting chloroenones 1a-g

II.1. Chloroallylation of aldehydes

The procedure for the synthesis of racemic chlorohydrins is adapted from those described in the literature (Scheme S1).[8]

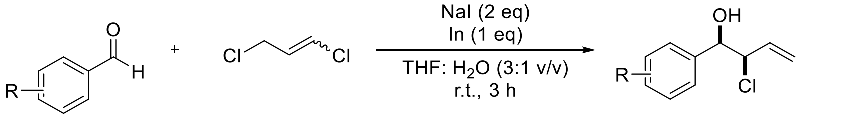

4a-g (1 eq) **5** (2 eq) *rac-syn*-**6a-g** (71-85%)

Scheme S1. Synthesis of allylic β-chlorohydrins **6a-g**.

To a solution of the corresponding benzaldehyde **4a-g** (10 mmol, 1 equiv) in a mixture of THF:H_2O (3:1 v/v) (24 mL), 1,3-dichloroprop-1-ene (**5**, 1.85 g, 20 mmol, 2 equiv), sodium iodide (NaI, 3.0 g, 20 mmol, 2 equiv) and indium powder (1.15 g, 10 mmol, 1 equiv) were added. The mixture was stirred at r.t. for 3 h and then diluted with water (15 mL) and extracted with Et_2O (3 × 20 mL). The combined organic layers were washed with a saturated aqueous $Na_2S_2O_3$ solution (1 x 25 mL), water (1 x 25 mL) and brine (1 x 25 mL), dried over Na_2SO_4, filtered, and evaporated under reduced pressure. Purification by column chromatography (Hex:EtOAc 10:1) afforded the corresponding chlorohydrins **6a-g** in moderate to good yields**:** **6a** (1.55 g, 85% yield), **6b** (1.69 g, 86% yield), **6c** (1.91 g, 88% yield), **6d** (2.22 g, 85% yield), **6e** (1.69 g, 84% yield), **6f** (1.58 g, 79% yield), and **6g** (1.42 g, 71% yield), and moderate d.r. (*syn*:*anti* 80:20 for **6a**, **6c** and **6f**, 79:21 for **6b** and **6g**, and 81:19 for **6d** and **6e**). The spectroscopic data for known compounds **6a-e** were consistent with those reported in literature.[8-10] Analytical data of novel compounds **6f** and **6g** are given below.

2-Chloro-1-(3-fluorophenyl)but-3-en-1-ol (**6f**): Pale yellow oil (1.58 g, 79% yield). R_f (Hex:EtOAc 10:1): 0.34. IR: ν 3433, 1933, 1904, 1795, 1651, 1493 and 799 cm^{-1}. ^{1}H-NMR (300.13 MHz, $CDCl_3$): δ 7.29 (dt, *J* = 14.3, 7.3 Hz, 1H), 7.11 (t, *J* = 8.5 Hz, 2H), 7.01 (ddd, *J* = 17.0, 10.2, 8.2 Hz, 1H), 5.82 (ddd, *J* = 17.0, 10.2, 8.2 Hz, 1H), 5.41–5.06 (m, 2H), 4.73 (d, *J* = 6.4 Hz, 1H), 4.53 (q, *J* = 7.8, 7.0 Hz, 1H), 2.64 (s, 1H). ^{13}C-NMR

(75.5 MHz, $CDCl_3$): δ 162.8 (d, *J* = 246.4 Hz, C), 141.7 (*d*, *J* = 7.4 Hz, C), 134.0 (CH), 129.9 (*d*, *J* = 8.2 Hz, CH), 122.6 (d, *J* = 2.7 Hz, CH), 119.8 (CH_2), 115.4 (d, *J* = 21.2 Hz, CH), 114.0 (d, *J* = 22.3 Hz, CH), 76.7 (C), 69.0 (C). ^{19}F-RMN (282 MHz, $CDCl_3$): δ –112.59 (major isomer), –112.68 (minor isomer).

2-Chloro-1-(2-fluorophenyl)but-3-en-1-ol (**6g**): Pale yellow oil (1.42 g, 71% yield). R_f (Hex:EtOAc 10:1): 0.33. IR: ν 3427, 1921, 1824, 1805, 1794, 1751, 1632, 1559, 1490 and 791 cm^{-1}. ^{1}H-NMR (300.13 MHz, $CDCl_3$): δ 7.60–7.39 (m, 1H), 7.33–7.22 (m, 1H), 7.17 (t, *J* = 7.3 Hz, 1H), 7.06–6.92 (m, 1H), 5.93 (dt, *J* = 17.2, 9.3, 1H), 5.35–4.94 (m, 4H), 4.61 (t, *J* = 7.6 Hz, 1H), 2.66 (s, 1H). ^{13}C-NMR (75.5 MHz, $CDCl_3$): δ 160.2 (d, *J* = 246.1 Hz, C) 134.5 (CH), 130.3 (d, *J* = 8.4 Hz, CH), 128.5 (d, *J* = 3.7 Hz, CH), 127.1, (d, *J* = 13.0 Hz, C), 124.7 (d, *J* = 3.1 Hz, CH), 120.0 (CH_2), 115.7 (d, *J* = 21.9 Hz, CH), 71.5 (CH), 69.2 (CH). ^{19}F-RMN (282 MHz, $CDCl_3$): δ –112.59 (major isomer), –118.27 (minor isomer).

II.2. Oxidation-isomerization of chlorohydrins

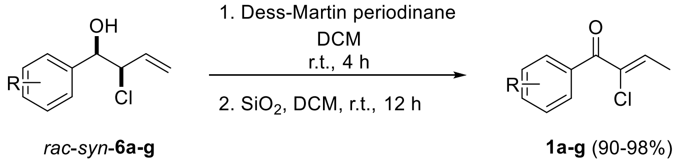

Scheme S2. Synthesis of α-chloroenones **1a-g**.

Dess-Martin periodinane (3.18 g, 7.5 mmol, 1.5 equiv) was added to a solution of the corresponding allylic β-chlorohydrin **6a-g** (5 mmol, 1 equiv) in dichloromethane (DCM, 12 mL). After stirring for 4 h at r.t., the mixture was diluted with more DCM (10 mL), quenched with a saturated aqueous $NaHCO_3$/$Na_2S_2O_3$ solution (50% *v*/*v*) and left stirring for 20 minutes. The resulting aqueous layer was extracted with DCM (3 × 20 mL). The combined organic layers were successively washed with a saturated aqueous $NaHCO_3$ solution (2 x 25 mL), a saturated aqueous $Na_2S_2O_3$ solution (1 x 25 mL), distilled water (1 x 25 mL) and brine (1 x 25 mL), dried over Na_2SO_4, filtered, and the solvent evaporated under reduced pressure.

To a solution of the obtained crude residue in DCM, SiO_2 was added (1:1 w/w substrate:SiO_2) and the mixture was stirred at r.t. for 12 h. Purification by column chromatography (Hex:EtOAc 20:1) afforded the corresponding chloroenones **1a-g** in good yields: **1a** (0.89 g, 98% yield), **1b** (0.94 g, 97% yield), **1c** (1.05 g, 98% yield), **1d**

(1.18 g, 91% yield), **1e** (0.92 g, 93% yield), **1f** (0.94 g, 95% yield), and **1g** (0.95 g, 96% yield). The spectroscopic data of the compound **1a** matched with the reported in the bibliography.[11] Characterization data of novel compounds **1b-g** can be found below.

(***Z***)**-2-Chloro-1-(*4*-methyl)but-2-en-1-one** (**1b**): Pale yellow oil (0.94 g, 97% yield). R_f (Hex:EtOAc 20:1): 0.54. IR: ν 3207, 2923, 1454, 1192, 1049, 769, 750 cm^{-1}. ^{1}H-NMR (300.13 MHz, $CDCl_3$): δ 7.61 (d, *J* = 8.0 Hz, 2H), 7.25 (d, *J* = 8.1 Hz, 2H), 6.73 (q, *J* = 6.8 Hz, 1H), 2.42 (s, 3H), 2.04 (d, *J* = 6.8 Hz, 3H). ^{13}C-NMR (75.5 MHz, $CDCl_3$): δ 190.0 (C), 143.3 (C), 139.5 (CH), 134.4 (C), 134.1 (C), 129.6 (2 x CH), 129.0 (2 x CH), 21.6 (CH_3), 15.4 (CH_3). HRMS (ESI$^+$, m/z): calcd. for $C_{11}H_{12}ClO$ ([M + H]$^+$) 195.0571; found, 195.0577.

(***Z***)**-2-Chloro-1-(4-chlorophenyl)but-2-en-1-one** (**1c**): White solid (1.05 g, 98% yield). R_f (Hex:EtOAc 20:1): 0.51, Mp: 55-56 ºC. IR: ν 1654, 1586, 1197, 828, 740 cm^{-1}. ^{1}H-NMR (300.13 MHz, $CDCl_3$): δ 7.65 (d, *J* = 8.4 Hz, 2H), 7.43 (d, *J* = 8.4 Hz, 2H), 6.74 (q, *J* = 6.8 Hz, 1H), 2.05 (d, *J* = 6.8 Hz, 3H). ^{13}C-NMR (75.5 MHz, $CDCl_3$): δ 189.0 (C), 140.3 (CH), 138.9 (C), 135.1 (C), 134.1 (C), 130.8 (2 x CH), 128.7 (2 x CH), 15.3 (CH_3). HRMS (ESI$^+$, m/z): calcd. for $C_{10}H_9Cl_2O$ ([M + H]$^+$) 215.0025; found, 215.0031.

(***Z***)**-1-(4-Bromophenyl)-2-chlorobut-2-en-1-one** (**1d**): Pale yellow solid (1.18 g, 91% yield). R_f (Hex:EtOAc 20:1): 0.51. Mp: 62-64 ºC. IR: ν 1768, 1693, 1610, 1479, 1394, 1069, 738, 627 cm^{-1}. ^{1}H-NMR (300.13 MHz, $CDCl_3$): δ 7.59 (quint, *J* = 9.5, 8.9 Hz, 4H), 6.75 (q, *J* = 6.7 Hz, 1H), 2.05 (d, *J* = 6.8 Hz, 3H). ^{13}C-NMR (75.5 MHz, $CDCl_3$): δ 189.7 (C), 141.3 (CH), 135.9 (C), 134.6 (C), 132.1 (2 x CH), 131.3 (2 x CH), 127.9 (C), 16.0 (CH_3). HRMS (ESI$^+$, m/z): calcd. for $C_{10}H_9BrClO$ ([M + H]$^+$) 258.9520; found, 258.9527.

(***Z***)**-2-Chloro-1-(4-fluorophenyl)but-2-en-1-one** (**1e**): White solid (0.92 g, 93% yield). R_f (Hex:EtOAc 20:1): 0.52. Mp: 53-54 ºC. IR: ν 1650, 1479, 1243, 744, 637 cm^{-1}. ^{1}H-NMR (300.13 MHz, $CDCl_3$): δ 7.75 (dd, *J* = 8.7, 5.4 Hz, 2H), 7.14 (t, *J* = 8.6 Hz, 2H), 6.73 (q, *J* = 6.8 Hz, 1H), 2.06 (d, *J* = 6.8 Hz, 3H). ^{13}C-NMR (75.5 MHz, $CDCl_3$): δ 188.8 (C), 165.3 (d, *J* = 254.3 Hz, C), 139.9 (CH), 134.0 (C), 132.9 (*d*, *J* = 3.0 Hz, C), 132.0 (d, *J* = 9.1 Hz, 2 x CH), 115.6 (d, *J* = 22.0 Hz, 2 x CH), 15.4 (CH_3). ^{19}F-RMN (282 MHz, $CDCl_3$): δ –105.80. HRMS (ESI$^+$, m/z): calcd. for $C_{10}H_9ClFO$ ([M + H]$^+$) 199.0320; found, 199.0330.

(***Z***)**-2-Chloro-1-(3-fluorophenyl)but-2-en-1-one** (**1f**): Pale yellow solid (0.94 g, 95% yield). R_f (Hex:EtOAc 20:1): 0.48. Mp: 48-50 ºC. IR: ν 1662, 1582, 1269, 905, 688 cm^{-1}. ^{1}H-NMR (300.13 MHz, CDCl$_3$): δ 7.53–7.33 (m, 3H), 7.30–7.20 (m, 1H), 6.79 (q, *J* = 6.8 Hz, 1H), 2.07 (d, *J* = 6.8 Hz, 3H). ^{13}C-NMR (75.5 MHz, CDCl$_3$): δ 188.8 (C), 162.3 (d, *J* = 248.3 Hz, C), 141.1 (CH), 138.9 (d, *J* = 6.2 Hz, C), 134.2 (C), 130.1 (d, *J* = 7.7 Hz, CH), 125.0 (d, *J* = 2.5 Hz, CH), 119.4 (d, *J* = 21.4 Hz, CH), 116.2 (d, *J* = 22.8 Hz, CH), 15.6 (CH$_3$). ^{19}F-RMN (282 MHz, CDCl$_3$): δ –111.64. HRMS (ESI$^+$, m/z): calcd. for $C_{10}H_9ClFO$ ([M + H]$^+$) 199.0320; found, 199.0327.

(***Z***)**-2-Chloro-1-(2-fluorophenyl)but-2-en-1-one** (**1g**): Bright yellow oil (0.95 g, 96% yield). R_f (Hex:EtOAc 20:1): 0.55. IR: ν 1673, 1451, 1286. 731, 701 cm^{-1}. ^{1}H-NMR (300.13 MHz, CDCl$_3$): δ 7.69 – 7.31 (m, 2H), 7.22 (q, *J* = 7.3 Hz, 1H), 7.11 (t, *J* = 9.1 Hz, 1H), 6.79 (td, *J* = 6.8, 1.4 Hz, 1H), 2.04 (d, *J* = 6.9 Hz, 3H). ^{13}C-NMR (75.5 MHz, CDCl$_3$): δ 187.0 (C), 159.4 (*d*, *J* = 252.0 Hz, C), 142.5 (CH), 135.5 (C), 133.0 (*d*, *J* = 8.1 Hz, CH), 130.6 (CH), 126.1 (*d*, *J* = 15.3 Hz, C), 124.3 (*d*, *J* = 3.0 Hz, CH), 116.3 (*d*, *J* = 21.4 Hz, CH), 15.8 (CH$_3$). ^{19}F-RMN (282 MHz, CDCl$_3$): δ –118.09. HRMS (ESI$^+$, m/z): calcd. for $C_{10}H_9ClFO$ ([M + H]$^+$) 199.0320; found, 199.0322.

III. Synthesis of analytical standards

III.1. Synthesis of racemic chloroketones ***2a-g***

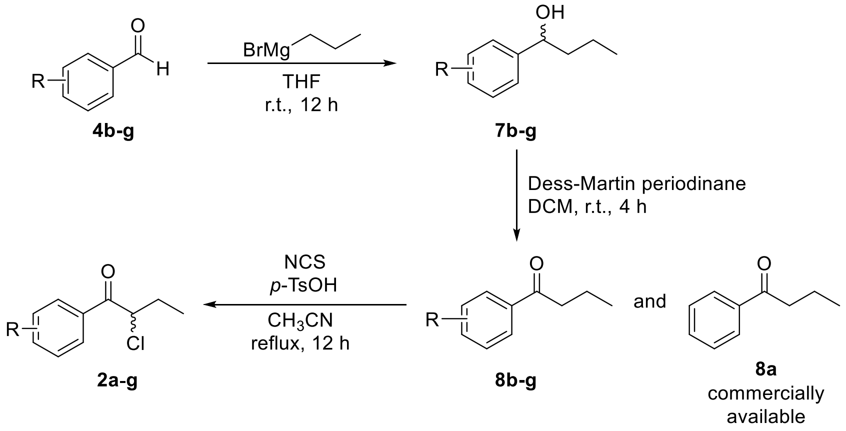

Scheme S3. Synthetic route towards racemic chloroketones **2a-g**.

General procedure for the synthesis of alcohols 7b-g. Propylmagnesium bromide (2 M solution in diethyl ether, 3 mL, 6 mmol, 1.5 equiv.) was added dropwise at 0 ºC to a solution of the corresponding aldehyde **4b-g** (4 mmol, 1 equiv.) in dry THF (13 mL). The resulting solution was stirred at r.t. for 12 h and then quenching on

addition of an aqueous saturated NH_4Cl solution. The mixture was extracted with Et_2O (3 × 20 mL) and the combined organic layers were washed with water (1 x 25 mL) and brine (1 x 25 mL), dried over Na_2SO_4, filtered, and evaporated under reduced pressure. Purification by column chromatography (Hex:EtOAc 10:1) afforded the corresponding 1-arylbutan-1-ols **7b-g**: **7b** (0.54 g, 82% yield), **7c** (0.66 g, 90% yield), **7d** (0.71 g, 77% yield), **7e** (0.53 g, 79% yield), **7f** (0.62 g, 92% yield), and **7g** (0.59 g, 87% yield). Physical and spectral data of **7b-f** were consistent with those reported in the literature.[12] Data for compound **7g** are presented below.

1-(2-Fluorophenyl)butan-1-ol (**7g**): Pale yellow oil (0.59 g, 87% yield). R_f (Hex:EtOAc 10:1): 0.49. IR: ν 3337, 2958, 1617, 1585, 1487, 1220, 1064, 754 cm^{-1}. ^{1}H-NMR (300.13 MHz, $CDCl_3$): δ 7.69–7.31 (m, 2H), 7.22 (q, J = 7.3 Hz, 1H), 7.11 (t, J = 9.1 Hz, 1H), 6.79 (td, J = 6.8, 1.4 Hz, 1H), 2.04 (d, J = 6.9 Hz, 3H). ^{13}C-NMR (75.5 MHz, $CDCl_3$): δ 187.0 (C), 167.1, 157.7 (C), 142.5 (CH), 135.5 (C), 133.0, 132.9 (CH), 130.6 (CH), 126.2, 126.0 (C), 124.3 (CH), 116.4, 116.1 (CH), 15.8 (CH_3). ^{19}F-RMN (282 MHz, $CDCl_3$): δ –118.09.

General procedure for the synthesis of ketones 8b-g. Dess-Martin periodinane (636 mg, 1.5 mmol, 1.5 equiv.) was added to the corresponding solution of alcohol **7b-g** (1 mmol, 1 equiv.) in DCM (3 mL). The reaction mixture was stirred at r.t for 4 h and then diluted with Et_2O (3 mL) and quenched with a 100 mL saturated aqueous $NaHCO_3$ solution containing 25 g of $Na_2S_2O_3$. The mixture was stirred vigorously for 20 minutes and then extracted with DCM (3 × 10 mL). The combined organic layers were successively washed with an aqueous saturated $NaHCO_3$ solution (2 x 10 mL), aqueous saturated $Na_2S_2O_3$ solution (1 x 10 mL), water (1 x 10 mL) and brine (1 x 10 mL), dried over Na_2SO_4, filtered, and evaporated under reduced pressure. Purification by column chromatography (Hex:EtOAc 20:1) afforded the corresponding butyrophenones **8b-g** in 77-92% isolated yield: **8b** (133 mg 82%), **8c** (164 mg, 90%), **8d** (174 mg, 77%), **8e** (131 mg, 79%), **8f** (153 mg, 92%), and **8g** (144 mg, 87%). Physical and spectral data were consistent with those reported in literature.[13]

General procedure for the synthesis of chloroketones 2a-g. To a solution of the corresponding butyrophenone **8a-g** (1 mmol, 1 equiv.) in dry acetonitrile (10 mL), *N*-chlorosuccinimide (NCS, 160 mg, 1.2 mmol, 1.2 equiv.) and *p*-toluenesulfonic acid (*p*-TsOH, 34 mg, 0.2 mmol, 0.2 equiv.) were added, and the mixture was stirred at

reflux for 12 h. After cooling down to r.t., the resulting mixture was filtered through a celite/SiO_2 pad, washing with hexane at 0 ºC (3 x 15 mL). After solvent evaporation under reduced pressure, 1-aryl-2-chlorobutan-1-ones **2a-g** were obtained in 85-95% isolated yield: **2a** (174 mg, 95%), **8b** (187 mg, 95%), **8c** (193 mg, 89%), **8d** (222 mg, 85%), **8e** (183 mg, 91%), **8f** (184 mg, 92%), and **8g** (173 mg, 86%). Physical and spectral data for compound **2a** were consistent with those reported in literature.[14] Physical and spectral data for novel compounds **2b-g** are presented below.

2-Chloro-1-(4-methylphenyl)butan-1-one (**2b**): Pale yellow oil (187 mg, 95% yield). R_f = (Hex:EtOAc 20:1): 0.49. IR: ν 2974, 1684, 1605, 1179, 766 cm^{-1}. ^{1}H-NMR (300.13 MHz, $CDCl_3$): δ 7.88 (d, *J* = 8.2 Hz, 2H), 7.25 (d, *J* = 8.0 Hz, 2H), 5.05 (dd, *J* = 7.8, 5.8 Hz, 1H), 2.38 (s, 3H), 2.23–1.65 (m, 2H), 1.05 (t, *J* = 7.4 Hz, 3H). ^{13}C-NMR (75.5 MHz, $CDCl_3$): δ 193.2 (C), 144.7 (C), 132.0 (C), 129.4 (2 x CH), 128.9 (2 x CH), 59.3 (CH), 27.2 (CH_2), 21.6 (CH_3), 10.8 (CH_3). HRMS (ESI^+, m/z): calcd. for $C_{11}H_{14}ClO$ ($[M + H]^+$) 197.0728; found, 197.0732.

2-Chloro-1-(4-chlorophenyl)butan-1-one (**2c**): Pale yellow oil (193 mg, 89% yield). R_f = (Hex:EtOAc 20:1): 0.51. IR: ν 2997, 1688, 1588, 1276, 764 cm^{-1}. ^{1}H-NMR (300.13 MHz, $CDCl_3$): δ 7.89 (d, *J* = 8.6 Hz, 2H), 7.39 (d, *J* = 8.6 Hz, 2H), 4.96 (dd, *J* = 7.9, 5.7 Hz, 1H), 2.32–1.78 (m, 2H), 1.03 (t, *J* = 7.3 Hz, 3H). ^{13}C-NMR (75.5 MHz, $CDCl_3$): δ 192.3 (C), 140.1 (C), 132.8 (C), 130.3 (2 x CH), 129.0 (2 x CH), 59.2 (CH), 26.9 (CH_2), 10.7 (CH_3). HRMS (ESI^+, m/z): calcd. for $C_{10}H_{10}Cl_2ONa$ ($[M + Na]^+$) 239.0001; found, 239.0010.

1-(4-Bromophenyl)-2-chlorobutan-1-one (**2d**): Colourless oil (222 mg, 85% yield). R_f = (Hex:EtOAc 20:1): 0.51. IR: ν 2973, 1675, 1567, 1296, 902, 742 cm^{-1}. ^{1}H-NMR (300.13 MHz, $CDCl_3$): δ 7.85 (d, *J* = 8.7 Hz, 2H), 7.60 (d, *J* = 8.7 Hz, 2H), 4.97 (dd, *J* = 8.0, 5.7 Hz, 1H), 2.29–1.81 (m, 2H), 1.06 (t, *J* = 7.3 Hz, 3H). ^{13}C-NMR (75.5 MHz, $CDCl_3$): δ 193.0 (C), 133.6 (C), 132.5 (2 x CH), 130.8 (2 x CH), 129.4 (C), 59.5 (CH), 27.3 (CH_2), 11.3 (CH_3). HRMS (ESI^+, m/z): calcd. for $C_{10}H_{10}BrClONa$ ($[M + Na]^+$) 282.9496; found, 282.9501.

2-Chloro-1-(4-fluorophenyl)butan-1-one (**2e**): Pale yellow oil (183 mg, 91% yield). R_f = (Hex:EtOAc 20:1): 0.47. IR: ν 2992, 1689, 1596, 1227, 1158, 853, 764 cm^{-1}. ^{1}H-NMR (300.13 MHz, $CDCl_3$): δ 7.99 (dd, *J* = 8.6, 5.6 Hz, 2H), 7.09 (d, J = 8.6 Hz, 2H),

4.98 (dd, *J* = 7.9, 5.8 Hz, 1H), 2.19–1.82 (m, 2H), 1.02 (t, *J* = 7.4 Hz, 3H). ^{13}C-NMR (75.5 MHz, $CDCl_3$): δ 192.0 (C), 166.0 (d, *J* = 256.0 Hz, C), 131.6 (d, *J* = 9.5 Hz, 2 x CH), 130.9 (d, *J* = 3.1 Hz, C), 115.9 (d, *J* = 22.0 Hz, 2 x CH), 59.2 (CH), 26.9 (CH_2), 10.8 (CH_3). ^{19}F-RMN (282 MHz, $CDCl_3$): δ –103.80. HRMS (ESI$^+$, m/z): calcd. for $C_{10}H_{11}FClO$ ([M + H]$^+$) 201.0477; found, 201.0481.

2-Chloro-1-(3-fluorophenyl)butan-1-one (**2f**): Pale yellow oil (184 mg, 92% yield). R_f = (Hex:EtOAc 20:1): 0.49. IR: ν 2925, 1693, 1588, 1274, 766 cm^{-1}. ^{1}H-NMR (300.13 MHz, $CDCl_3$): δ 7.78 (dd, *J* = 7.7, 1.4 Hz, 1H), 7.68 (dt, *J* = 9.4, 2.1 Hz, 1H), 7.47 (td, *J* = 8.0, 5.5 Hz, 1H), 7.34–7.20 (m, 1H), 4.98 (dd, *J* = 7.9, 5.8 Hz, 1H), 2.32–1.90 (m, 2H), 1.08 (t, *J* = 7.3 Hz, 3H). ^{13}C-NMR (75.5 MHz, $CDCl_3$): δ 192.5 (d, *J* = 2.2 Hz, C), 162.9 (d, *J* = 248.3 Hz, C), 136.7 (d, *J* = 6.4 Hz, C), 130.6 (d, *J* = 7.7 Hz, CH), 124.7 (d, *J* = 3.1 Hz, CH), 120.9 (d, *J* = 21.5 Hz, CH), 115.8 (d, *J* = 22.6 Hz, CH), 59.4 (CH), 27.0 (CH_2), 10.9 (CH_3). ^{19}F-RMN (282 MHz, $CDCl_3$): δ –111.28. HRMS (ESI$^+$, m/z): calcd. for $C_{10}H_{11}FClO$ ([M + H]$^+$) 201.0477; found, 201.0478.

2-Chloro-1-(2-fluorophenyl)butan-1-one (**2g**): Pale yellow oil (173 mg, 86% yield). R_f = (Hex:EtOAc 20:1): 0.49. IR: ν 2985, 1692, 1608, 1480, 762 cm^{-1}. ^{1}H-NMR (300.13 MHz, $CDCl_3$): δ 7.83 (td, *J* = 7.7, 1.6 Hz, 1H), 7.59–7.40 (m, 1H), 7.22 (t, *J* = 7.6 Hz, 1H), 7.11 (dd, *J* = 11.1, 8.7 Hz, 1H), 5.05 (dd, *J* = 7.9, 5.1 Hz, 1H), 2.15 (dt, *J* = 13.6, 7.0 Hz, 1H), 1.91 (dt, *J* = 14.5, 7.3 Hz, 1H), 1.05 (t, *J* = 7.3 Hz, 3H). ^{13}C-NMR (75.5 MHz, $CDCl_3$): δ 192.5 (d, *J* = 4.1 Hz, C), 161.1 (d, *J* = 254.1 Hz, C), 135.1 (d, *J* = 9.2 Hz, CH), 131.4 (d, *J* = 2.2 Hz, CH), 124.8 (d, *J* = 3.2 Hz, C), 123.8 (d, *J* = 12.8 Hz, C), 116.6 (d, *J* = 23.8 Hz, CH), 64.2 (d, *J* = 8.4 Hz, C), 26.9 (CH_2), 10.7 (CH_3). ^{19}F-RMN (282 MHz, $CDCl_3$): δ –110.04. HRMS (ESI$^+$, m/z): calcd. for $C_{10}H_{11}FClO$ ([M + H]$^+$) 201.0477; found, 201.0479.

*III.2. Synthesis of racemic chlorohydrins **3a-g***

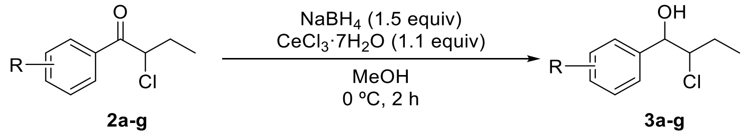

Scheme S4. Synthesis of β-chlorohydrins **3a-g**.

The corresponding α-chloroketone **2a-g** (1 mmol, 1 equiv.) was dissolved in MeOH (5 mL) and cooled down to 0 ºC. Then, $NaBH_4$ (57 mg, 1.5 mmol, 1.5 equiv.) and

$CeCl_3·7H_2O$ (410 mg, 1.1 mmol, 1.1 equiv.) were added to the solution and stirred for 2 h. The reaction was quenched by addition of an aqueous saturated NH_4Cl solution, and the mixture was extracted with EtOAc (3 × 10 mL). The combined organic layers were washed with distilled water (1 x 10 mL) and brine (1 x 10 mL), dried over Na_2SO_4, filtered, and the solvent evaporated under reduced pressure. Purification by column chromatography (Hex:EtOAc 10:1) afforded the corresponding chlorohydrins **3a-g** in 81-99% isolated yields: **3a** (183 mg, 99%), **3b** (189 mg, 95%), **3c** (199 mg, 91%), **3d** (245 mg, 93%), **3e** (190 mg, 94%), **3f** (172 mg, 85%), and **3g** (164 mg, 81%).

IV. Bioreduction of chloroenone 1a to chloroketone 2a

IV.1. Screening of EREDs

Chloroenone **1a** (4.3 mg, 0.025 mmol), 2-PrOH (50 μL), a solution containing D-(+)-glucose (75 mM), GDH-105 (10 U) and NADPH (1 mM) in KPi buffer 100 mM pH 7 (950 μL), and finally ERED-110 (2 mg) or ERED P1-H09 (4 mg) were successively added to a 2 mL-Eppendorf tube. Then, the recipient was closed and kept under orbital shaking at 25 or 30 °C for 24 h and 250 rpm. After this time, the solution was extracted with EtOAc (3 x 0.5 mL) and the combined organic layers were dried over anhydrous Na_2SO_4, filtered and evaporated.

Table S1. Screening of EREDs for the bioreduction of chloroenone **1a**.

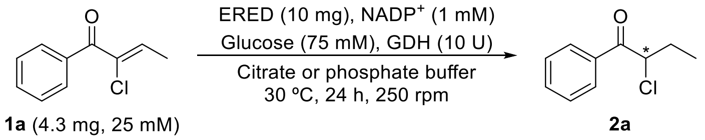

Entry	Buffer	ERED	*c* (%)[a]	*ee* (%)[b]
1	Citrate 100 mM pH 5	103	5	n.d.
2		110	5	n.d.
3		112	<1	n.d.
4		207	6	n.d.
5		P1-A04	27	86 (*R*)
6		P1-E01	6	n.d.
7		P1-H09	9	n.d.
8	Phosphate 100 mM pH 7	103	89	88 (*R*)
9		110	99	94 (*R*)
10		112	7	n.d.
11		207	88	92 (*R*)
12		P1-A04	>99	78 (*R*)
13		P1-E01	72	77 (*R*)
14		P1-H09	78	85 (*S*)

[a] Determined by GC. [c] Determined by HPLC. Configuration of the major enantiomer in parentheses. n.d.: Not determined.

IV.2. Optimization of the ERED-catalyzed process

Table S2. Influence of ERED-110 amount and time in the bioreduction of **1a** using ERED-110.

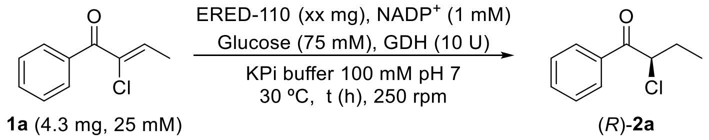

Entry	t (h)	ERED-110 (mg)[a]	*c* (%)	*ee* (%)[b]
1	3	9	96	99
2	4	9	99	99
3	3	7	90	98
4	3	5	75	98
5	3	3	73	96
6	3	2	57	n.d.
7	3	1	10	n.d.

[a] Determined by GC. [b] Determined by HPLC. n.d.: Not determined.

Table S3. Screening of temperature in the bioreduction of **1a** using ERED-110.

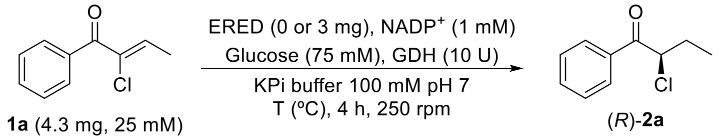

Entry	T (ºC)	ERED-110 (mg)	*c* (%)[a]	*ee* (%)[b]
1	30	3	73	96
2	35	3	73	94
3	40	3	72	92
4	30	0	<1	----

[a] Determined by GC. [b] Determined by HPLC.

Table S4. Influence of the cosolvent in the ERED-100-catalyzed bioreduction of **1a**.

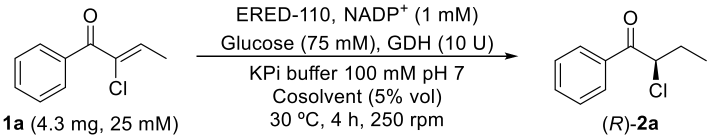

Entry	Cosolvent (5% vol)	ERED-110 (mg)	*c* (%)[a]	*ee* (%)[b]
1	---	3	73	96
2	2-PrOH	3	97	96
3	DMSO	3	69	94
4	THF	3	47	87
5	MeCN	3	52	91
6	2-Me-THF	3	51	92
7	*n*-Heptane	3	19	90
8	TBME	3	65	91
9	2-PrOH	2	98	96
10	2-PrOH	1	97	94

[a] Determined by GC. [b] Determined by HPLC.

Table S5. Influence of cosolvent percentage and temperature in the ERED-catalyzed bioreduction of **1a**.

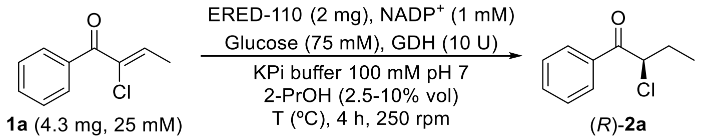

Entry	2-PrOH (% vol)	T (ºC)	*c* (%)[a]	*ee* (%)[b]
1	2.5	30	90	91
2	5	30	97	93
3	7.5	30	83	94
4	10	30	86	94
5	5	25	>99	97

[a] Determined by GC. [b] Determined by HPLC.

Table S6. Monitorization of the ERED-110-catalyzed bioreduction of **1a** over the time.

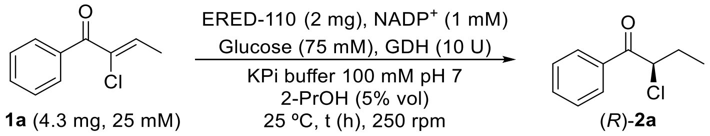

Entry	t (h)	*c* (%)[a]	*ee* (%)[b]
1	4	>99	97
2	3	>99	98
3	2	94	95

[a] Determined by GC. [b] Determined by HPLC.

Table S7. Study of the starting material concentration in the bioreduction of **1a** using ERED-110.

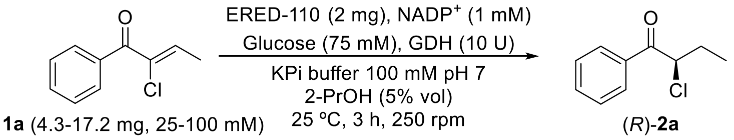

Entry	**1a** (mM)	*c* (%)[a]	*ee* (%)[b]
1	25	>99	98
2	50	56	95
3	75	42	95
4	100	29	92

[a] Determined by GC. [b] Determined by HPLC.

Table S8. Study of the same conditions with the enantiocomplementary enzyme ERED-P1-H09.

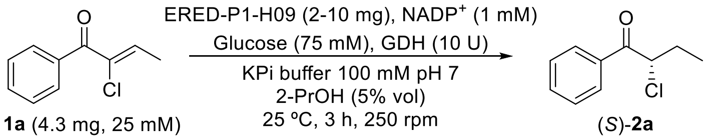

Entry	ERED P1-H09 (mg)	*c* (%)[a]	*ee* (%)[b]
1	2	23	99
2	4	46	99
3	6	50	99
4	8	48	99
5	10	48	99

[a] Determined by GC. [b] Determined by HPLC.

IV.3. Carbonyl bioreduction studies

Table S9. Study of the possible carbonyl bioreduction of substrate **1a** using ADHs.

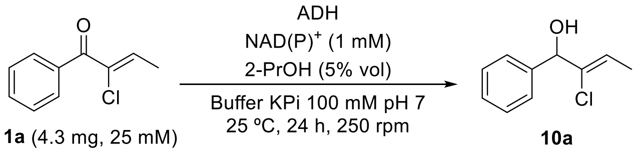

Entry	ADH	*c* (%)[a]
1	evo.1.1.200	82
2	*Lb*ADH	<1
3	*TeS*ADH	<1

[a] Determined by GC.

Table S10. Study of the carbonyl bioreduction of substrate **2a** using ADHs using 2-propanol as coupled substrate.

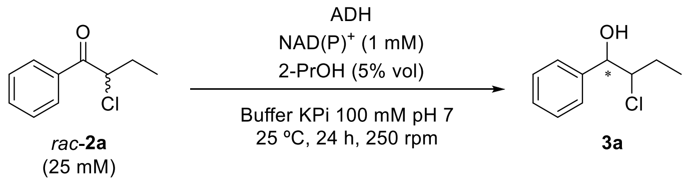

Entry[a]	ADH	***c*** (%)[b]	*syn:anti* ratio[c]	ee_{syn} **(%)**[c]	ee_{anti} (%)[c]
1	*Lb*ADH	35	>99:<1	89 (1*S*,2*S*)	-
2	*TeS*ADH	92	>99:<1	95 (1*S*,2*S*)	-
3	ADH-T	12	>99:<1	n.m.	-
4	ADH-A	13	>99:<1	n.m.	-
5	evo.1.1.200	>99	<1:>99	-	73 (1*S*,2*R*)
6	*Ras*ADH	29	10:90	n.m.	*rac*
7	HLADH	12	>99:<1	n.m	-
8	KRED-P1-A04	93	50:50	*rac*	>99 (1*S*,2*R*)
9	KRED-P1-A12	98	50:50	*rac*	>99 (1*S*,2*R*)
10	KRED-P1-B02	>99	>99:<1	-	20 (1*S*,2*R*)
11	KRED-P1-B05	>99	>99:<1	-	*rac*
12	KRED-P1-B10	>99	>99:<1	-	75 (1*S*,2*R*)
13	KRED-P1-B12	>99	>99:<1	-	75 (1*S*,2*R*)
14	KRED-P1-C01	>99	>99:<1	-	75 (1*S*,2*R*)
15	KRED-P1-H08	>99	>99:<1	-	45 (1*S*,2*R*)
16	KRED-P2-B02	>99	>99:<1	-	*rac*
17	KRED-P2-C02	>99	>99:<1	-	*rac*
18	KRED-P2-C11	>99	>99:<1	-	*rac*
19	KRED-P2-D03	>99	>99:<1	-	70 (1*S*,2*R*)
20	KRED-P2-D11	>99	>99:<1	-	35 (1*S*,2*R*)
21	KRED-P2-D12	>99	>99:<1	-	77 (1*S*,2*R*)
22	KRED-P2-G03	>99	12:88	n.m.	85 (1*S*,2*R*)
23	KRED-P2-H07	91	65:35	*rac*	*rac*
24	KRED-P3-B03	90	91:9	95 (1*S*,2*S*)	n.m.
25	KRED-P3-G09	63	50:50	20 (1*S*,2*S*)	*rac*
26	KRED-P3-H12	83	50:50	27 (1*R*,2*R*)	*rac*

[a] All the experiments were carried using a 25 mM substrate concentration (4.3 mg in 1 mL of solvent) and adding in house *E. coli* enzymes (10 mg), evo.1.1.200 (10 mg) or Codexis KREDs (4 mg).
[b] Conversion values measured by GC analyses.
[c] Enantiomeric and diastereomeric excess values calculated by HPLC analyses.

V. Cascade bioreduction of chloroenones 1a-g to chlorohydrins 3a-g

V.1. Screening of EREDs and ADHs

Table S11. Screening of different EREDs and ADHs for the asymmetric bioreduction of **1a**.

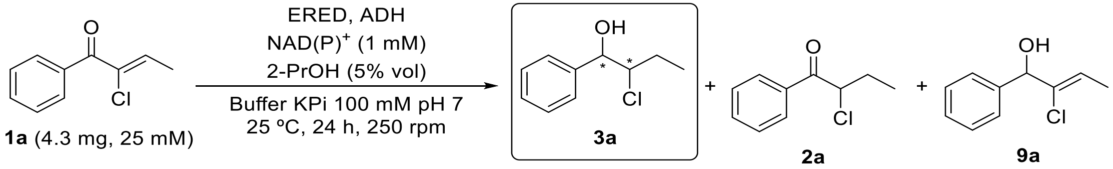

Entry	ERED[a]	ADH[a]	**3a** *c* (%)[b]	**3a** *syn:anti*[c]	*syn*-**3a** *ee* (%)[c]	*anti*-**3a** *ee* (%)[b]	**2a** *c* (%)[b]	**9a** *c* (%)[b]
1	110	*Lb*ADH	>99	99:1	98 (1*R*,2*R*)	n.d.	<1	<1
2	110	TeSADH	98	96:4	98 (1*R*,2*R*)	n.d.	<1	<1
3	110	ADH-T	>99	>99:1	96 (1*R*,2*R*)	n.d.	<1	<1
4	110	ADH-A	>99	98:2	93 (1*R*,2*R*)	n.d.	<1	<1
5	110	evo.1.1.200	>99	<1:>99	n.d.	>99 (1*S*,2*R*)	<1	<1
6	110	*Ras*ADH	59	35:65	95 (1*R*,2*R*)	<1	<1	<1
7	110	HLADH	18	>99:<1	90 (1*R*,2*R*)	n.d.	<1	<1
8	110	KRED-P1-A04	>99	63:37	>99 (1*R*,2*R*)	>99 (1*S*,2*R*)	<1	<1
9	110	KRED-P1-A12	>99	41:59	99 (1*R*,2*R*)	99 (1*S*,2*R*)	<1	<1
10	110	KRED-P1-B02	>99	<1:>99	n.d.	20 (1*R*,2*S*)	<1	<1
11	110	KRED-P1-B05	98	45:55	98 (1*R*,2*R*)	40 (1*S*,2*R*)	<1	<1
12	110	KRED-P1-B10	>99	<1:>99	n.d.	40 (1*S*,2*R*)	<1	<1
13	110	KRED-P1-B12	>99	35:65	>99 (1*R*,2*R*)	72 (1*S*,2*R*)	<1	<1
14	110	KRED-P1-C01	>99	10:90	>99 (1*R*,2*R*)	98 (1*S*,2*R*)	<1	<1
15	110	KRED-P1-H08	>99	10:90	>99 (1*R*,2*R*)	94 (1*S*,2*R*)	<1	<1

Entry	ERED[a]	ADH[a]	**3a** *c* (%)[b]	**3a** *syn:anti*[c]	*syn*-**3a** *ee* (%)[c]	*anti*-**3a** *ee* (%)[b]	**2a** *c* (%)[b]	**9a** *c* (%)[b]
16	110	KRED-P2-B02	>99	<1:>99	n.d.	<1	<1	<1
17	110	KRED-P2-C02	>99	<1:>99	n.d.	10 (1*S*,2*R*)	<1	<1
18	110	KRED-P2-C11	>99	<1:>99	n.d.	<1	<1	<1
19	110	KRED-P2-D03	>99	<1:>99	n.d.	99 (1*S*,2*R*)	<1	<1
20	110	KRED-P2-D11	>99	<1:>99	n.d.	95 (1*S*,2*R*)	<1	<1
21	110	KRED-P2-D12	>99	<1:>99	n.d.	92 (1*S*,2*R*)	<1	<1
22	110	KRED-P2-G03	>99	<1:>99	n.d.	86 (1*S*,2*R*)	<1	<1
23	110	KRED-P2-H07	96	57:42	94 (1*R*,2*R*)	99 (1*S*,2*R*)	<1	<1
24	110	KRED-P3-B03	>99	>99:<1	91 (1*S*,2*S*)	n.d.	<1	<1
25	110	KRED-P3-G09	>99	2:98	n.d.	94 (1*S*,2*R*)	<1	<1
26	110	KRED-P3-H12	>99	>99:<1	92 (1*R*,2*R*)	n.d.	<1	<1
27	P1-H09	*Lb*ADH	>99	>99:<1	85 (1*S*,2*S*)	n.d.	<1	<1
28	P1-H09	TeSADH	91	>99:<1	92 (1*S*,2*S*)	n.d.	<1	9
29	P1-H09	ADH-T	93	>99:<1	83 (1*S*,2*S*)	n.d.	<1	<1
30	P1-H09	ADH-A	37	>99:<1	<1	n.d.	<1	<1
31	P1-H09	evo.1.1.200	40	2:98	n.d.	<1	2	26
32	P1-H09	*Ras*ADH	21	85:15	90 (1*S*,2*S*)	33 (1*S*,2*R*)	<1	30
33	P1-H09	HLADH	10	>99:<1	<1	n.d.	<1	<1
34	P1-H09	KRED-P1-A04	75	58:42	96 (1*S*,2*S*)	>99 (1*S*,2*R*)	<1	25
35	P1-H09	KRED-P1-A12	30	69:31	90 (1*S*,2*S*)	>99 (1*S*,2*R*)	<1	70
36	P1-H09	KRED-P1-B02	90	<1:>99	n.d.	>99 (1*S*,2*R*)	<1	10
37	P1-H09	KRED-P1-B05	60	19:81	98 (1*S*,2*S*)	<1	<1	40
38	P1-H09	KRED-P1-B10	>99	5:95	n.d.	90 (1*S*,2*R*)	<1	<1
39	P1-H09	KRED-P1-B12	74	7:93	n.d.	>99 (1*S*,2*R*)	<1	<1
40	P1-H09	KRED-P1-C01	55	2:98	n.d.	>99 (1*S*,2*R*)	<1	45
41	P1-H09	KRED-P1-H08	20	n.d.	n.d.	n.d.	<1	20

Entry	ERED[a]	ADH[a]	**3a** *c* (%)[b]	**3a** *syn:anti*[c]	*syn*-**3a** *ee* (%)[c]	*anti*-**3a** *ee* (%)[b]	**2a** *c* (%)[b]	**9a** *c* (%)[b]
42	P1-H09	KRED-P2-B02	44	<1:>99	n.d.	>99 (1*S*,2*R*)	7	48
43	P1-H09	KRED-P2-C02	40	<1:>99	n.d.	>99 (1*S*,2*R*)	11	49
44	P1-H09	KRED-P2-C11	14	n.d.	n.d.	n.d.	6	80
45	P1-H09	KRED-P2-D03	54	<1:>99	n.d.	>99 (1*S*,2*R*)	2	18
46	P1-H09	KRED-P2-D11	50	<1:>99	n.d.	>99 (1*S*,2*R*)	<1	14
47	P1-H09	KRED-P2-D12	84	<1:>99	n.d.	>99 (1*S*,2*R*)	<1	16
48	P1-H09	KRED-P2-G03	37	>99:<1	n.d.	>99 (1*S*,2*R*)	10	53
49	P1-H09	KRED-P2-H07	73	71:29	92 (1*S*,2*S*)	>99 (1*R*,2*S*)	10	17
50	P1-H09	KRED-P3-B03	76	73:27	80 (1*S*,2*S*)	>99 (1*R*,2*S*)	3	21
51	P1-H09	KRED-P3-G09	>99	90:10	74 (1*S*,2*S*)	>99 (1*R*,2*S*)	<1	<1
52	P1-H09	KRED-P3-H12	85	86:14	78 (1*S*,2*S*)	>99 (1*R*,2*S*)	<1	<1

[a] Amount of enzyme: ERED-110 (2 mg), ERED-P1-H09 (4 mg), commercial KREDs (4 mg); *E. coli* overexpressed in house enzymes (10 mg). [b] Determined by GC. [c] Determined by HPLC. Configuration of the major enantiomer in parentheses. n.d.: not determined.

During the screening of substrate **1a** in the ERED-ADH bienzymatic cascade experiments, byproduct **9a** was identified. Physical and spectral data are given below:

(*Z*)-2-Chloro-1-phenylbut-2-en-1-ol: (**9a**): Yellow solid. R_f (Hex:EtOAc 10:1): 0.39. IR: ν 3237, 1447, 1013, 698 cm^{-1}. Mp: 71-72 ºC. ^{1}H-NMR (300.13 MHz, $CDCl_3$): δ 7.84–7.06 (*m*, 5H), 6.07–5.84 (*m*, 1H), 5.30 (*s*, 1H), 2.60 (*d*, *J* = 3.8 Hz, 1H), 2.41–1.20 (*m*, 3H). ^{13}C-NMR (75.5 MHz, $CDCl_3$): δ 140.7 (C), 136.8 (C), 128.8 (2 x CH), 128.5 (CH), 126.9 (2 x CH), 123.2 (CH), 77.5 (CH), 14.2 (CH_3). HRMS (ESI^+, m/z): calcd. for $C_{10}H_{11}ClONa$ ($[M + Na]^+$) 205.0391; found, 205.0395.

V.2. General experimental procedures

Experimental procedure using *Ras*ADH, *TeS*ADH, ADH-T and HLADH: Chloroenone **1a** (4.3 mg, 0.025 mmol), 2-PrOH (50 μL, 5% vol), a NADPH 10 mM aqueous solution (50 μL), KPi buffer 100 mM pH 7 (900 μL), ERED-110 (2 mg) or ERED P1-H09 (4 mg) and lyophilized cells of *E. coli* overexpressing ADH (10 mg) were successively added to a 2 mL-Eppendorf tube. Then, the recipient was closed and kept under orbital shaking at 250 rpm at 25 °C for 24 h. After this time, the solution was extracted with EtOAc (3 x 0.5 mL), and the combined organic layers were dried over anhydrous Na_2SO_4, filtered and evaporated under reduced pressure.

Experimental procedure using *Ras*ADH and 2-PrOH: Chloroenone **1a** (4.3 mg, 0.025 mmol), 2-PrOH (25 μL, 2.5% vol), a solution containing D-(+)-glucose (75 mM), GDH-105 (10 U) and NADPH (1 mM) in KPi buffer 100 mM pH 7 (975 μL), ERED P1-H09 (4 mg) and lyophilized cells of *E. coli* overexpressing *Ras*ADH (10 mg) were successively added to a 2 mL-Eppendorf tube. Then, the recipient was closed and kept under orbital shaking at 250 rpm at 25 °C for 24 h. After this time, the solution was extracted with EtOAc (3 x 0.5 mL), and the combined organic layers were dried over anhydrous Na_2SO_4, filtered and evaporated under reduced pressure.

Experimental procedure using using *Lb*ADH: Chloroenone **1a** (4.3 mg, 0.025 mmol), 2-PrOH (50 μL, 5% vol), a NADPH 10 mM aqueous solution (50 μL), a $MgCl_2$ 10 mM aqueous solution (50 μL), KPi buffer 100 mM pH 7 (850 μL), ERED-110 (2 mg) or ERED P1-H09 (4 mg) and lyophilized cells of *E. coli* overexpressing *Lb*ADH (10 mg) were successively added to a 2 mL-Eppendorf tube. Then, the recipient was closed and

kept under orbital shaking at 250 rpm at 25 °C for 24 h. After this time, the solution was extracted with EtOAc (3 x 0.5 mL), and the combined organic layers were dried over anhydrous Na_2SO_4, filtered and evaporated under reduced pressure.

Experimental procedure using ADH-A: Chloroenone **1a** (4.3 mg, 0.025 mmol), 2-PrOH (50 μL, 5% vol), a NADH 10 mM aqueous solution (25 μL), a NADPH 10 mM aqueous solution (25 μL), KPi buffer 100 mM pH 7 (900 μL), ERED-110 (2 mg) or ERED P1-H09 (4 mg) and lyophilized cells of *E. coli* overexpressing ADH-A (10 mg) were successively added to a 2 mL-Eppendorf tube. Then, the recipient was closed and kept under orbital shaking at 250 rpm at 25 °C for 24 h. After this time, the solution was extracted with EtOAc (3 x 0.5 mL), and the combined organic layers were dried over anhydrous Na_2SO_4, filtered and evaporated under reduced pressure.

Experimental procedure using commercial ADHs from Codexis: The selected commercially available Codexis KRED (4 mg) was added to a 2 mL Eppendorf tube containing chloroenone **1a** (4.3 mg, 0.025 mmol), 2-PrOH (50 μL, 5% vol), a NADH 10 mM aqueous solution (25 μL), a NADPH 10 mM aqueous solution (25 μL), a $MgCl_2$ 10 mM aqueous solution (50 μL), KPi buffer 100 mM pH 7 (850 μL) and ERED-110 (2 mg) or ERED P1-H09 (4 mg). Then, the recipient was closed and kept under orbital shaking at 250 rpm at 25 °C for 24 h. After this time, the solution was extracted with EtOAc (3 x 0.5 mL), and the combined organic layers were dried over anhydrous Na_2SO_4, filtered and evaporated under reduced pressure.

Experimental procedure using commercial evo.1.1.200: evo.1.1.200 (4 mg) was added to a 2 mL Eppendorf tube containing chloroenone **1a** (4.3 mg, 0.025 mmol), 2-PrOH (50 μL, 5% vol), a NADH 10 mM aqueous solution (25 μL), a NADPH 10 mM aqueous solution (25 μL), a $MgCl_2$ 10 mM aqueous solution (50 μL), KPi buffer 100 mM pH 7 (850 μL) and ERED-110 (2 mg) or ERED P1-H09 (4 mg). Then, the recipient was closed and kept under orbital shaking at 250 rpm at 25 °C for 24 h. After this time, the solution was extracted with EtOAc (3 x 0.5 mL), and the combined organic layers were dried over anhydrous Na_2SO_4, filtered and evaporated under reduced pressure.

*V.3. Assignment of the absolute configuration of chlorohydrin **1a***

The absolute configuration of ketone intermediate (*R*)-**2a** obtained in the bioreduction of **1a** by ERED-110 was previously assigned by comparison with the data reported in literature.[14] Thus, for the assignment of the other chiral center, the product resulting from the ERED-110 and evo.1.1.200 cascade **3a** was subjected to a chemical epoxidation to analyze the *cis*/*trans* disposition of its substituents (Scheme S5), and therefore, unambiguously assign the absolute configuration of all the different **3a** diastereoisomers through analysis of the ^{1}H-NMR crudes. As data of both *cis* and *trans* isomers of epoxide **10a** has been reported in the literature,[16] the assignment of the relative configuration of the β-chlorohydrin obtained in this process could be made by comparison of the coupling constants observed in the ^{1}H-NMR spectrum (δ 3.65 (*d*, *J* = 1.9 Hz, 1H), 2.98 (*td*, *J* = 5.5, 2.1 Hz, 1H)), resulting to be the *trans* diastereoisomer. Combining this fact with previous knowledge of the disposition of the carbon chlorine bond, the absolute configuration could be unambiguously assigned, being (1*S*,2*R*). The rest of the compounds could be assigned *via* HPLC by comparison of the peak order in the same columns and ^{1}H-NMR.

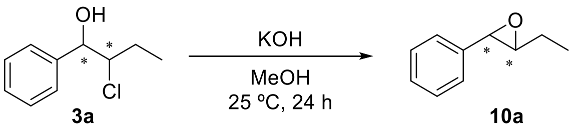

Scheme S5. Epoxidation of chlorohydrin **3a** to determine its configuration.

To a solution of β-chlorohydrin **3a** (12.6 mg, 0.07 mmol, 1 equiv.) in MeOH (1 mL) KOH was added (11.8 mg, 0.21 mmol, 3 equiv.) and the mixture was stirred at r.t. for 24. Then, the reaction mixture was diluted with water (5 mL) and extracted with EtOAc (3 x 5 mL). The combined organic layers were dried over anhydrous Na_2SO_4, filtered and evaporated under reduced pressure. ^{1}H-NMR of the crude residue allowed the determination of the absolute configuration (see Figure S1).

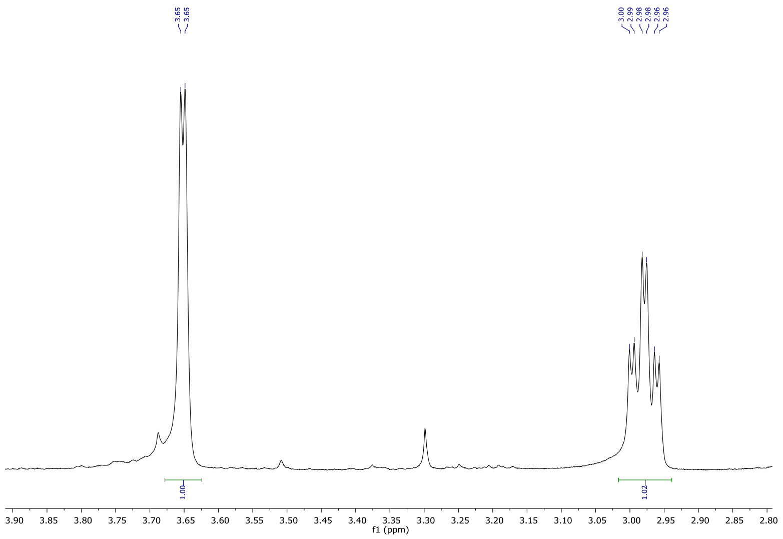

Figure S1. Area of interest in ^{1}H-NMR spectra of epoxide **10a.**

*V.4. Scope of the cascade bienzymatic reduction of chloroenones **1b-g***

Table S12. Screening of different ADHs for the asymmetric bioreduction of **1b**.

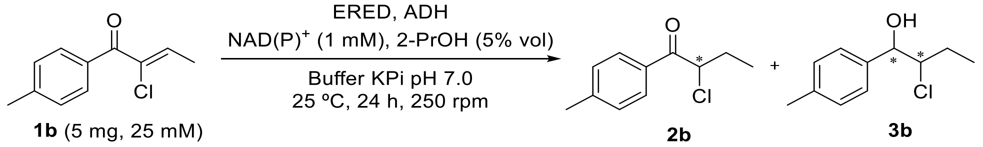

Entry	ERED[a]	ADH[a]	**3b** *c* (%)[b]	*syn:anti*[c]	**2b** *c* (%)[b]	**2b** *ee* (%)[c]	*syn*-**3b** *ee* (%)[c]	*anti*-**3b** *ee* (%)[c]
1	110	evo.1.1.200	>99	99:1	<1	n.d.	>99 (1*R*,2*R*)	n.d.
2	110	*Lb*ADH	<1	n.d.	>99	>99 (*R*)	n.d.	n.d.
3	110	TeSADH	<1	n.d.	95	>99 (*R*)	n.d.	n.d.
4	P1-H09	*Lb*ADH	<1	n.d.	>99	93 (*S*)	n.d.	n.d.
5	P1-H09	TeSADH	<1	n.d.	96	94 (*S*)	n.d.	n.d.
6	P1-H09	*Ras*ADH[d]	92	1:>99	<1	n.d.	n.d.	>99 (1*R*,2*S*)

[a] Amount of enzyme: ERED-110 (2 mg), ERED-P1-H09 (4 mg), evo.1.1.200 (4 mg); *E. coli* overexpressed in house enzymes (10 mg). [b] Determined by GC. [c] Determined by HPLC. Configuration of the major enantiomer in parentheses. Absolute configuration was assigned based on the results obtained for **3a**. [d] 2.5% of 2-PrOH and glucose (75 mM)/GDH (10 U).

Table S13. Screening of different ADHs for the asymmetric bioreduction of **1c**.

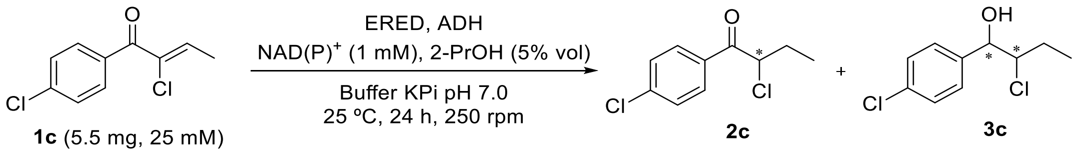

Entry	ERED[a]	ADH[a]	**3c** *c* (%)[b]	*syn:anti*[c]	**2c** *c* (%)[b]	**2c** *ee* (%)[c]	*syn*-**3c** *ee* (%)[c]	*anti*-**3c** *ee* (%)[c]
1	110	evo.1.1.200	>99	98:2	<1	n.d.	>99 (1*R*,2*R*)	n.d.
2	110	*Lb*ADH	6	n.d.	94	96 (*R*)	n.d.	n.d.
3	110	TeSADH	<1	n.d.	77	98 (*R*)	n.d.	n.d.
4	P1-H09	*Lb*ADH	5	n.d.	95	80 (*S*)	n.d.	n.d.
5	P1-H09	TeSADH	<1	n.d.	83	85 (*S*)	n.d.	n.d.
6	P1-H09	evo.1.1.200	98	12:88	n.d.	n.d.	34 (1*S*,2*S*)	66 (1*R*,2*S*)
7	P1-H09	*Ras*ADH[d]	>99	10:90	<1	n.d.	>99 (1*R*,2*R*)	>99 (1*S*,2*R*)

[a] Amount of enzyme: ERED-110 (2 mg), ERED-P1-H09 (4 mg), evo.1.1.200 (4 mg); *E. coli* overexpressed in house enzymes (10 mg). [b] Determined by GC. [c] Determined by HPLC. Configuration of the major enantiomer in parentheses. Absolute configuration was assigned based on the results obtained for **3a**. [d] 2.5% of 2-PrOH and glucose (75 mM)/GDH (10 U).

Table S14. Screening of different ADHs for the asymmetric bioreduction of **1d**.

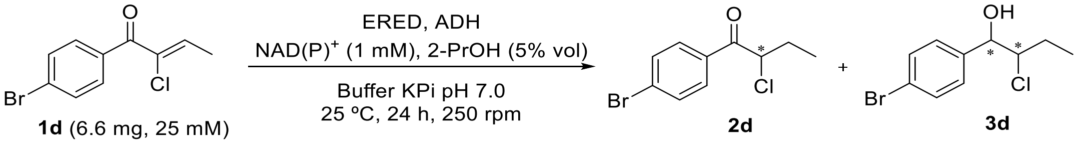

Entry	ERED[a]	ADH[a]	**3d** *c* (%)[b]	*syn:anti*[c]	**2d** *c* (%)[b]	**2d** *ee* (%)[c]	*syn*-**3d** *ee* (%)[c]	*anti*-**3d** *ee* (%)[c]
1	110	evo.1.1.200	>99	>99:1	<1	n.d.	>99 (1*R*,2*R*)	n.d.
2	110	*Lb*ADH	8	n.d.	92	98 (*R*)	n.d.	n.d.
3	110	TeSADH	6	n.d.	94	93 (*R*)	n.d.	n.d.
4	P1-H09	*Lb*ADH	4	n.d.	96	72 (*S*)	n.d.	n.d.
5	P1-H09	TeSADH	11	n.d.	89	86 (*S*)	n.d.	n.d.
6	P1-H09	evo.1.1.200	51	18:82	49	86 (*S*)	50 (1*S*,2*S*)	40 (1*R*,2*S*)
7	P1-H09	*Ras*ADH[d]	>99	6:94	<1	n.d.	>99 (1*R*,2*R*)	>99 (1*S*,2*R*)

[a] Amount of enzyme: ERED-110 (2 mg), ERED-P1-H09 (4 mg), evo.1.1.200 (4 mg); *E. coli* overexpressed in house enzymes (10 mg). [b] Determined by GC. [c] Determined by HPLC. Configuration of the major enantiomer in parentheses. Absolute configuration was assigned based on the results obtained for **3a**. [d] 2.5% of 2-PrOH and glucose (75 mM)/GDH (10 U).

Table S15. Screening of different ADHs for the asymmetric bioreduction of **1e**.

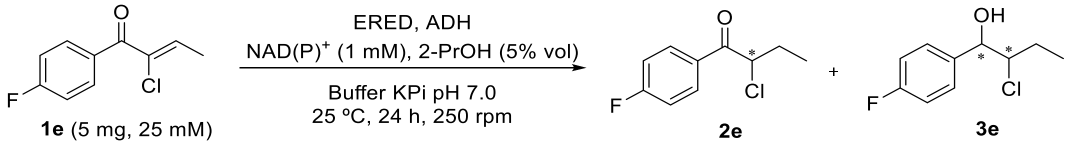

Entry	ERED[a]	ADH[a]	**3e** *c* (%)[b]	*syn:anti*[c]	**2e** *c* (%)[b]	**2e** *ee* (%)[c]	*syn*-**3e** *ee* (%)[c]	*anti*-**3e** *ee* (%)[c]
1	110	evo.1.1.200	>99	93:7	<1	n.d.	>99 (1*S*,2*S*)	n.d.
2	110	*Lb*ADH	5	n.d.	95	96 (*R*)	n.d.	n.d.
3	110	TeSADH	5	n.d.	95	75 (*R*)	n.d.	n.d.
4	P1-H09	*Lb*ADH	9	n.d.	83	86 (*S*)	n.d.	n.d.
5	P1-H09	TeSADH	>99	86:14	<1	n.d.	>99 (1*S*,2*S*)	>99 (1*S*,2*R*)
6	P1-H09	evo.1.1.200	86	9:91	n.d.	n.d.	n.d.	98 (1*R*,2*S*)
7	P1-H09	*Ras*ADH[d]	>99	50:50	<1	n.d.	>99 (1*S*,2*S*)	>99 (1*S*,2*R*)

[a] Amount of enzyme: ERED-110 (2 mg), ERED-P1-H09 (4 mg), evo.1.1.200 (4 mg); *E. coli* overexpressed in house enzymes (10 mg). [b] Determined by GC. [c] Determined by HPLC. Configuration of the major enantiomer in parentheses. Absolute configuration was assigned based on the results obtained for **3a**. [d] 2.5% of 2-PrOH and glucose (75 mM)/GDH (10 U).

Table S16. Screening of different ADHs for the asymmetric bioreduction of **1f**.

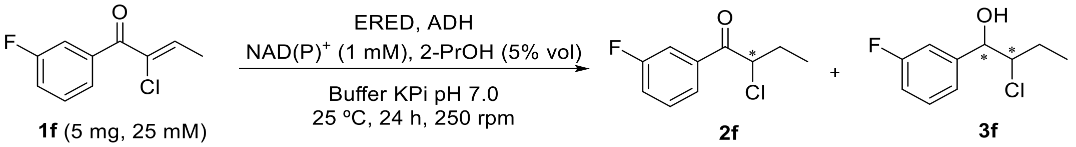

Entry	ERED[a]	ADH[a]	**3f** *c* (%)[b]	*syn:anti*[c]	**2f** *c* (%)[b]	**2f** *ee* (%)[c]	*syn*-**3f** *ee* (%)[c]	*anti*-**3f** *ee* (%)[c]
1	110	evo.1.1.200	>99	91:9	<1	n.d.	>99 (1*R*,2*R*)	n.d.
2	110	*Lb*ADH	11	n.d.	89	>99 (*R*)	n.d.	n.d.
3	110	TeSADH	24	70:30	76	>99 (*R*)	>99 (1*R*,2*R*)	>99 (1*R*,2*S*)
4	P1-H09	*Lb*ADH	16	>1:99	84	90 (*S*)	n.d.	40 (1*R*,2*S*)
5	P1-H09	TeSADH	19	>1:99	81	90 (*S*)	n.d.	>99 (1*R*,2*S*)
6	P1-H09	evo.1.1.200	>99	4:96	n.d.	n.d.	n.d.	94 (1*S*,2*R*)
7	P1-H09	*Ras*ADH[d]	>99	40:60	<1	n.d.	>99 (1*S*,2*S*)	66 (1*R*,2*S*)

[a] Amount of enzyme: ERED-110 (2 mg), ERED-P1-H09 (4 mg), evo.1.1.200 (4 mg); *E. coli* overexpressed in house enzymes (10 mg). [b] Determined by GC. [c] Determined by HPLC. Configuration of the major enantiomer in parentheses. Absolute configuration was assigned based on the results obtained for **3a**. [d] 2.5% of 2-PrOH and glucose (75 mM)/GDH (10 U).

Table S17. Screening of different ADHs for the asymmetric bioreduction of **1g**.

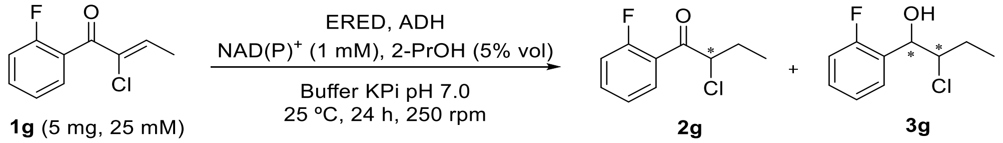

Entry	ERED[a]	ADH[a]	**3g** *c* (%)[b]	*syn:anti*[c]	**2g** *c* (%)[b]	**2g** *ee* (%)[c]	*syn*-**3g** *ee* (%)[c]	*anti*-**3g** *ee* (%)[c]
1	110	evo.1.1.200	>99	93:7	>1	n.d.	>99 (1*R*,2*R*)	n.d.
2	110	*Lb*ADH	12	n.d.	88	36 (*R*)	n.d.	n.d.
3	110	TeSADH	4	n.d.	96	62 (*R*)	n.d.	n.d.
4	P1-H09	*Lb*ADH	6	n.d.	94	34 (*S*)	n.d.	n.d.
5	P1-H09	TeSADH	5	n.d.	95	44 (*S*)	n.d.	n.d.
6	P1-H09	evo.1.1.200	>99	64:36	n.d.	n.d.	>99 (1*R*,2*R*)	90 (1*S*,2*R*)
7	P1-H09	*Ras*ADH[d]	>99	3:97	>1	n.d.	n.d.	>99 (1*S*,2*R*)

[a] Amount of enzyme: ERED-110 (2 mg), ERED-P1-H09 (4 mg), evo.1.1.200 (4 mg); *E. coli* overexpressed in house enzymes (10 mg). [b] Determined by GC. [c] Determined by HPLC. Configuration of the major enantiomer in parentheses. Absolute configuration was assigned based on the results obtained for **3a**. [d] 2.5% of 2-PrOH and glucose (75 mM)/GDH (10 U).

***syn*-2-Chloro-1-phenylbutan-1-ol (*syn*-3a):** Pale yellow oil [(1*R*,2*R*): 18 mg, 87% yield), ((1*S*,2*S*): 17 mg, 84% yield]. R_f 0.29 (Hex:EtOAc 10:1). (1*R*,2*R*): $[\alpha]^{20}_D$ = +61.5 (0.1 *c*, $CHCl_3$); (1*S*,2*S*): $[\alpha]^{20}_D$ = -56.4 (0.1 *c*, $CHCl_3$). IR: ν 3393, 2972, 1453, 792, 699 cm^{-1}. ^{1}H-NMR (300.13 MHz, $CDCl_3$): δ 8.38–6.75 (m, 5H), 4.95 (s, 1H), 4.43–3.95 (m, 1H), 2.51 (s, 1H), 2.08–1.53 (m, 2H), 1.02 (t, *J* = 7.2 Hz, 3H). ^{13}C-NMR (75.5 MHz, $CDCl_3$): δ 139.9 (C), 128.3 (2 x CH), 128.0 (CH), 126.6 (2 x CH), 76.9 (CH), 70.2 (CH), 24.8 (CH_2), 11.3 (CH_3). HRMS (ESI^+, m/z): calcd. for $C_{10}H_{13}{}^{35}ClONa$ ([M + Na]$^+$) 207.0547; found, 207.0547.

***anti*-2-Chloro-1-phenylbutan-1-ol (*anti*-3a):** Pale yellow oil (148 mg, 81% yield). R_f (Hex:EtOAc 10:1): 0.28. (1*S*,2*R*): $[\alpha]^{20}_D$ = -26.5 (0.1 *c*, $CHCl_3$). IR: ν 3380, 2972, 1452, 763, 699 cm^{-1}. ^{1}H-NMR (300.13 MHz, $CDCl_3$): δ 7.35 (d, *J* = 7.4 Hz, 5H), 4.67 (d, *J* = 7.1 Hz, 1H), 4.05 (ddd, *J* = 9.0, 7.2, 4.0 Hz, 1H), 2.78 (s, 1H), 2.05-1.39 (m, 2H), 1.03 (t, *J* = 7.3 Hz, 3H). ^{13}C-NMR (75.5 MHz, $CDCl_3$): δ 140.1 (C), 128.6 (2 x CH), 128.4 (CH), 126.8 (2 x CH), 77.4 (CH), 71.8 (CH), 27.5 (CH_2), 11.1 (CH_3). HRMS (APCI+, m/z): calcd. for $C_{10}H_{12}{}^{35}Cl$ ([M – OH]$^+$) 167.0622; found, 167.0619.

***syn*-2-Chloro-1-(4-methylphenyl)butan-1-ol (*syn*-3b):** Pale yellow oil (18 mg, 89% yield). R_f (Hex:EtOAc 10:1): 0.30. (1*R*,2*R*): $[\alpha]^{20}_D$= +40.1 (0.1 *c*, $CHCl_3$); IR: ν 3207, 2933, 1454, 764, 750 cm^{-1}. ^{1}H-NMR (300.13 MHz, $CDCl_3$): δ 7.29 (d, *J* = 7.7 Hz, 2H),

7.20 (d, *J* = 7.9 Hz, 2H), 4.93 (d, *J* = 4.3 Hz, 1H), 4.11 (dt, *J* = 9.8, 3.7 Hz, 1H), 2.38 (s, 4H), 2.08-1.50 (m, 2H), 1.03 (t, *J* = 7.3 Hz, 3H). ^{13}C-NMR (75.5 MHz, $CDCl_3$): δ 136.9 (C), 130.9 (C), 129.0 (2 x CH), 126.5 (2 x CH), 76.8 (CH), 70.2 (CH), 24.8 (CH_2), 21.2 (CH_3), 11.3 (CH_3). HRMS (APCI+, m/z): calcd. for $C_{11}H_{14}{}^{35}Cl$ ([M – OH]$^+$) 181.0779; found, 181.0774.

***anti*-2-Chloro-1-(4-methylphenyl)butan-1-ol (*anti*-3b):** Pale yellow oil (16 mg, 80% yield). R_f (Hex:EtOAc 10:1): 0.33. (1*R*,2*S*): $[\alpha]^{20}_D$ = +27.9 (0.1 *c*, $CHCl_3$). IR: ν 3414, 2923, 1461, 765, 750 cm^{-1}. ^{1}H-NMR (300.13 MHz, $CDCl_3$): δ 7.25 (d, *J* = 7.9 Hz, 2H), 7.18 (d, *J* = 7.7 Hz, 2H), 4.64 (d, *J* = 7.1 Hz, 1H), 4.04 (ddd, *J* = 9.0, 7.2, 3.8 Hz, 1H), 2.83 (s, 1H), 2.36 (s, 3H), 2.05-1.49 (m, 2H), 1.02 (*t*, *J* = 7.3 Hz, 3H). ^{13}C-NMR (75.5 MHz, $CDCl_3$): δ 138.2 (C), 137.1 (C), 129.3 (2 x CH), 126.8 (2 x CH), 77.4 (CH), 72.1 (CH), 27.6 (CH_2), 21.3 (CH_3), 11.2 (CH_3). HRMS (APCI+, m/z): calcd. for $C_{11}H_{14}{}^{35}Cl$ ([M – OH]$^+$) 181.0779; found, 181.0774.

***syn*-2-Chloro-1-(4-chlorophenyl)butan-1-ol (*syn*-3c):** Colorless oil (16 mg, 81% yield). R_f (Hex:EtOAc 10:1): 0.21. (1*R*,2*R*): $[\alpha]^{20}_D$ = +29.3 (0.1 *c*, $CHCl_3$). IR: ν 3304, 2964, 1491, 1089, 792, 593 cm^{-1}. ^{1}H-NMR (300.13 MHz, $CDCl_3$): δ 7.38–7.28 (m, 4H), 4.92 (s, 1H), 4.06 (dd, *J* = 5.7, 4.0 Hz, 1H), 2.48 (s, 1H), 1.85-1.61 (m, 2H), 1.00 (t, *J* = 7.3 Hz, 3H). ^{13}C-NMR (75.5 MHz, $CDCl_3$): δ 138.9 (C), 138.4 (C), 128.5 (2 x CH), 128.0 (2 x CH), 76.3 (CH), 69.9 (CH), 24.7 (CH_2), 11.2 (CH_3). HRMS (APCI+, m/z): calcd. for $C_{10}H_{11}{}^{35}Cl_2$ ([M – OH]$^+$) 201.0232; found, 201.0232.

***anti*-2-Chloro-1-(4-chlorophenyl)butan-1-ol (*anti*-3c):** Colorless oil (17 mg, 86% yield). R_f (Hex:EtOAc 10:1): 0.25. (1*S*,2*R*): $[\alpha]^{20}_D$ = -18.2 (0.1 *c*, $CHCl_3$). IR: ν 3421, 2970, 1491, 1087, 799, 599 cm^{-1}. ^{1}H-NMR (300.13 MHz, $CDCl_3$): δ 7.51–7.24 (m, 4H), 4.66 (d, *J* = 6.8 Hz, 1H), 3.98 (ddd, *J* = 9.1, 6.8, 4.0 Hz, 1H), 2.89 (s, 1H), 1.86-1.50 (m, 2H), 1.02 (t, *J* = 7.2 Hz, 3H). ^{13}C-NMR (75.5 MHz, $CDCl_3$): δ 138.7 (C), 134.2 (C), 128.8 (2 x CH), 128.3 (2 x CH), 76.7 (CH), 71.6 (CH), 27.6 (CH_2), 11.2 (CH_3). HRMS (APCI+, m/z): calcd. for $C_{10}H_{11}{}^{35}Cl_2$ ([M – OH]$^+$) 201.0232; found, 201.0223.

***syn*-1-(4-Bromophenyl)-2-chlorobutan-1-ol (*syn*-3d):** Colorless oil (17 mg, 85% yield). R_f (Hex:EtOAc 10:1): 0.24. (1*R*,2*R*): $[\alpha]^{20}_D$ = +71.3 (0.1 *c*, $CHCl_3$). IR: ν 3092, 2971, 1487, 1009, 725 cm^{-1}. ^{1}H-NMR (300.13 MHz, $CDCl_3$): δ 7.52 (d, *J* = 8.4 Hz, 2H), 7.26 (d, *J* = 8.3 Hz, 2H), 4.93 (s, 1H), 4.08 (dt, *J* = 9.6, 3.9 Hz, 1H), 2.51 (s, 1H),

1.69 (ttd, *J* = 17.4, 10.1, 8.7, 5.8 Hz, 2H), 1.03 (t, *J* = 7.3 Hz, 3H). ^{13}C-NMR (75.5 MHz, $CDCl_3$): δ 138.8 (C), 131.5 (2 x CH), 128.3 (2 x CH), 122.0 (C), 76.3 (CH), 69.8 (CH), 24.7 (CH_2), 11.2 (CH_3). HRMS (APCI+, m/z): calcd. for $C_{10}H_{11}{}^{35}ClBr$ ([M – OH]$^+$) 244.9727; found, 244.9728.

***anti*-1-(4-Bromophenyl)-2-chlorobutan-1-ol (*anti*-3d):** Colorless oil (18 mg, 90% yield). R_f (Hex:EtOAc 10:1): 0.28. (1*S*,2*R*): $[\alpha]^{20}_D$= -62.5 (0.1 *c*, $CHCl_3$). IR: ν 3446, 2969, 1486, 1010, 722 cm^{-1}. ^{1}H-NMR (300.13 MHz, $CDCl_3$): δ 7.51 (d, *J* = 8.4 Hz, 2H), 7.24 (d, *J* = 8.4 Hz, 2H), 4.65 (d, *J* = 6.7 Hz, 1H), 3.99 (ddd, *J* = 9.2, 6.9, 4.1 Hz, 1H), 2.95 (s, 1H), 1.74–1.55 (m, 2H), 1.03 (t, *J* = 7.2 Hz, 3H). ^{13}C-NMR (75.5 MHz, $CDCl_3$): δ 139.4 (C), 132.1 (2 x CH), 128.9 (2 x CH), 122.7 (C), 77.1 (CH), 71.9 (CH), 27.9 (CH_2), 11.6 (CH_3). HRMS (APCI+, m/z): calcd. for $C_{10}H_{11}{}^{35}ClBr$ ([M – OH]$^+$) 244.9727; found, 244.9714.

***syn*-2-Chloro-1-(4-fluorophenyl)butan-1-ol (*syn*-3e):** Colorless oil (17 mg, 85% yield). R_f (Hex:EtOAc 10:1): 0.22. (1*S*,2*S*): $[\alpha]^{20}_D$ = -114.2 (0.1 *c*, $CHCl_3$). IR: ν 3428, 2981, 1604, 1509, 1221 cm^{-1}. ^{1}H-NMR (300.13 MHz, $CDCl_3$): δ 7.36 (dd, *J* = 8.4, 5.5 Hz, 2H), 7.05 (t, *J* = 8.7 Hz, 2H), 4.92 (s, 1H), 4.06 (dt, *J* = 9.8, 3.8 Hz, 1H), 2.46 (d, *J* = 3.0 Hz, 1H), 1.84–1.64 (m, , 2H), 1.01 (t, *J* = 7.3 Hz, 3H). ^{13}C-NMR (75.5 MHz, $CDCl_3$): δ 160.8 (C), 135.6 (C), 128.3 (d, *J* = 3.0 Hz, 2 x CH), 115.2 (d, *J* = 21.5 Hz, 2 x CH), 76.3 (CH), 70.0 (CH), 24.8 (CH_2), 11.2 (CH_3). ^{19}F-RMN (282 MHz, $CDCl_3$): δ –114.15. HRMS (APCI+, m/z): calcd. for $C_{10}H_{11}{}^{35}ClF$ ([M – OH]$^+$) 185.0528; found, 185.0526.

***anti*-2-Chloro-1-(4-fluorophenyl)butan-1-ol (*anti*-3e):** Colorless oil (16 mg, 80% yield). R_f (Hex:EtOAc 10:1): 0.22. (1*R*,2*S*): $[\alpha]^{20}_D$= +23.5 (0.1 *c*, $CHCl_3$). IR: ν 3392, 2977, 1602, 1511, 1225 cm^{-1}. ^{1}H-NMR (300.13 MHz, $CDCl_3$): δ 7.34 (dd, *J* = 8.7, 5.4 Hz, 2H), 7.06 (t, *J* = 8.6 Hz, 2H), 4.67 (*d*, *J* = 7.1 Hz, 1H), 4.00 (ddd, *J* = 9.2, 7.2, 3.8 Hz, 1H), 2.8 (s, 1H), 1.69–1.57 (m,2H), 1.03 (t, *J* = 7.3 Hz, 3H). ^{13}C-NMR (75.5 MHz, $CDCl_3$): δ 162.6 (d, *J* = 246.9 Hz, C), 135.7 (C), 128.5 (d, *J* = 7.1 Hz, 2 x CH), 115.5 (d, *J* = 21.4 Hz, 2 x CH), 76.7 (CH), 71.8 (CH), 27.4 (CH_2), 11.0 (CH_3). ^{19}F-RMN (282 MHz, $CDCl_3$): δ –113.63. HRMS (APCI+, m/z): calcd. for $C_{10}H_9{}^{35}ClFO$ ([M – 3H]$^+$) 199.0320; found, 199.0320.

syn*-2-Chloro-1-(3-fluorophenyl)butan-1-ol** (syn*-3f**): Colorless oil (16 mg, 80% yield). R_f (Hex:EtOAc 10:1): 0.17. (1*R*,2*R*): $[\alpha]^{20}_{D}$ = +57.5 (0.1 *c*, $CHCl_3$). IR: ν 3330, 2989, 1590, 1137, 756 cm^{-1}. ^1H-NMR (300.13 MHz, $CDCl_3$): δ 7.43–7.28 (m, 1H), 7.21–7.07 (m, 2H), 7.06 – 6.93 (m, 1H), 4.96 (t, *J* = 3.8 Hz, 1H), 4.09 (dt, *J* = 9.6, 3.9 Hz, 1H), 2.49 (d, *J* = 3.6 Hz, 1H), 1.70 (m, 2H), 1.01 (t, *J* = 7.3 Hz, 3H). ^{13}C-NMR (75.5 MHz, $CDCl_3$): δ 162.8 (d, *J* = 246.2 Hz, C), 142.3 (C), 129.9 (CH), 122.1 (CH), 114.9 (d, *J* = 21.3 Hz, CH), 113.6 (d, *J* = 22.4 Hz, CH), 76.3 (CH), 69.9 (CH), 24.6 (CH_2), 11.2 (CH_3). ^{19}F-RMN (282 MHz, $CDCl_3$): δ –112.63. HRMS (APCI-, m/z): calcd. for $C_{10}H_{11}{}^{35}ClF$ ($[M + Cl]^-$) 207.0255; found, 207.0272.

anti*-2-Chloro-1-(3-fluorophenyl)butan-1-ol** (anti*-3f**): Colorless oil (18 mg, 89% yield). R_f (Hex:EtOAc 10:1): 0.17. (1*S*,2*R*): $[\alpha]^{20}_{D}$ = -34.0 (0.1 *c*, $CHCl_3$). IR: ν 3380, 2970, 1591, 1137, 783 cm^{-1}. ^1H-NMR (300.13 MHz, $CDCl_3$): δ 7.33 (td, *J* = 8.0, 5.9 Hz, 1H), 7.16–6. 92 (m, 3H), 4.68 (d, *J* = 6.6 Hz, 1H), 4.01 (ddd, *J* = 9.0, 6.7, 4.2 Hz, 1H), 2.88 (s, 1H), 1.71–1.60 (m, 2H), 1.03 (t, *J* = 7.3 Hz, 3H). ^{13}C-NMR (75.5 MHz, $CDCl_3$): δ 163.0 (d, *J* = 246.4 Hz, C), 142.8 (d, *J* = 6.9 Hz, C), 130.2 (d, *J* = 8.1 Hz, CH), 122.6 (d, *J* = 2.9 Hz, CH), 115.4 (d, *J* = 21.3 Hz, CH), 113.9 (d, *J* = 22.1 Hz, CH), 76.7 (CH), 71.6 (CH), 27.7 (CH_2), 11.2 (CH_3). ^{19}F-RMN (282 MHz, $CDCl_3$): δ –112.46. HRMS (APCI+, m/z): calcd. for $C_{10}H_{11}{}^{35}ClF$ ($[M – OH]^+$) 185.0528; found, 185.0496.

syn*-2-Chloro-1-(2-fluorophenyl)butan-1-ol** (syn*-3g**): Yellow oil (17 mg, 85% yield). R_f (Hex:EtOAc 10:1): 0.15. (1*R*,2*R*): $[\alpha]^{20}_{D}$= +28.3 (0.1 *c*, $CHCl_3$). IR: ν 3380, 2973, 1515, 1184, 690 cm^{-1}. ^1H-NMR (300.13 MHz, $CDCl_3$): δ 7.69–7.45 (m, 1H), 7.36–7.27 (m, 1H), 7.18 (t, *J* = 7.5 Hz, 1H), 7.11–6.92 (m, 1H), 5.25 (d, *J* = 3.6 Hz, 1H), 4.21 (dt, *J* = 9.5, 3.5 Hz, 1H), 2.54 (s, 1H), 1.71 (tdd, *J* = 17.1, 8.4, 5.4 Hz, 2H), 1.01 (t, *J* = 7.3 Hz, 3H). ^{13}C-NMR (75.5 MHz, $CDCl_3$): δ 159.8 (d, *J* = 250.6 Hz, C), 129.5 (d, *J* = 8.3 Hz, CH), 128.4 (d, *J* = 3.8 Hz, CH), 126.9 (d, *J* = 13.1 Hz, C), 124.2 (d, *J* = 3.8 Hz, CH), 115.2 (d, *J* = 21.7 Hz, CH), 71.2 (CH), 68.9 (CH), 24.8 (CH_2), 11.3 (CH_3). ^{19}F-RMN (282 MHz, $CDCl_3$): δ –118.09. HRMS (APCI+, m/z): calcd. for $C_{10}H_{11}{}^{35}ClF$ ($[M – OH]^+$) 185.0528; found, 185.0524.

anti*-2-Chloro-1-(2-fluorophenyl)butan-1-ol** (anti*-3g**): Yellow oil (17 mg, 85% yield). R_f (Hex:EtOAc 10:1): 0.15. (1*S*,2*R*): $[\alpha]^{20}_{SR}$ = -118.3 (0.1 *c*, $CHCl_3$). IR: ν 3432, 2968, 1507, 1214, 697 cm^{-1}. ^1H-NMR (300.13 MHz, $CDCl_3$): δ 7.47 (t, *J* = 7.1

Hz, 1H), 7.36–7.26 (m, 1H), 7.19 (t, J = 7.4 Hz, 1H), 7.05 (t, J = 9.3 Hz, 1H), 5.07 (d, J = 5.7 Hz, 1H), 4.12 (q, J = 6.4 Hz, 1H), 2.75 (s, 1H), 1.77 (quint, J = 7.0 Hz, 2H), 1.06 (t, J = 7.2 Hz, 3H). ^{13}C-NMR (75.5 MHz, $CDCl_3$): δ 158.7 (C), 129.7 (d, J = 8.3 Hz, CH), 128.1 (d, J = 3.6 Hz, CH), 127.6 (d, J = 13.1 Hz, C), 124.3 (d, J = 2.8 Hz, CH), 115.4 (d, J = 22.0 Hz, CH), 71.2 (CH), 70.1 (CH), 27.7 (CH_2), 11.2 (CH_3). ^{19}F-RMN (282 MHz, $CDCl_3$): δ –118.33. HRMS (APCI+, m/z): calcd. for $C_{10}H_{11}{}^{35}ClF$ ($[M - OH]^+$) 185.0528; found, 185.0532.

V.5. Scale-up of the bienzymatic synthesis of chlorohydrin ***3a***

Table S18. Semipreparative bienzymatic reduction of ketone **1a** (50 mg).

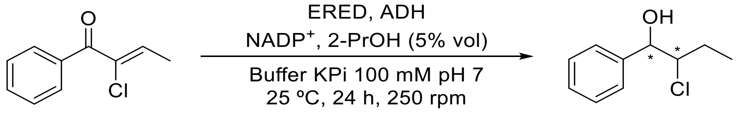

1a (50 mg, 25 mM) **3a**

Entry	ERED	ADH	**3a** *c* (%)[a]	*syn:anti*[b]	**3a** *ee* (%)[b]
1	110	evo.1.1.200	>99 (87)	1:>99	>99 (1*S*,2*R*)
2	110	*Lb*ADH	>99 (91)	>99:1	>99 (1*R*,2*R*)
3	P1-H09	*Lb*ADH	>99 (89)	>99:1	85 (1*S*,2*S*)

[a] Determined by GC. Isolated yields in parentheses. [b] Determined by HPLC. Configuration of the major enantiomer in parentheses.

Experimental procedure for the semipreparative bienzymatic reduction of ketone 1a (1 mmol scale) using commercial ERED-110 and evo.1.1.200: evo.1.1.200 (160 mg) was added to a 500 mL Erlenmeyer flask containing chloroenone **1a** (172 mg, 1 mmol), 2-PrOH (4 mL, 5% vol), a NADH 10 mM aqueous solution (1 mL), a NADPH 10 mM aqueous solution (1 mL), a $MgCl_2$ 10 mM aqueous solution (2 mL), KPi buffer 100 mM pH 7 (34 mL) and ERED-110 (80 mg). Then, the recipient was closed and kept under orbital shaking at 250 rpm at 25 °C for 24 h. After this time, the solution was extracted with EtOAc (3 x 15 mL), and the combined organic layers were dried over anhydrous Na_2SO_4, filtered and evaporated under reduced pressure. The corresponding chlorohydrin (1*S*,2*R*)-**3a** was recovered as a pale yellow oil (148 mg, 81% yield) in 1:99 *dr* (*syn*:*anti*) and 99% *ee*.

VI. Reference section

1. Lavandera, I.; Kern, A.; Ferreira-Silva, B.; Glieder, A.; de Wildeman, S.; Kroutil, W. Stereoselective bioreduction of bulky-bulky ketones by a novel ADH from *Ralstonia* sp. *J. Org. Chem*. **2008**, *73,* 6003-6005.

2. Lavandera, I.; Kern, A.; Resch, V.; Ferreira-Silva, B.; Glieder, A.; Fabian, W. M. F.; de Wildeman, S.; Kroutil, W. One-way biohydrogen transfer for oxidation of *sec*-alcohols. *Org. Lett.* **2008**, *10*, 2155-2158.

3. Findrik, Z.; Vasić-Rački, D.; Lütz, S.; Daussmann, T.; Wandrey, C. Kinetic modeling of acetophenone reduction catalyzed by alcohol dehydrogenase from *Thermoanaerobacter* sp. *Biotechnol. Lett.* **2005**, *27,* 1087-1095.

4. Wolberg, M.; Hummel, W.; Wandrey, C.; Müller, M. Highly regio- and enantioselective reduction of 3,5-dioxocarboxylates. *Angew. Chem. Int. Ed.* **2000**, *39*, 4306-4308.

5. Heiss, C.; Laivenieks, M.; Zeikus, J. G.; Phillips, R. S. Mutation of cysteine-295 to alanine in secondary alcohol dehydrogenase from *Thermoanaerobacter ethanolicus* affects the enantioselectivity and substrate specificity of ketone reductions. *Bioorg. Med. Chem.* **2001**, *9*, 1659-1666.

6. Edegger, K.; Gruber, C. C.; Poessl, T. M.; Wallner, S. R.; Lavandera, I.; Faber, K.; Niehaus, F.; Eck, J.; Oehrlein, R.; Hafner, A.; Kroutil, W. Biocatalytic deuterium- and hydrogen-transfer using over-expressed ADH-'A': Enhanced stereoselectivity and ^{2}H-labeled chiral alcohols. *Chem. Commun.* **2006**, 2402-2404.

7. Grunwald, J.; Wirz, B.; Scollar, M. P.; Klibanov, A. M. Asymmetric oxidoreductions catalyzed by alcohol dehydrogenase in organic solvents. *J. Am. Chem. Soc.* **1986**, *108*, 6732-6734.

8. Araki, S.; Hirashita, T.; Shimizu, H.; Yamamura, H.; Kawai, M.; Butsugan, Y. Indium-mediated reaction of 1,3-dichloro and 1,3-dibromopropene with carbonyl compounds. Generation of novel 3,3-diindiopropene. *Tetrahedron Lett*. **1996**, *37,* 8417-8420.

9. Bandini, M.; Cozzi, P. G.; Melchiorre, P.; Morganti, S.; Ronchi A. U. Cr(Salen)-catalyzed addition of 1,3-dichloropropene to aromatic aldehydes. A simple access to optically active vinyl epoxides. *Org. Lett*. **2001**, *3*, 1153-1155.

10. Masuyama, Y.; Ito, A.; Kurusu, Y. Either γ-*syn* or γ-*anti*-selective palladium-catalyzed carbonyl allylation by mixed (*E*)- and (*Z*)-1,3-dichloropropene with tin(II) halides. *Chem. Commun*. **1998**, *3*, 315-316.

11. Sadhukan, S.; Baire, B. Formal halo Meyer-Schuster rearrangement of propargylic acetates through a novel intermediate and an unexampled mechanistic pathway. *Chem. Eur. J.* **2019**, *25*, 9816-9820.

12. Lo Fiego, M. J.; Badajoz, M. A.; Domini, C.; Chopa, A. B.; Lockhart, M. T. Indium-mediated regioselective synthesis of ketones from arylstannanes under solvent-free ultrasound irradiation. *Ultrason. Sonochem.* **2013**, *20*, 826-832.

13. Guha, S.; Kazi, I.; Mukherjee, P.; Sekar, G. Halogen-bonded iodonium ion catalysis: A route to α-hydroxy ketone via domino oxidations of secondary alcohol and aliphatic C-H bond with high selectivity and control. *Chem. Commun.* **2017**, *53*, 10942-10945.

14. Lauriers, A. J. D.; Legault, C. Y. Iodine (III)-mediated oxidative hydrolysis of haloalkenes: Access to α-halo ketones by a release-and-catch mechanism. *Org. Lett.* **2016**, *18*, 108-111.

15. Shimagaki, M.; Matsuzaki, Y.; Hori, I.; Nakata, T.; Oishi, T. Highly stereoselective synthesis of β-oxosulfonium salts, synthesis of *trans* epoxides. *Tetrahedron Lett.* **1984**, *25*, 4779-4782.

VII. Analytical data

VII.1. GC for the determination of the conversions

An Agilent HP-1 (30 m x 0.32 mm x 0.25 μm) column was used for the determination of the conversion values in the bienzymatic cascade protocol.

Table S19. GC analytical conditions and retention times for the determination of conversion values in HP-1 column.[a]

Entry	Substrate	Retention time (min)
1	**1a**	11.5
2	**2a**	10.0
3	*syn*-**3a**	11.1
4	*anti*-**3a**	10.7
5	**1b**	14.6
6	**2b**	13.3
7	*syn*-**3b**	13.0
8	*anti*-**3b**	13.8
9	**1c**	15.6
10	**2c**	14.2
11	*syn*-**3c**	15.9
12	*anti*-**3c**	15.2
13	**1d**	19.0
14	**2d**	17.0
15	*syn*-**3d**	18.8
16	*anti*-**3d**	18.4
17	**1e**	10.9
18	**2e**	9.4
19	*syn*-**3e**	10.6
20	*anti*-**3e**	10.2
21	**1f**	10.5
22	**2f**	9.2
23	*syn*-**3f**	11.0
24	*anti*-**3f**	10.0
25	**1g**	11.1
26	**2g**	9.0
27	*syn*-**3g**	10.5
28	*anti*-**3g**	9.7
29	**10a**	12.6

[a] GC program: initial temp. (80 ºC) / time (1 min) / ramp (10 ºC/min) / temp. (120 ºC) / time (5 min) / ramp (10 ºC/min) / temp. (150 ºC) / time (5 min) / ramp (10 ºC/min) / final temp. (200 ºC) / time (1 min). 12.2 psi N_2.

VII.2. HPLC analyses for the determination of enantiomeric excess

Table S20. HPLC analytical conditions and retention times for the determination of the enantiomeric excess of ketones **2a-g**.

Entry	Ketone	Column	Eluent[a]	Flow (mL/min)	(*R*)-**2** (min)	(*S*)-**2** (min)
1[b]	**2a**	Chiralpak OD-H	0.5:99.5	1.0	7.3	8.1
2		Chiralpak AD-H	5:95	0.5	9.5	10.1
3	**2b**	Chiralpak AD-H	10:90	0.7	8.1	8.5
4	**2c**	Chiralpak AD-H	5:95	0.5	9.8	10.5
5	**2d**	Chiralpak AD-H	5:95	0.5	10.2	11.2
6	**2e**	Chiralpak AD-H	5:95	0.5	9.5	10.0
7	**2f**	Chiralpak AD-H	5:95	0.5	8.9	9.3
8	**2g**	Chiralpak AD-H	2:98	0.5	8.4	8.7

[a] The eluent was composed by mixtures of 2-propanol/ *n*-hexane and the column displays ratio in volume.
[b] Determined by using the method reported by Lauriers and Legault.[14]

HPLC separation for both enantiomers of racemic ketone 2a in OD-H column

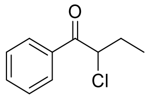

2a

HPLC (OD-H, 2-propanol/*n*-hexane = 0.5/99.5, flow rate = 1.0 mL/min, l = 210 nm)

Ketone (*R*)-**2a** in 99% *ee* (after bioreduction with ERED-110)

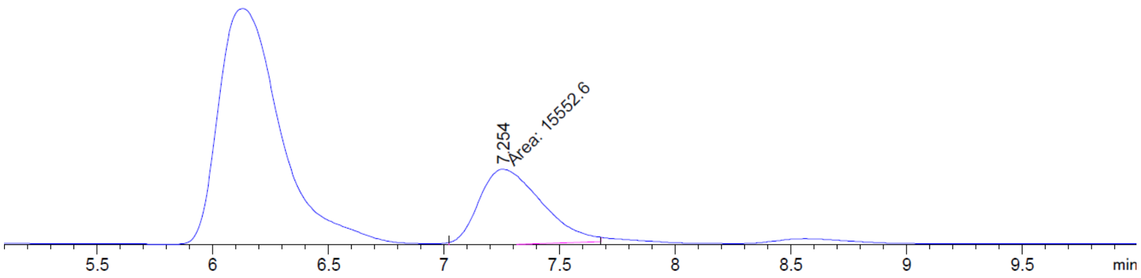

Peak #	RetTime [min]	Type	Width [min]	Area [mAU*s]	Height [mAU]	Area %
1	7.254	MM	0.3164	1.55526e4	819.23669	100.0000

Ketone (*S*)-**2a** in 99% *ee* (after bioreduction with ERED P1-H09) (8.5 min peak stands for starting material)

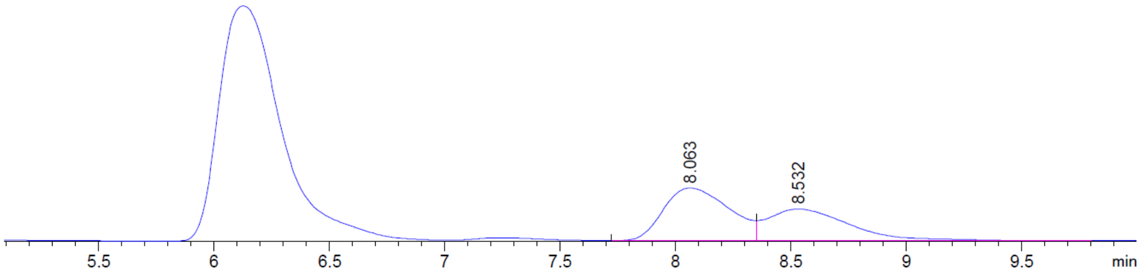

Peak #	RetTime [min]	Type	Width [min]	Area [mAU*s]	Height [mAU]	Area %
1	8.063	BV	0.3137	1.13886e4	573.50775	57.2730
2	8.532	VB	0.3771	8496.16602	345.30054	42.7270

Figure S2. HPLC chromatograms of optically active **2a**. Peak observed at 6.2 min correspond to the solvent (EtOAc), while the one at 8.5 min corresponds to the starting material **1a.**

HPLC separation for both enantiomers of racemic ketone 2a in AD-H column

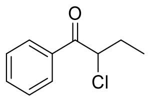

2a

HPLC (AD-H, 2-propanol/*n*-hexane = 10/90, flow rate = 0.7 mL/min, l = 210 nm)

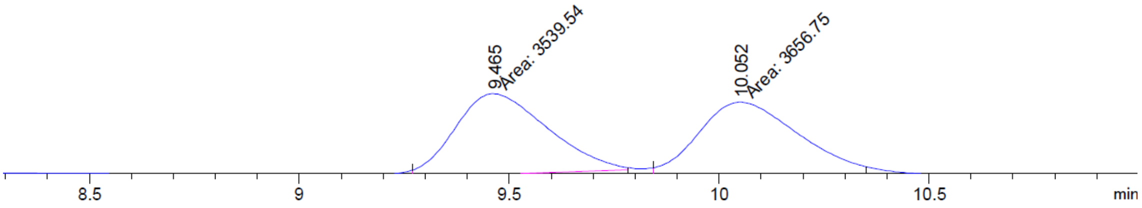

Peak #	RetTime [min]	Type	Width [min]	Area [mAU*s]	Height [mAU]	Area %
1	9.465	MM	0.2566	3539.53882	229.91002	49.1856
2	10.052	MM	0.2846	3656.75317	214.15321	50.8144

Ketone (*R*)-**2a** in 99% *ee* (after bioreduction with ERED-110)

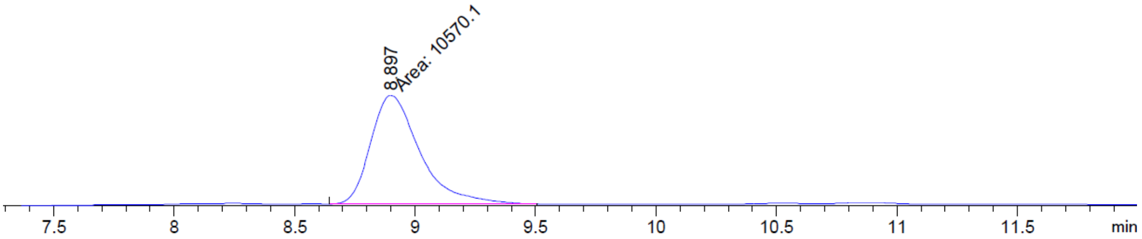

Peak #	RetTime [min]	Type	Width [min]	Area [mAU*s]	Height [mAU]	Area %
1	8.897	MM	0.2344	1.05701e4	751.66162	100.0000

Ketone (*S*)-**2a** in 99% *ee* (after bioreduction with ERED P1-H09)

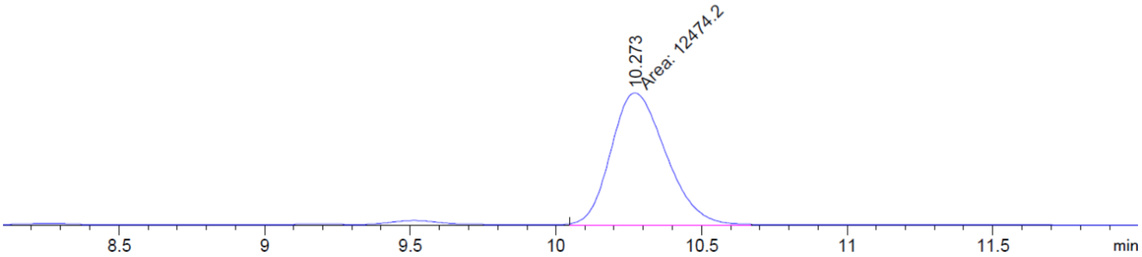

Peak #	RetTime [min]	Type	Width [min]	Area [mAU*s]	Height [mAU]	Area %
1	10.273	MM	0.2193	1.24742e4	947.83875	100.0000

Figure S3. HPLC chromatograms of optically active **2a** obtained using selective EREDs separated in Chiralpak AD-H column.

HPLC separation for both enantiomers of racemic ketone 2b

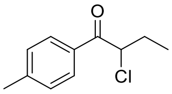

2b

HPLC (AD-H, 2-propanol/*n*-hexane = 5/95, flow rate = 0.5 mL/min, l = 210 nm)

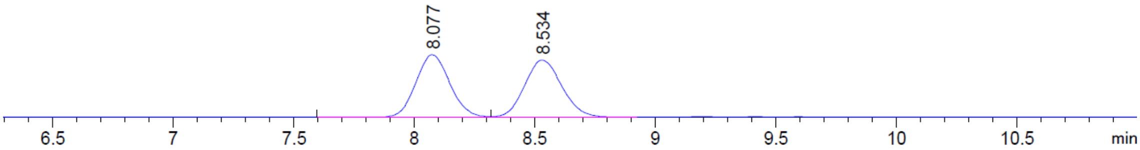

Peak #	RetTime [min]	Type	Width [min]	Area [mAU*s]	Height [mAU]	Area %
1	8.077	BV	0.1521	1686.21509	172.35324	49.9499
2	8.534	VB	0.1671	1689.59949	157.55386	50.0501

Ketone (*R*)-**2b** in >99% *ee* (after bioreduction with ERED-110)

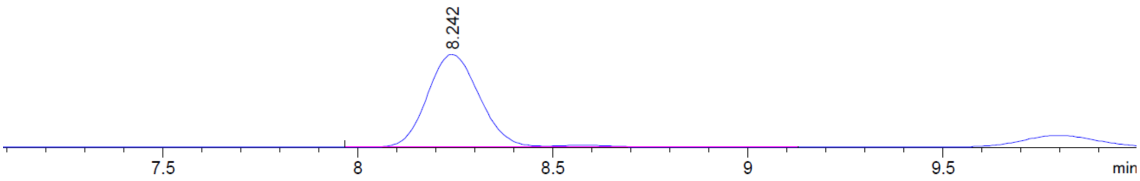

Peak #	RetTime [min]	Type	Width [min]	Area [mAU*s]	Height [mAU]	Area %
1	8.242	BV R	0.1437	1.55586e4	1653.27673	100.0000

Ketone (*S*)-**2b** in 94% *ee* (after bioreduction with ERED P1-H09)

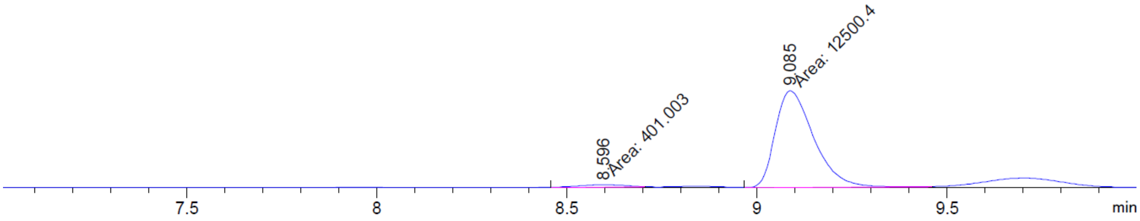

Peak #	RetTime [min]	Type	Width [min]	Area [mAU*s]	Height [mAU]	Area %
1	8.596	MM	0.1410	401.00262	47.39416	3.1082
2	9.085	MM	0.1171	1.25004e4	1779.28577	96.8918

Figure S4. HPLC chromatograms of optically active **2b** obtained using selective EREDs separated in Chiralpak AD-H column. Peak observed at 9.7 min corresponds to the starting material **1b**.

HPLC separation for both enantiomers of racemic ketone 2c in AD-H column

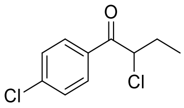

2c

HPLC (AD-H, 2-propanol/*n*-hexane = 5/95, flow rate = 0.5 mL/min, l = 210 nm)

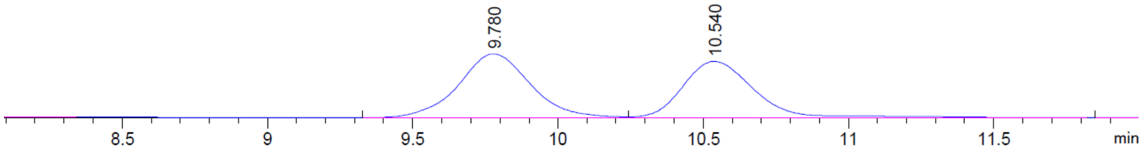

Peak #	RetTime [min]	Type	Width [min]	Area [mAU*s]	Height [mAU]	Area %
1	9.780	BV	0.2601	3906.15820	227.69518	53.0786
2	10.540	VB	0.2644	3453.03662	201.00868	46.9214

Ketone (*R*)-**2c** in 98% *ee* (after bioreduction with ERED-110)

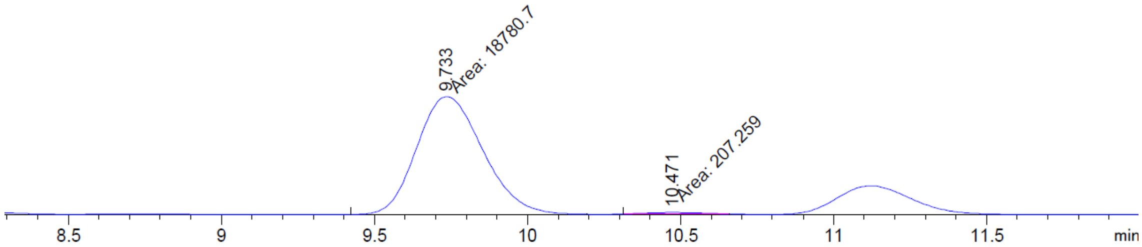

Peak #	RetTime [min]	Type	Width [min]	Area [mAU*s]	Height [mAU]	Area %
1	9.733	MM	0.2525	1.87807e4	1239.65381	98.9085
2	10.471	MM	0.2122	207.25926	16.27733	1.0915

Ketone (*S*)-**2c** in 85% *ee* (after bioreduction with ERED P1-H09)

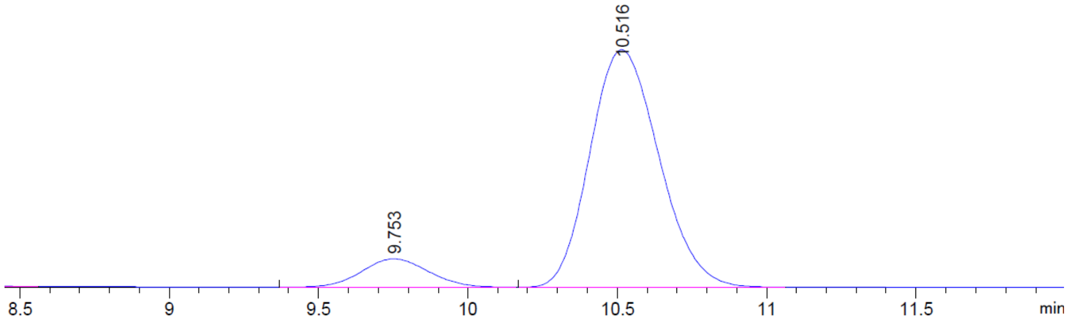

Peak #	RetTime [min]	Type	Width [min]	Area [mAU*s]	Height [mAU]	Area %
1	9.753	BB	0.2458	940.30988	59.67268	7.8533
2	10.516	BB	0.2513	8063.23730	502.29343	92.1467

Figure S5. HPLC chromatograms of optically active **2c** obtained using selective EREDs separated in Chiralpak AD-H column. Peak observed at 11.2 min corresponds to the starting material **1c**.

HPLC separation for both enantiomers of racemic ketone 2d in AD-H column

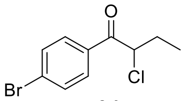

2d

HPLC (AD-H, 2-propanol/*n*-hexane = 5/95, flow rate = 0.5 mL/min, l = 210 nm)

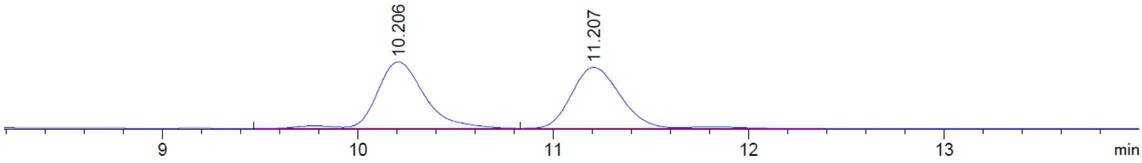

Peak #	RetTime [min]	Type	Width [min]	Area [mAU*s]	Height [mAU]	Area %
1	10.206	VB R	0.2542	3210.87769	189.16599	51.0738
2	11.207	BV R	0.2685	3075.86890	173.30736	48.9262

Ketone (*R*)-**2d** in >99% *ee* (after bioreduction with ERED-110)

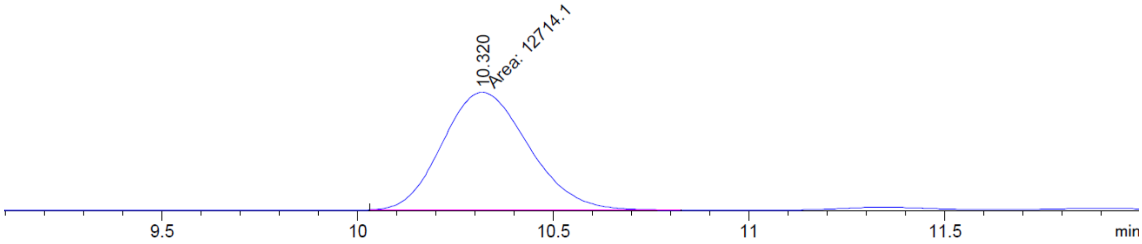

Peak #	RetTime [min]	Type	Width [min]	Area [mAU*s]	Height [mAU]	Area %
1	10.320	MM	0.2536	1.27141e4	835.60779	100.0000

Ketone (*S*)-**2d** in 86% *ee* (after bioreduction with ERED P1-H09)

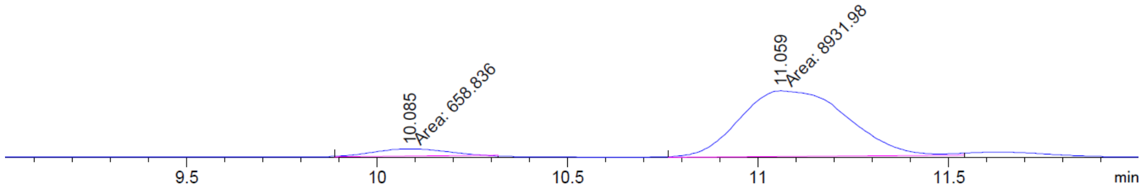

Peak #	RetTime [min]	Type	Width [min]	Area [mAU*s]	Height [mAU]	Area %
1	10.085	MM	0.2176	658.83582	50.45655	6.8694
2	11.059	MM	0.3264	8931.98145	456.02655	93.1306

Figure S6. HPLC chromatograms of optically active **2d** obtained using selective EREDs separated in Chiralpak AD-H column.

HPLC separation for both enantiomers of racemic ketone 2e in AD-H column

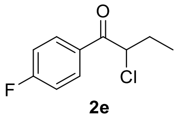

2e

HPLC (AD-H, 2-propanol/*n*-hexane = 5/95, flow rate = 0.5 mL/min, l = 210 nm)

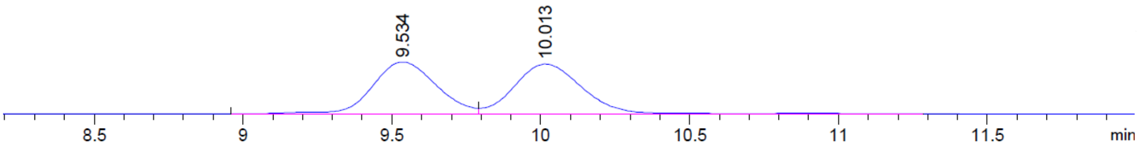

Peak #	RetTime [min]	Type	Width [min]	Area [mAU*s]	Height [mAU]	Area %
1	9.534	BV	0.2322	5429.64600	363.73236	49.3171
2	10.013	VV R	0.2434	5580.00830	350.10641	50.6829

Ketone (*R*)-**2e** in 96% *ee* (after bioreduction with ERED-110)

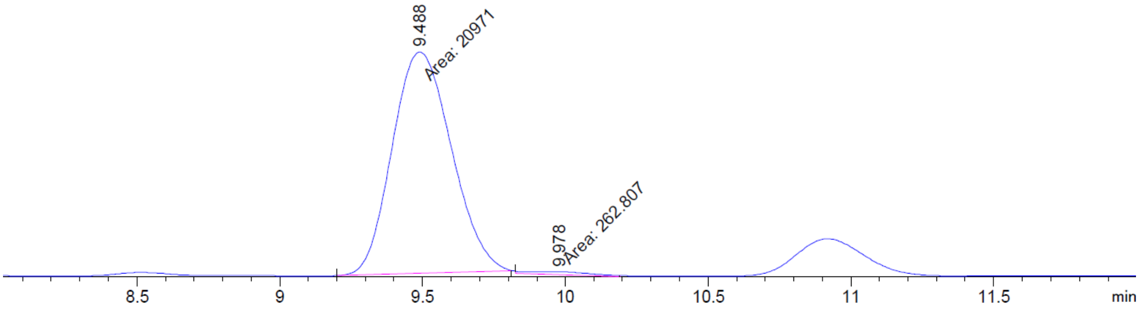

Peak #	RetTime [min]	Type	Width [min]	Area [mAU*s]	Height [mAU]	Area %
1	9.488	MM	0.2403	2.09710e4	1454.61194	98.7623
2	9.978	MM	0.2384	262.80685	18.37243	1.2377

Ketone (*S*)-**2e** in 86% *ee* (after bioreduction with ERED P1-H09)

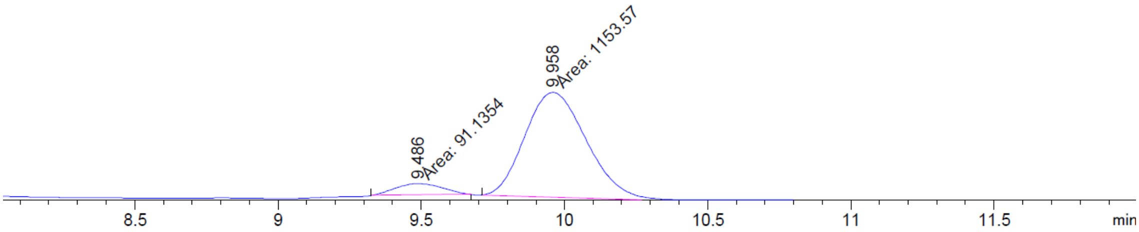

Peak #	RetTime [min]	Type	Width [min]	Area [mAU*s]	Height [mAU]	Area %
1	9.486	MM	0.1881	91.13535	8.07507	7.3218
2	9.958	MM	0.2511	1153.57178	76.57680	92.6782

Figure S7. HPLC chromatograms of optically active **2e** obtained using selective EREDs separated in Chiralpak AD-H column.

HPLC separation for both enantiomers of racemic ketone 2f in AD-H column

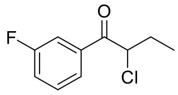

2f

HPLC (AD-H, 2-propanol/*n*-hexane = 5/95, flow rate = 0.5 mL/min, l = 210 nm)

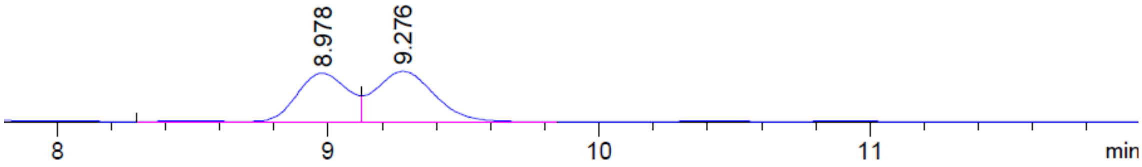

Peak #	RetTime [min]	Type	Width [min]	Area [mAU*s]	Height [mAU]	Area %
1	8.978	VV R	0.2023	2441.83691	185.92908	45.5652
2	9.276	VB	0.2296	2917.15576	193.80748	54.4348

Ketone (*R*)-**2f** in >99% *ee* (after bioreduction with ERED-110)

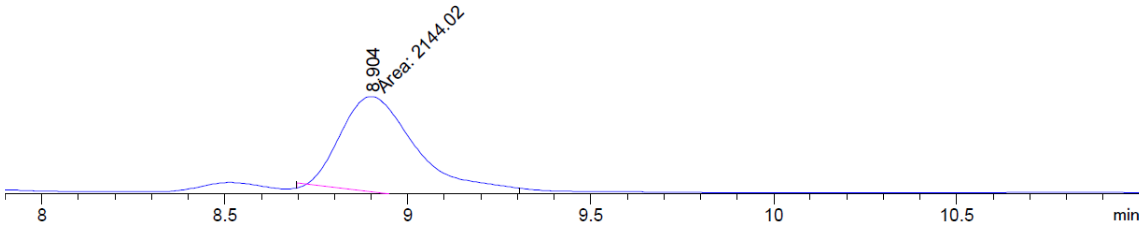

Peak #	RetTime [min]	Type	Width [min]	Area [mAU*s]	Height [mAU]	Area %
1	8.904	MM	0.2715	2144.02002	131.63684	100.0000

Ketone (*S*)-**2f** in 90% *ee* (after bioreduction with ERED P1-H09)

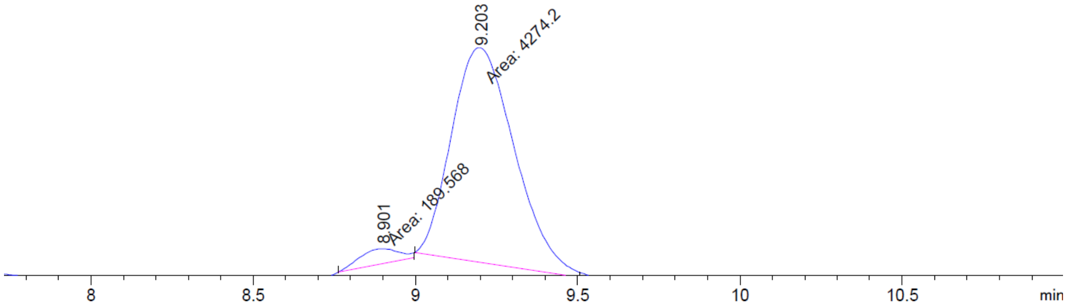

Peak #	RetTime [min]	Type	Width [min]	Area [mAU*s]	Height [mAU]	Area %
1	8.901	MM	0.1437	189.56763	21.98267	4.2468
2	9.203	MM	0.2251	4274.19775	316.51730	95.7532

Figure S8. HPLC chromatograms of optically active **2f** obtained using selective EREDs separated in Chiralpak AD-H column.

HPLC separation for both enantiomers of racemic ketone 2g in AD-H column

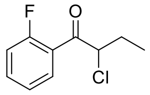

2g

HPLC (AD-H, 2-propanol/*n*-hexane = 2/98, flow rate = 0.5 mL/min, l = 210 nm)

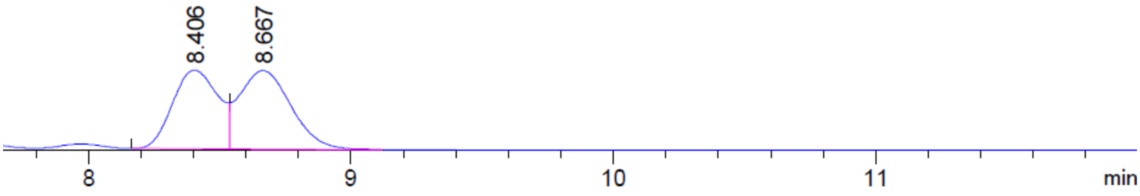

Peak #	RetTime [min]	Type	Width [min]	Area [mAU*s]	Height [mAU]	Area %
1	8.406	BV	0.1850	3726.34326	312.79669	46.4122
2	8.667	VB	0.2089	4302.45313	311.97614	53.5878

Ketone (*R*)-**2g** in 62% *ee* (after bioreduction with ERED-110)

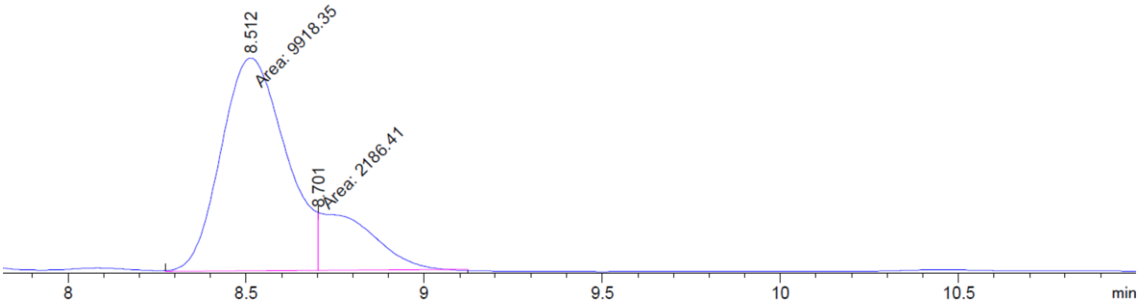

Peak #	RetTime [min]	Type	Width [min]	Area [mAU*s]	Height [mAU]	Area %
1	8.512	MF	0.2116	9918.34570	781.03839	81.9376
2	8.701	FM	0.1724	2186.40674	211.41441	18.0624

Ketone (*S*)-**2g** in 44% *ee* (after bioreduction with ERED P1-H09)

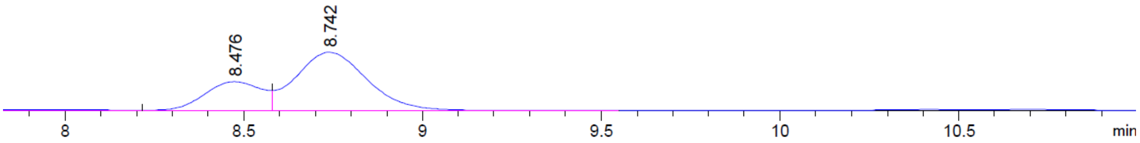

Peak #	RetTime [min]	Type	Width [min]	Area [mAU*s]	Height [mAU]	Area %
1	8.476	VV	0.1704	3450.79639	313.64087	28.1241
2	8.742	VB	0.2119	8819.09766	635.63165	71.8759

Figure S9. HPLC chromatograms of optically active **2g** obtained using selective EREDs separated in Chiralpak AD-H column.

Table S21. HPLC analytical conditions and retention times for the determination of enantiomeric excess values of halohydrins **3a-g**.

Entry	Chlorohydrin	Column[a]	Eluent[a]	Flow (mL/min)	(1*R*,2*R*)-**3** (min)	(1*S*,2*S*)-**3** (min)	(1*S*,2*R*)-**3** (min)	(1*R*,2*S*)-**3** (min)
1	**3a**	Chiralcel OJ-H	5:95	0.5	14.1	15.1	29.2	30.7
2		Chiralpak AD-H	5:95	0.5	n.d.	n.d.	23.7	20.9
3		Chiralpak IC	3:97	0.3	26.7	23.8	n.d.	n.d.
4	**3b**	Chiralcel OJ-H	10:90	0.7	10.4	10.7	11.1	12.4
5	**3c**	Chiralpak AD-H	5:95	0.5	17.8	18.4	21.4	20.0
6		Chiralpak IC	3:97	0.3	24.1	23.7	n.d.	n.d.
7	**3d**	Chiralpak AD-H	5:95	0.5	18.6	19.5	22.9	21.3
8	**3e**	Chiralpak AD-H	5:95	0.5	n.d.	n.d.	17.8	18.5
9		Chiralpak IC	3:97	0.3	24.8	24.0	n.d.	n.d.
10	**3f**	Chiralpak AD-H	5:95	0.5	15.6	16.7	20.3	18.3
11	**3g**	Chiralpak AD-H	2:98	0.5	15.6	15.6	18.7	17.7
12		Chiralpak IC	3:97	0.3	23.5	22.4	n.d.	n.d.

[a] The eluent was composed by mixtures of 2-propanol/ *n*-hexane and the column displays ratio in volume. n.d.: Not determined.

HPLC separation in the OJ-H column of all diastereoisomers of chlorohydrin 3a obtained via chemical reduction of the corresponding chloroketone 2a

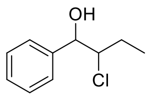

3a

HPLC (OJ-H, 2-propanol/*n*-hexane = 5/95, flow rate = 0.5 mL/min, l = 210 nm)

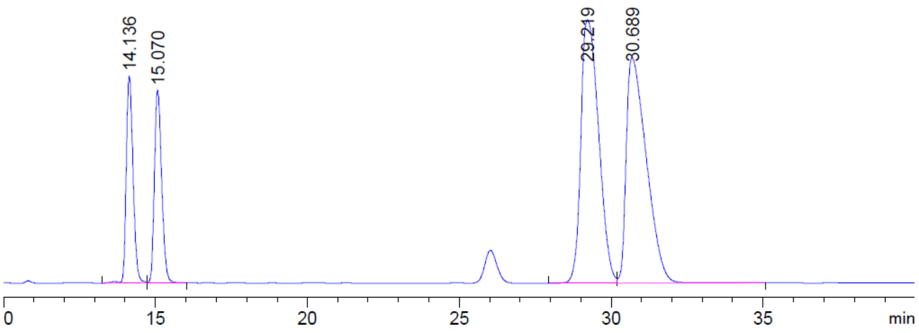

Peak #	RetTime [min]	Type	Width [min]	Area [mAU*s]	Height [mAU]	Area %
1	14.136	VV R	0.2419	3.14941e4	2027.11743	11.6819
2	15.070	VB	0.2570	3.12571e4	1889.23108	11.5940
3	29.219	BV	0.6408	1.04897e5	2577.59741	38.9089
4	30.689	VB	0.7047	1.01949e5	2207.89697	37.8153

(1*R*,2*R*)-**3a** in >99% *ee* and >99:1 *dr* (bioreduction with ERED-110 and *Lb*ADH)

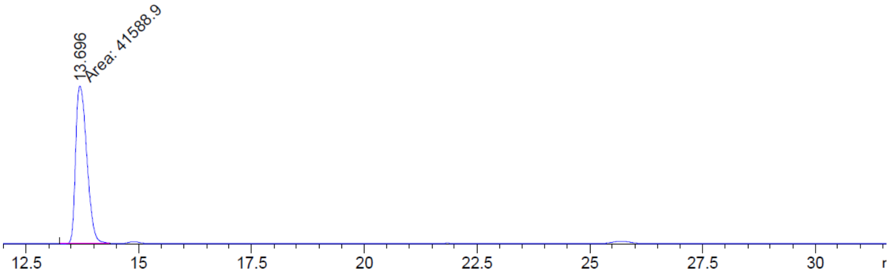

Peak #	RetTime [min]	Type	Width [min]	Area [mAU*s]	Height [mAU]	Area %
1	13.696	MM	0.2856	4.15889e4	2427.02783	100.0000

(1*S*,2*S*)-**3a** in 85% *ee* and >99:1 *dr* (bioreduction with ERED P1-H09 and *Lb*ADH)

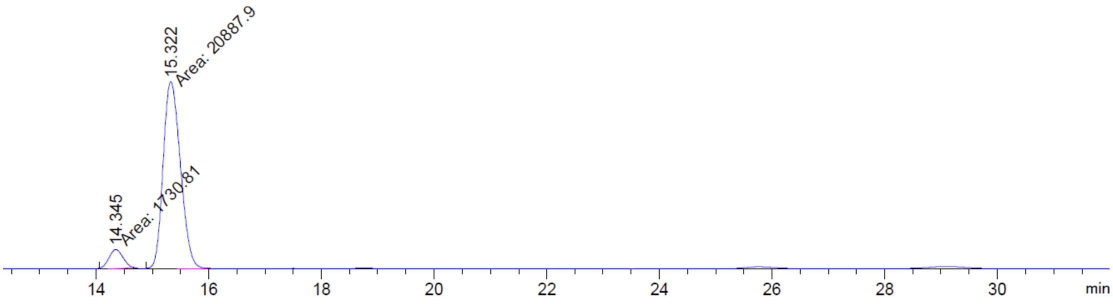

Peak #	RetTime [min]	Type	Width [min]	Area [mAU*s]	Height [mAU]	Area %
1	14.345	MM	0.2903	1730.81116	99.36265	7.6521
2	15.322	MM	0.3567	2.08879e4	975.99982	92.3479

(1*S*,2*R*)-**3a** in >99% *ee* and >1:99 *dr* (bioreduction with ERED-110 and evo.1.1.200)

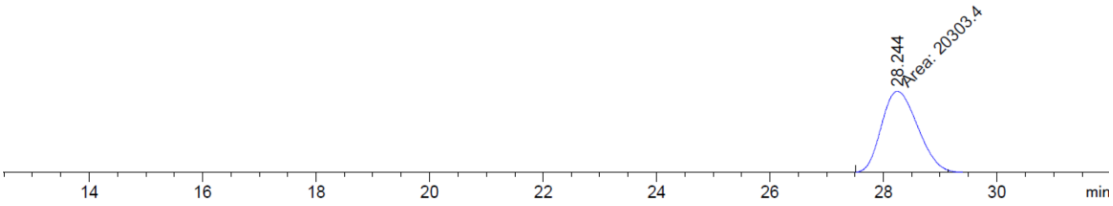

Peak #	RetTime [min]	Type	Width [min]	Area [mAU*s]	Height [mAU]	Area %
1	28.244	MM	0.7737	2.03034e4	437.37015	100.0000

Figure S10. HPLC chromatograms of racemic and optically active **3a** in Chiralcel OJ-H column.

HPLC separation in the AD-H column of the *anti*-enantiomers of chlorohydrin 3a obtained via chemical reduction of the corresponding chloroketone 2a

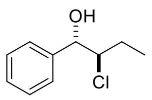

anti-**3a**

HPLC (AD-H, 2-propanol/*n*-hexane = 5/95, flow rate = 0.5 mL/min, l = 210 nm)

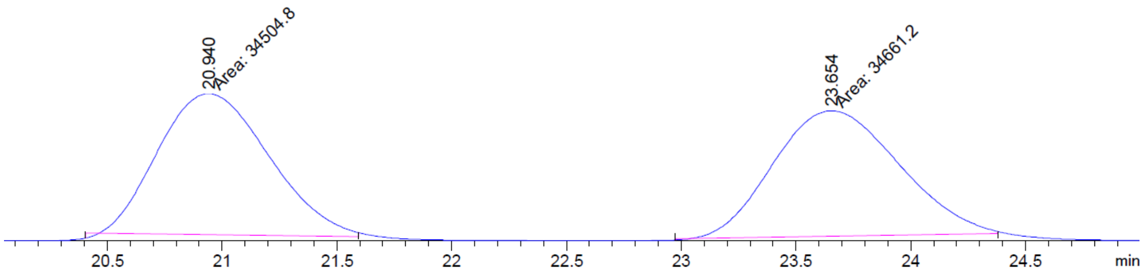

Peak #	RetTime [min]	Type	Width [min]	Area [mAU*s]	Height [mAU]	Area %
1	20.940	MM	0.5588	3.45048e4	1029.21558	49.8869
2	23.654	MM	0.6324	3.46612e4	913.46674	50.1131

Chlorohydrin (1*S*,2*R*)-**3a** in >99% *ee* and >1:99 *dr* (bioreduction with ERED-110 and evo.1.1.200)

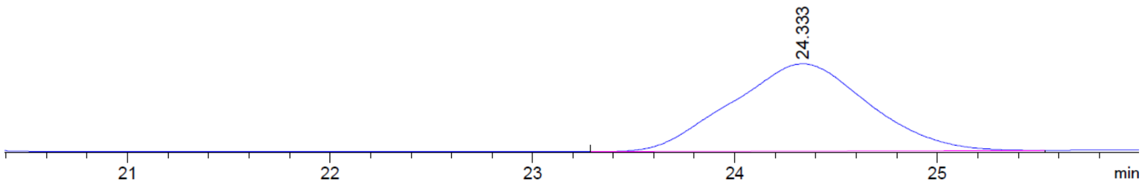

Peak #	RetTime [min]	Type	Width [min]	Area [mAU*s]	Height [mAU]	Area %
1	24.333	BB	0.6842	2.92459e4	622.38629	100.0000

Figure S11. HPLC chromatograms of racemic and optically active *anti*-**3a** in Chiralpak AD-H column.

HPLC separation in the IC column of the *syn*-enantiomers of racemic chlorohydrin 3a

HPLC (IC, 2-propanol/*n*-hexane = 3/97, flow rate = 0.3 mL/min, l = 210 nm)

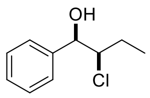

syn-**3a**

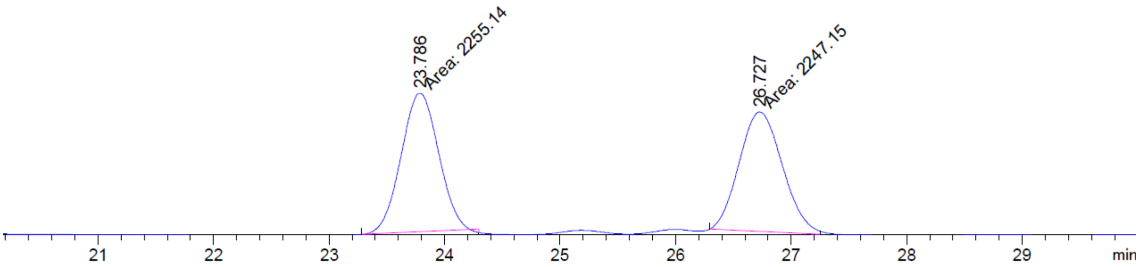

Peak #	RetTime [min]	Type	Width [min]	Area [mAU*s]	Height [mAU]	Area %
1	23.786	MM	0.3741	2255.14136	100.45841	50.0888
2	26.727	MM	0.4327	2247.14868	86.54883	49.9112

Chlorohydrin (1*R*,2*R*)-**3a** in >99% *ee* and >99:1 *dr* (bioreduction with ERED-110 and *Lb*ADH)

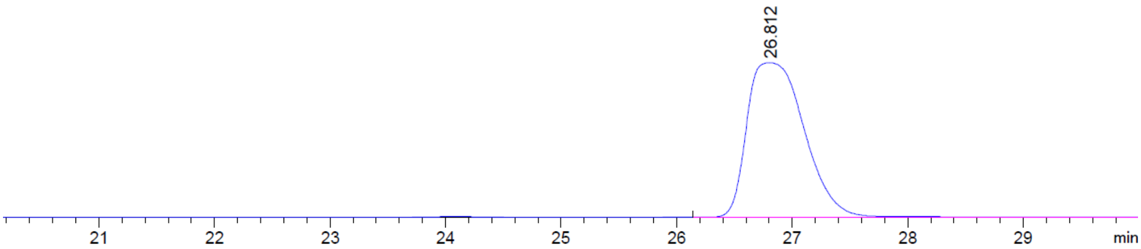

Peak #	RetTime [min]	Type	Width [min]	Area [mAU*s]	Height [mAU]	Area %
1	26.812	BB	0.4852	9.64873e4	2791.60034	100.0000

Figure S12. HPLC chromatograms of racemic and optically active *syn*-**3a** in Chiralpak IC column.

HPLC separation in the OJ-H column of all diastereoisomers of chlorohydrin 3b obtained via chemical reduction of the corresponding chloroketone 2b

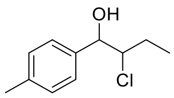

3b

HPLC (OJ-H, 2-propanol/*n*-hexane = 10/90, flow rate = 0.7 mL/min, l = 210 nm)

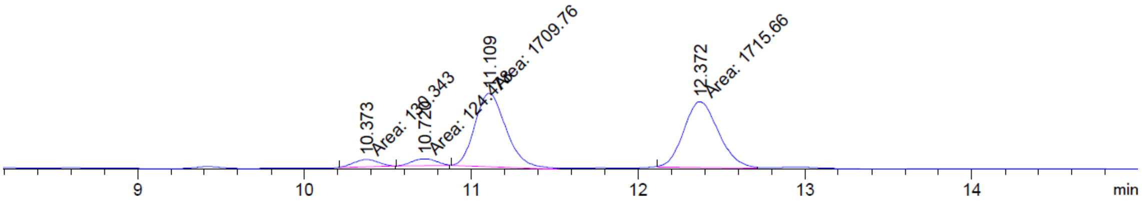

Peak #	RetTime [min]	Type	Width [min]	Area [mAU*s]	Height [mAU]	Area %
1	10.373	MM	0.1700	130.34271	12.78032	3.5417
2	10.720	MM	0.1687	124.47828	12.29564	3.3823
3	11.109	MM	0.2175	1709.75781	130.99741	46.4579
4	12.372	MM	0.2424	1715.65515	117.97478	46.6181

Chlorohydrin (1*R*,2*R*)-**3b** in >99% *ee* and 99:1 *dr* (bioreduction with ERED-110 and evo.1.1.200)

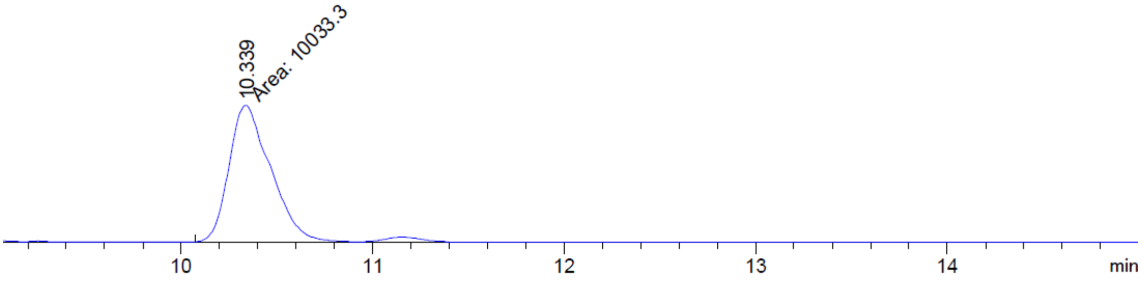

Peak #	RetTime [min]	Type	Width [min]	Area [mAU*s]	Height [mAU]	Area %
1	10.339	MM	0.2704	1.00333e4	618.49774	100.0000

Chlorohydrin (1*R*,2*S*)-**3b** in >99% *ee* and >1:99 *dr* (bioreduction with ERED P1-H09 and *Ras*ADH)

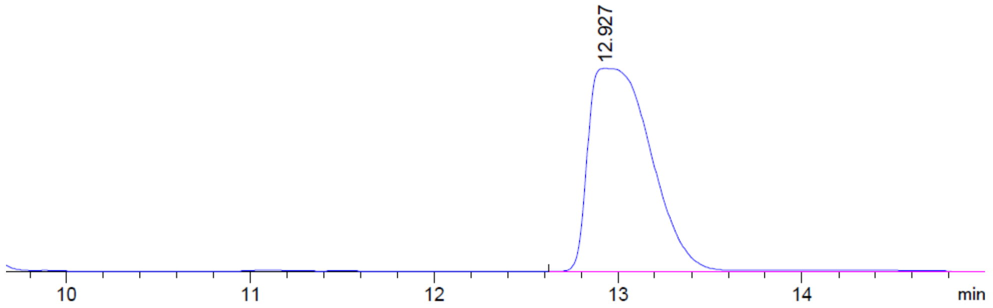

Peak #	RetTime [min]	Type	Width [min]	Area [mAU*s]	Height [mAU]	Area %
1	12.927	BV R	0.3082	6.30222e4	2640.71680	100.0000

Figure S13. HPLC chromatograms of racemic and optically active **3b** in Chiralcel OJ-H column.

HPLC separation in AD-H column of all diastereoisomers of chlorohydrin 3c obtained via chemical reduction of the corresponding chloroketone 2c

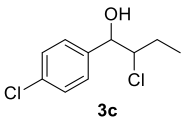

3c

HPLC (AD-H, 2-propanol/*n*-hexane = 5/95, flow rate = 0.5 mL/min, l = 210 nm)

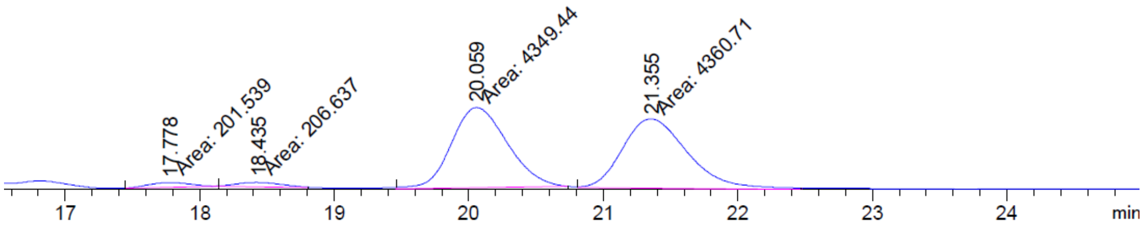

Peak #	RetTime [min]	Type	Width [min]	Area [mAU*s]	Height [mAU]	Area %
1	17.778	MM	0.3678	201.53856	9.13327	2.2103
2	18.435	MM	0.3956	206.63737	8.70599	2.2662
3	20.059	MM	0.4841	4349.43604	149.75244	47.7000
4	21.355	MM	0.5607	4360.70947	129.61617	47.8236

Chlorohydrin (1*R*,2*R*)-**3c** in >99% *ee* and 98:2 *dr* (bioreduction with ERED-110 and evo.1.1.200)

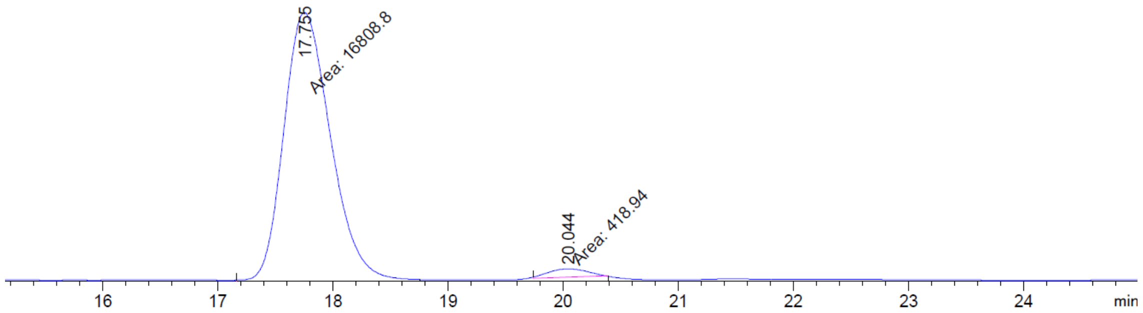

Peak #	RetTime [min]	Type	Width [min]	Area [mAU*s]	Height [mAU]	Area %
1	17.755	MM	0.4764	1.68088e4	588.09985	97.5682
2	20.044	MM	0.3887	418.94009	17.96307	2.4318

Chlorohydrin (1*S*,2*R*)-**3c** in >99% *ee* and 10:90 *dr* (bioreduction with ERED P1-H09 and *Ras*ADH)

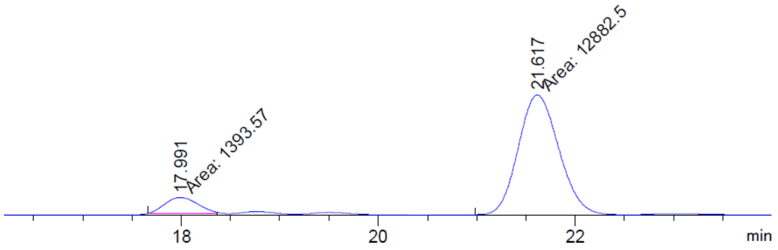

Peak #	RetTime [min]	Type	Width [min]	Area [mAU*s]	Height [mAU]	Area %
1	17.991	MM	0.3953	1393.56873	58.75168	9.7616
2	21.617	MM	0.4977	1.28825e4	431.38864	90.2384

Figure S14. HPLC chromatograms of racemic and optically active **3c** in Chiralpak AD-H column.

HPLC separation in the AD-H column of all diastereoisomers of chlorohydrin 3d obtained via chemical reduction of the corresponding chloroketone 2d

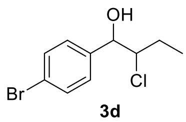

3d

HPLC (AD-H, 2-propanol/*n*-hexane = 5/95, flow rate = 0.5 mL/min, l = 210 nm)

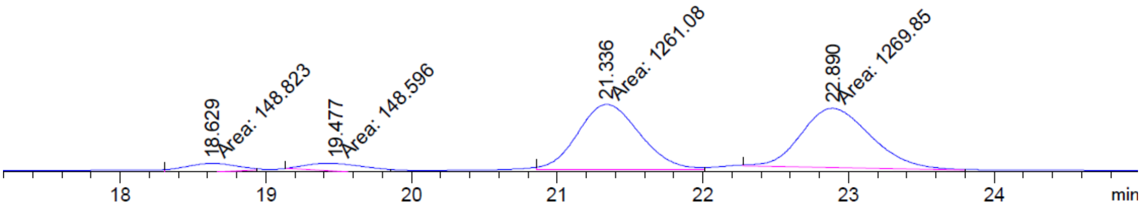

Peak #	RetTime [min]	Type	Width [min]	Area [mAU*s]	Height [mAU]	Area %
1	18.629	MM	0.4457	148.82283	5.56492	5.2618
2	19.477	MM	0.5202	148.59576	4.76089	5.2538
3	21.336	MM	0.5002	1261.08118	42.02125	44.5872
4	22.890	MM	0.5572	1269.85107	37.98308	44.8972

Chlorohydrin (1*R*,2*R*)-**3d** in >99% *ee* and >99:1 *dr* (bioreduction with ERED-110 and evo.1.1.200)

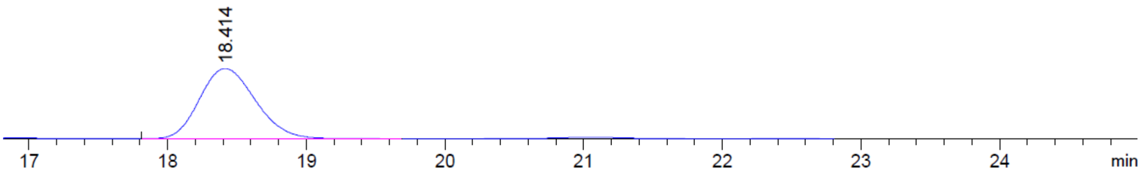

Peak #	RetTime [min]	Type	Width [min]	Area [mAU*s]	Height [mAU]	Area %
1	18.414	BB	0.4379	1.30667e4	467.77512	100.0000

Chlorohydrin (1*S*,2*R*)-**3d** in >99% *ee* and 6:94 *dr* (bioreduction with ERED P1-H09 and *Ras*ADH)

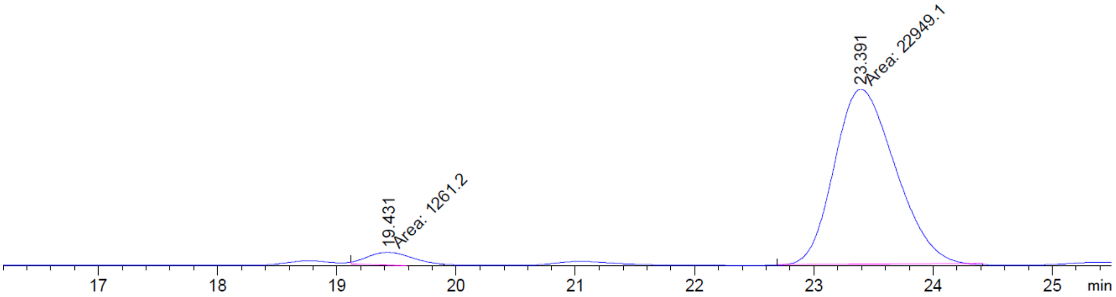

Peak #	RetTime [min]	Type	Width [min]	Area [mAU*s]	Height [mAU]	Area %
1	19.431	MM	0.4554	1261.19617	46.16052	5.2093
2	23.391	MM	0.5938	2.29491e4	644.09949	94.7907

Figure S15. HPLC chromatograms of racemic and optically active **3d** in Chiralpak AD-H column.

HPLC separation in the IC column of the *syn*-enantiomers of racemic chlorohydrin 3e

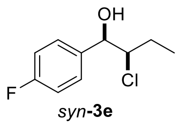

syn-**3e**

HPLC (IC, 2-propanol/*n*-hexane = 3/97, flow rate = 0.3 mL/min, l = 210 nm)

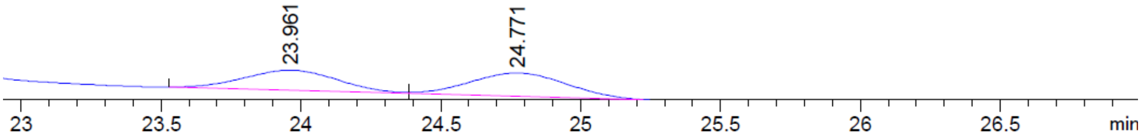

Peak #	RetTime [min]	Type	Width [min]	Area [mAU*s]	Height [mAU]	Area %
1	23.961	BV	0.3668	142.08357	6.03141	45.2383
2	24.771	VB	0.3924	171.99472	7.05455	54.7617

Chlorohydrin (1*S*,2*S*)-**3e** in >99% *ee* and 93:7 *dr* (bioreduction with ERED-110 and evo.1.1.200)

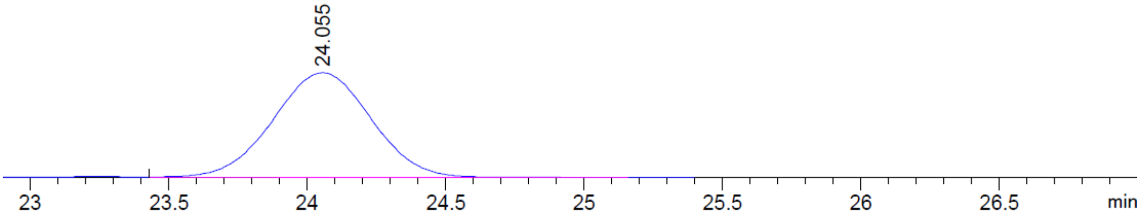

Peak #	RetTime [min]	Type	Width [min]	Area [mAU*s]	Height [mAU]	Area %
1	24.055	BB	0.3863	1.16553e4	471.59262	100.0000

Figure S16. HPLC chromatograms of racemic and optically active *syn*-**3e** in Chiralpak IC column.

HPLC separation in the AD-H column of all diastereoisomers of chlorohydrin 3f obtained via chemical reduction of the corresponding chloroketone 2f

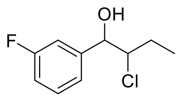

3f

HPLC (AD-H, 2-propanol/*n*-hexane = 5/95, flow rate = 0.5 mL/min, l = 210 nm)

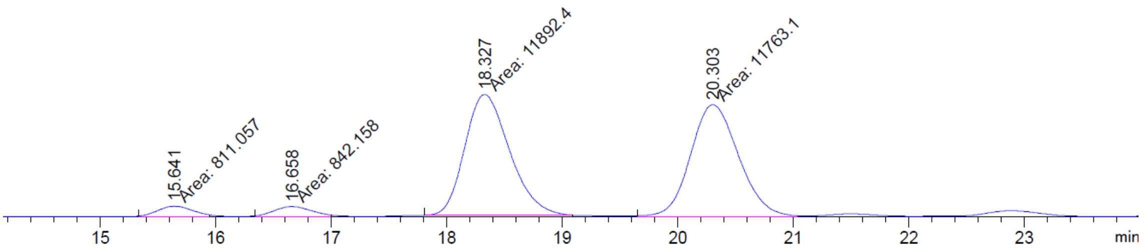

Peak #	RetTime [min]	Type	Width [min]	Area [mAU*s]	Height [mAU]	Area %
1	15.641	MM	0.3536	811.05737	38.22610	3.2047
2	16.658	MM	0.3831	842.15802	36.63995	3.3275
3	18.327	MM	0.4522	1.18924e4	438.28546	46.9893
4	20.303	MM	0.4832	1.17631e4	405.75403	46.4785

Chlorohydrin (1*R*,2*R*)-**3f** in >99% *ee* and 91:9 *dr* (bioreduction with ERED-110 and evo.1.1.200)

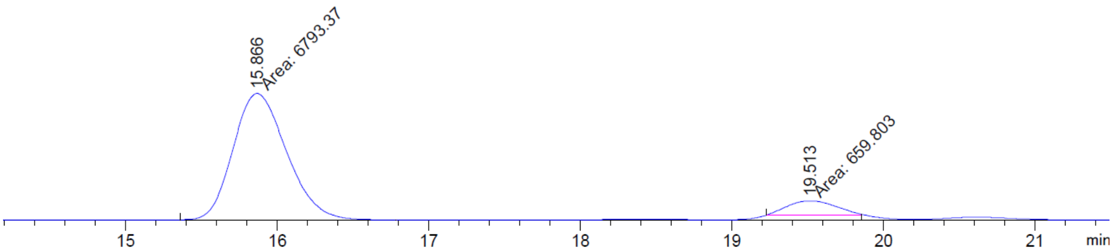

Peak #	RetTime [min]	Type	Width [min]	Area [mAU*s]	Height [mAU]	Area %
1	15.866	MM	0.4346	6793.36816	260.53162	91.1473
2	19.513	MM	0.3760	659.80334	29.24390	8.8527

Chlorohydrin (1*S*,2*R*)-**3f** in 94% *ee* and 4:96 *dr* (bioreduction with ERED P1-H09 and evo.1.1.200)

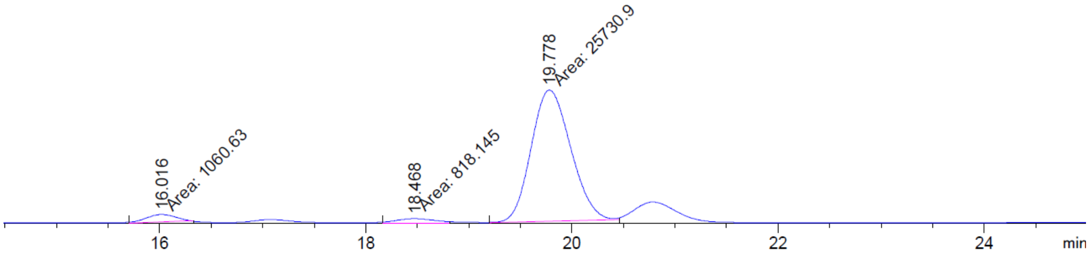

Peak #	RetTime [min]	Type	Width [min]	Area [mAU*s]	Height [mAU]	Area %
1	16.016	MM	0.3273	1060.62695	54.00610	3.8415
2	18.468	MM	0.4292	818.14459	31.76842	2.9633
3	19.778	MM	0.4433	2.57309e4	967.40588	93.1952

Figure S17. HPLC chromatograms of racemic and optically active **3f** in Chiralpak AD-H column.

HPLC separation in the AD-H column of all diastereoisomers of chlorohydrin 3g obtained via chemical reduction of the corresponding chloroketone 2g

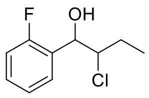

3g

HPLC (AD-H, 2-propanol/*n*-hexane = 2/98, flow rate = 0.5 mL/min, l = 210 nm)

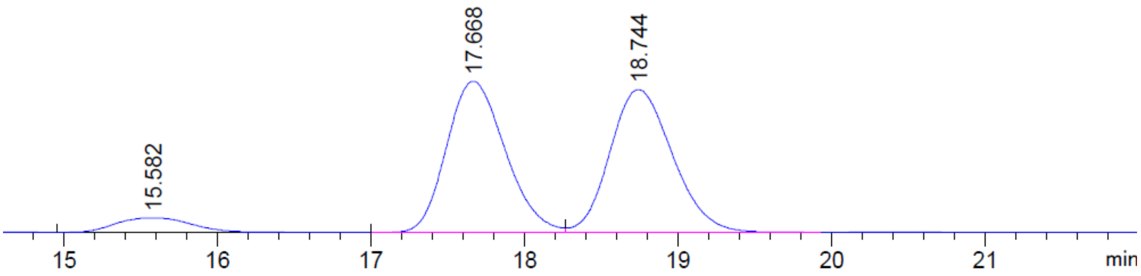

Peak #	RetTime [min]	Type	Width [min]	Area [mAU*s]	Height [mAU]	Area %
1	15.582	BB	0.5464	678.88776	19.98297	5.8213
2	17.668	BV	0.4154	5466.51172	206.16028	46.8737
3	18.744	VB	0.4425	5516.81396	194.72018	47.3050

Chlorohydrin (1*S*,2*R*)-**3g** in >99% *ee* and 93:7 *dr* (bioreduction with ERED P1-H09 and *Ras*ADH)

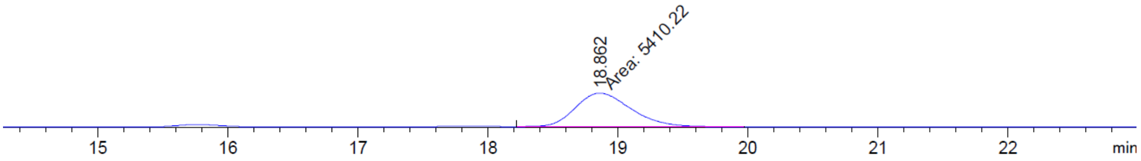

Peak #	RetTime [min]	Type	Width [min]	Area [mAU*s]	Height [mAU]	Area %
1	18.862	MM	0.4989	5410.21777	180.72664	100.0000

HPLC separation of the *syn* enantiomers of racemic chlorohydrin **3g** in Chiralcel IC column

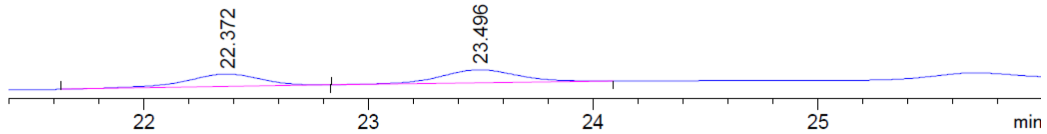

Peak #	RetTime [min]	Type	Width [min]	Area [mAU*s]	Height [mAU]	Area %
1	22.372	BB	0.3367	45.47656	1.94580	48.5765
2	23.496	BB	0.3525	48.14188	2.01706	51.4235

Chlorohydrin (1*R*,2*R*)-**3g** in >99% *ee* and 97:3 *dr* (bioreduction with ERED-110 and evo.1.1.200)

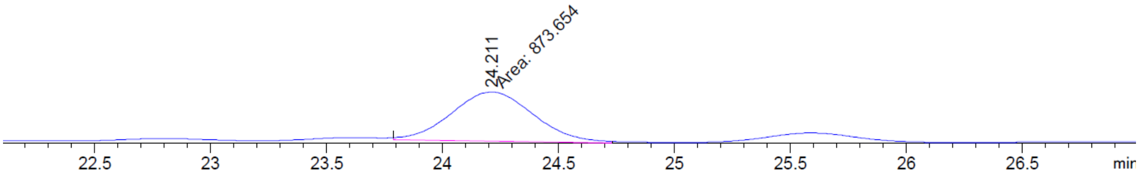

Peak #	RetTime [min]	Type	Width [min]	Area [mAU*s]	Height [mAU]	Area %
1	24.211	MM	0.4115	873.65369	35.38240	100.0000

Figure S18. HPLC chromatograms of racemic and optically active **3g** and *syn*-**3g** in Chiralpak AD-H and Chiralpak IC columns.

VII.3. NMR spectra

2-Chloro-1-(3-fluorophenyl)but-3-en-1-ol (7f)

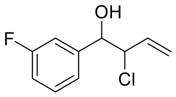

7f

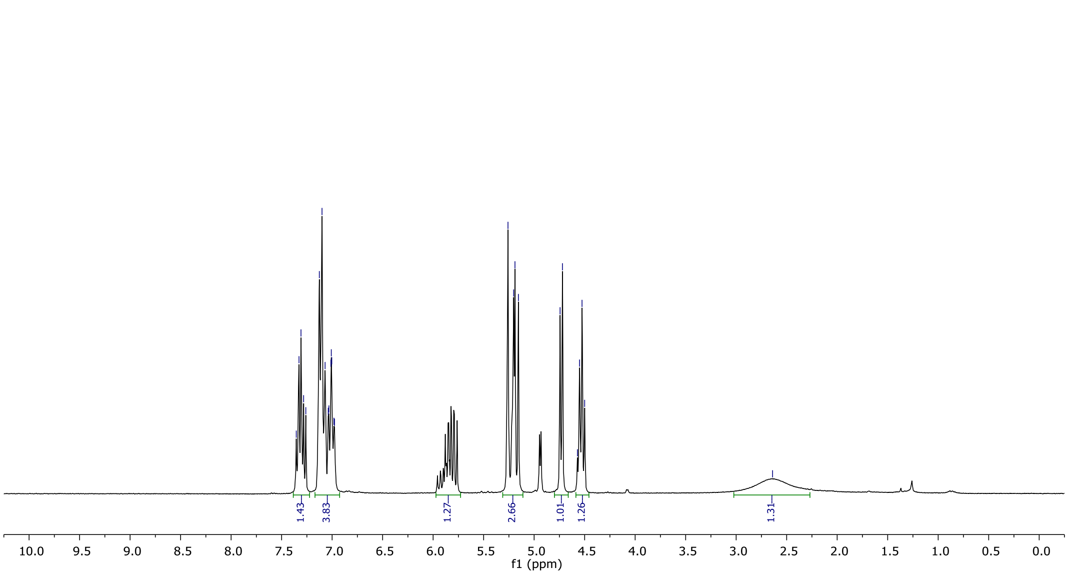

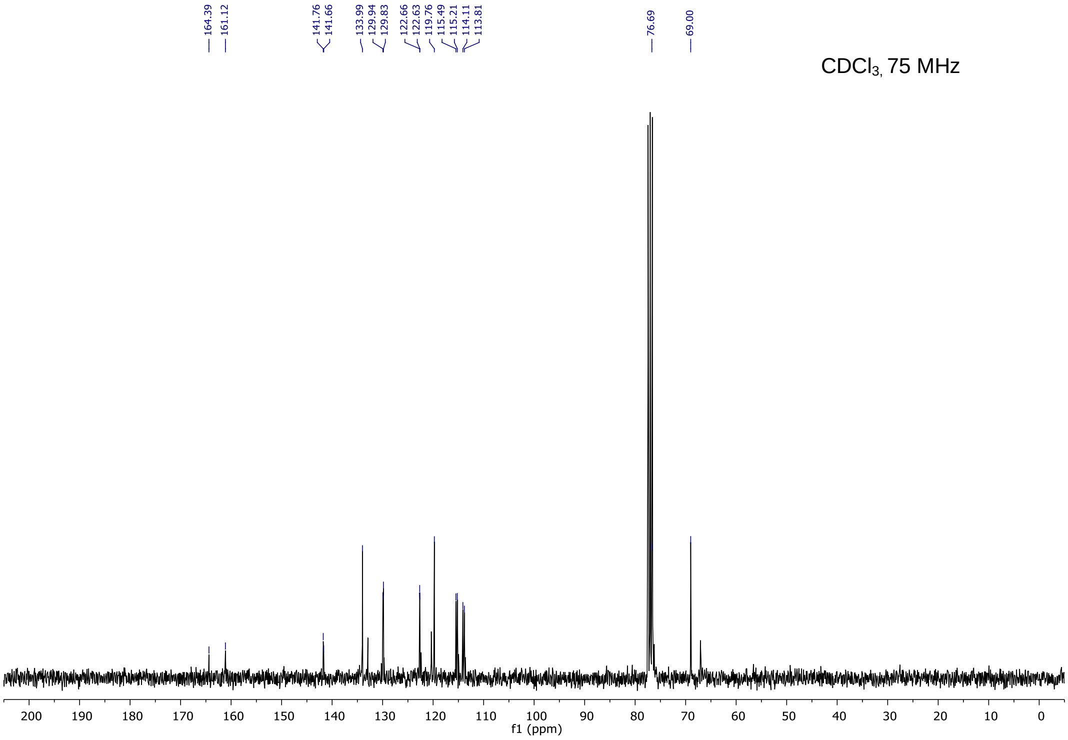

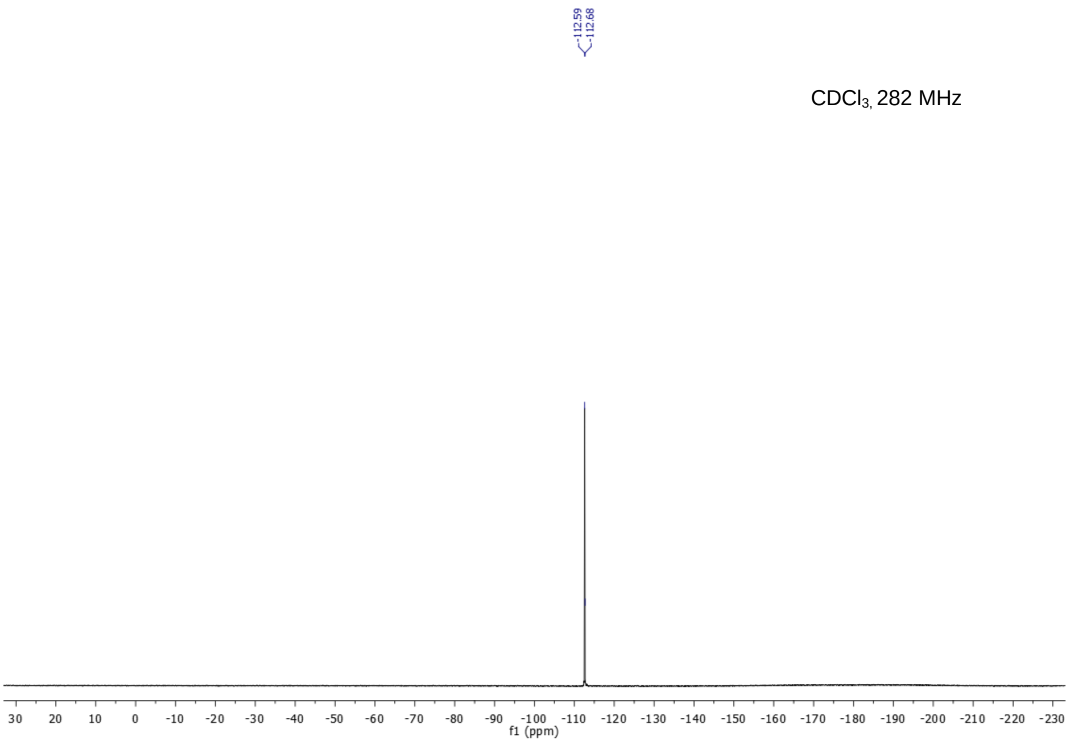

Figure S19. ^{1}H, ^{13}C and ^{19}F NMR spectra ($CDCl_3$) of compound **7f**.

2-Chloro-1-(2-fluorophenyl)but-3-en-1-ol (7g)

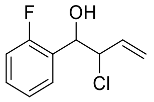

7g

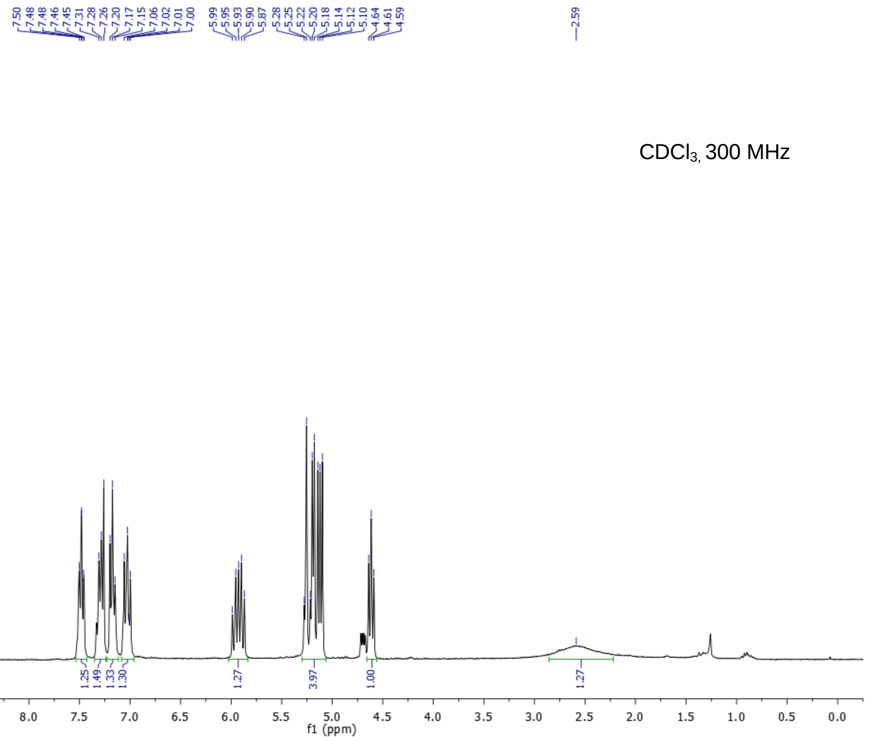

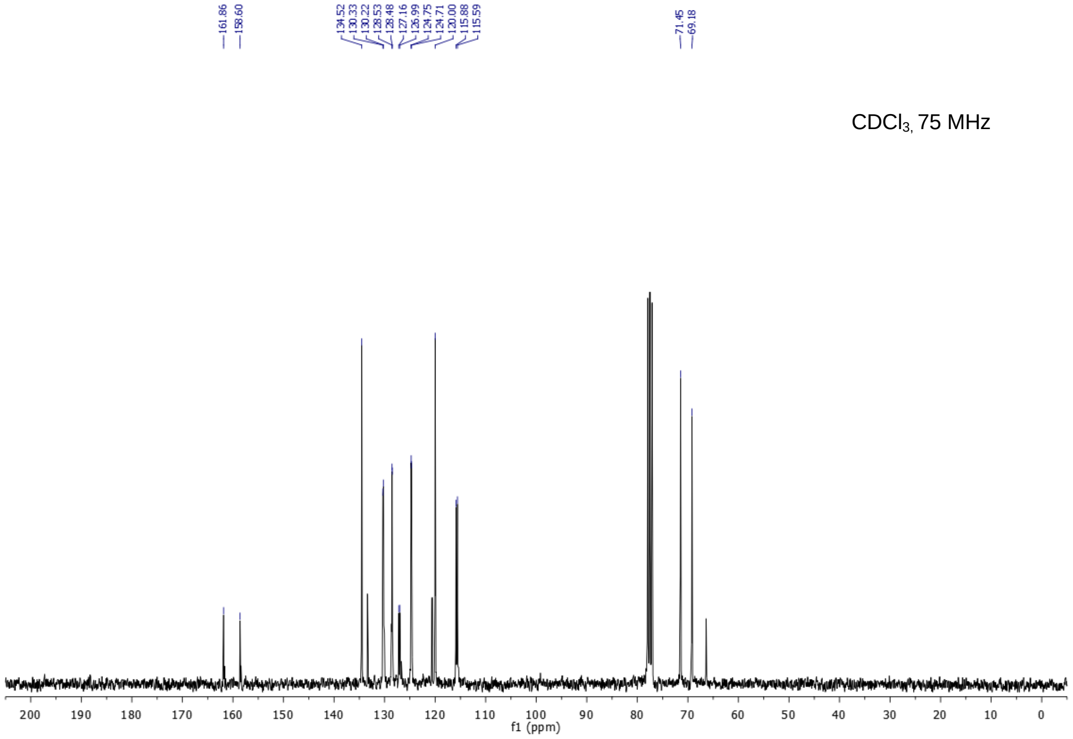

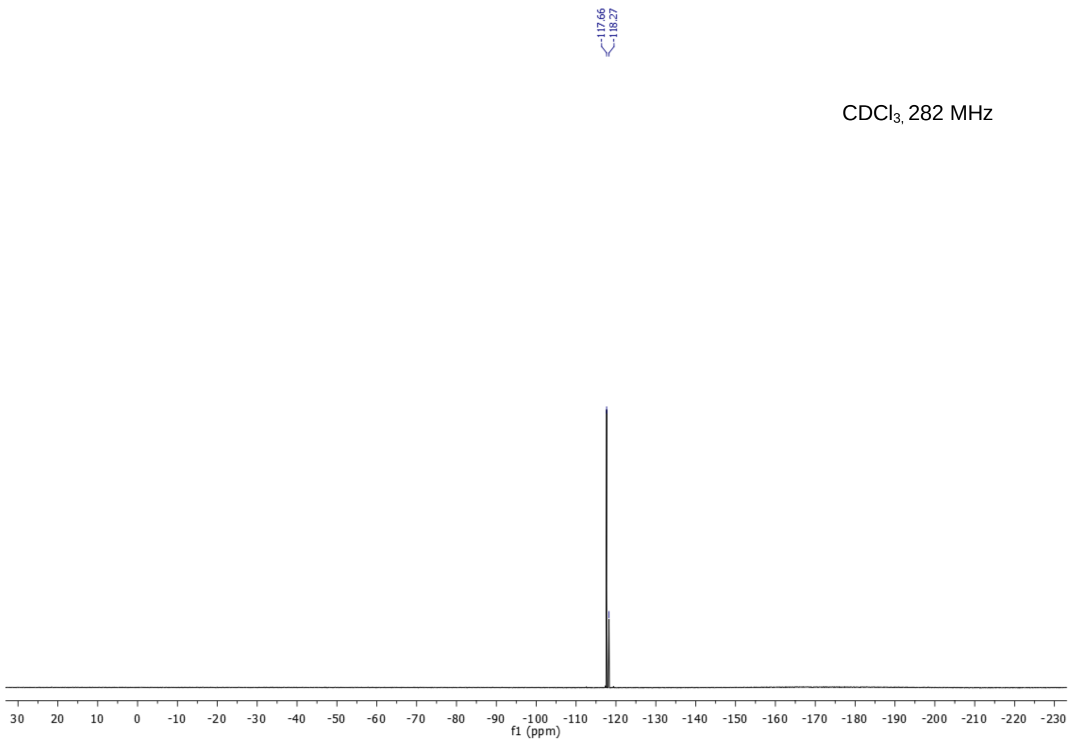

Figure S20. ^{1}H, ^{13}C and ^{19}F NMR spectra ($CDCl_3$) of compound **7g**.

(*Z*)-2-Chloro-1-(4-methylphenyl)but-2-en-1-one (1b)

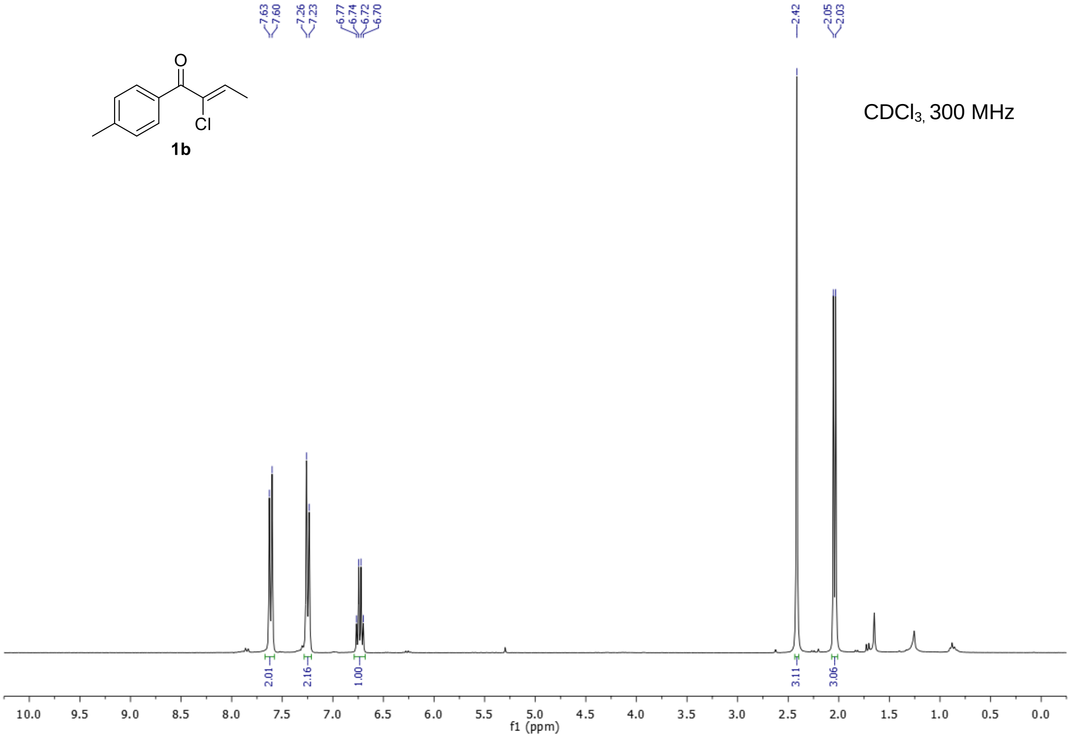

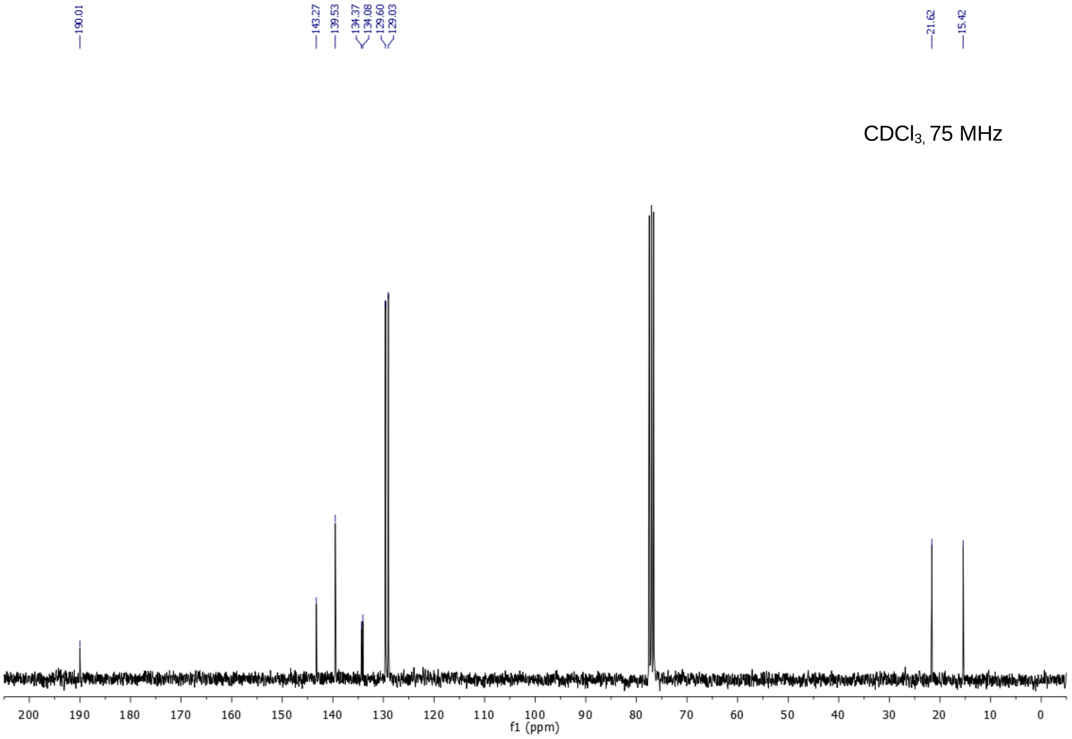

Figure S21. ^{1}H and ^{13}C NMR spectra ($CDCl_3$) of compound **1b**.

(*Z*)-2-Chloro-1-(4-chlorophenyl)but-2-en-1-one (1c)

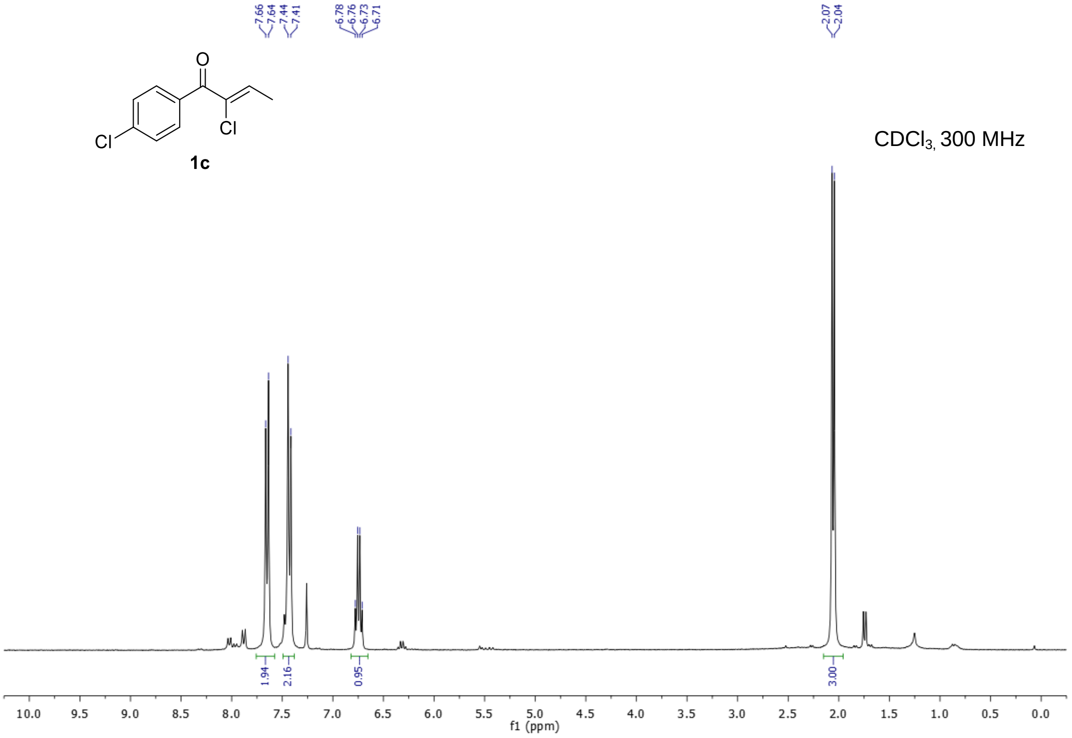

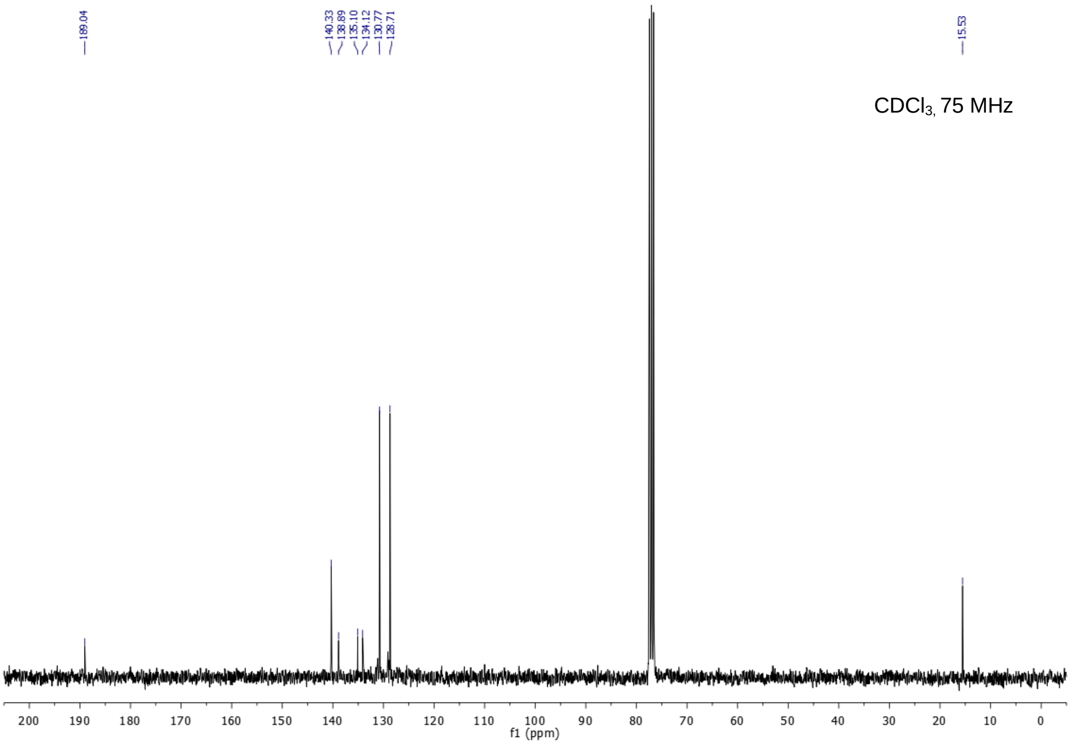

Figure S22. ^{1}H and ^{13}C NMR spectra ($CDCl_3$) of compound **1c**.

(*Z*)-1-(4-Bromophenyl)-2-chlorobut-2-en-1-one (1d)

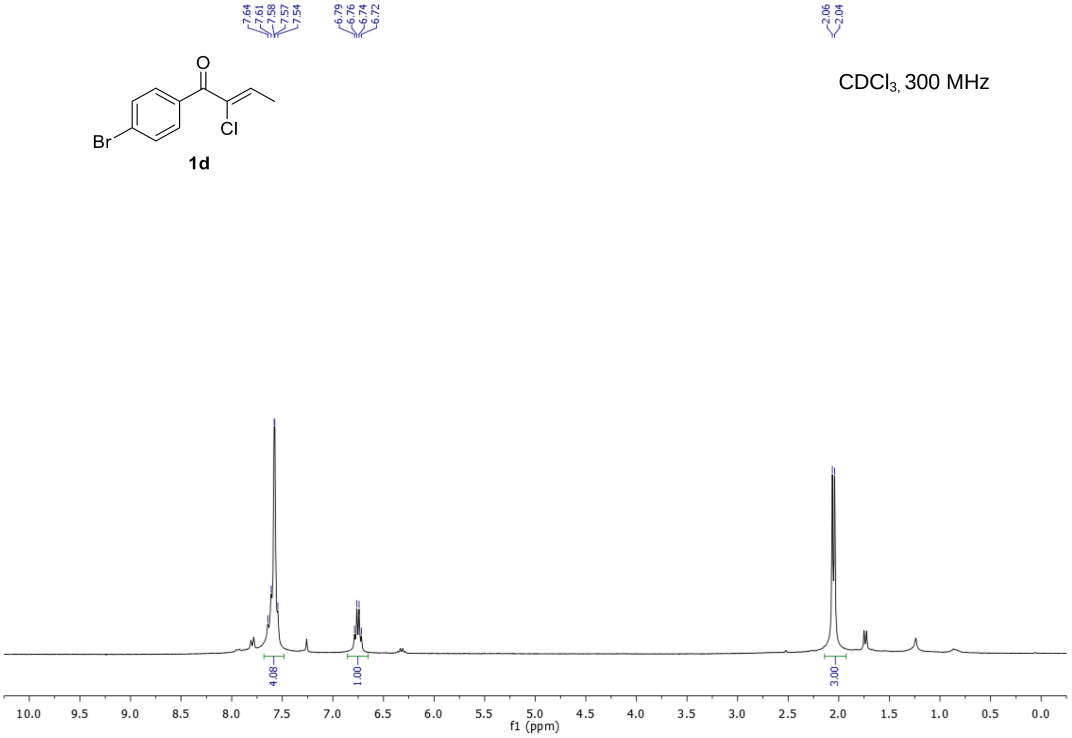

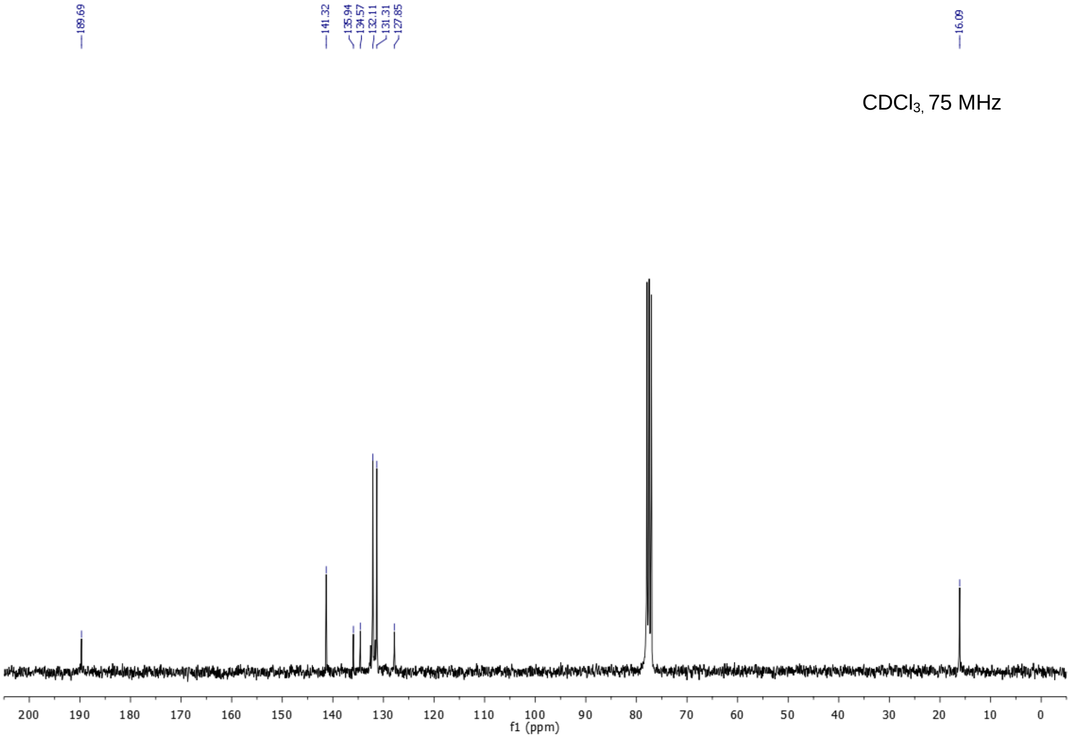

Figure S23. ^{1}H and ^{13}C NMR spectra ($CDCl_3$) of compound **1d**.

(*Z*)-2-Chloro-1-(4-fluorophenyl)but-2-en-1-one (1e)

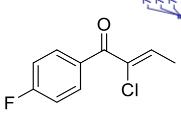

1e

CDCl$_3$, 300 MHz

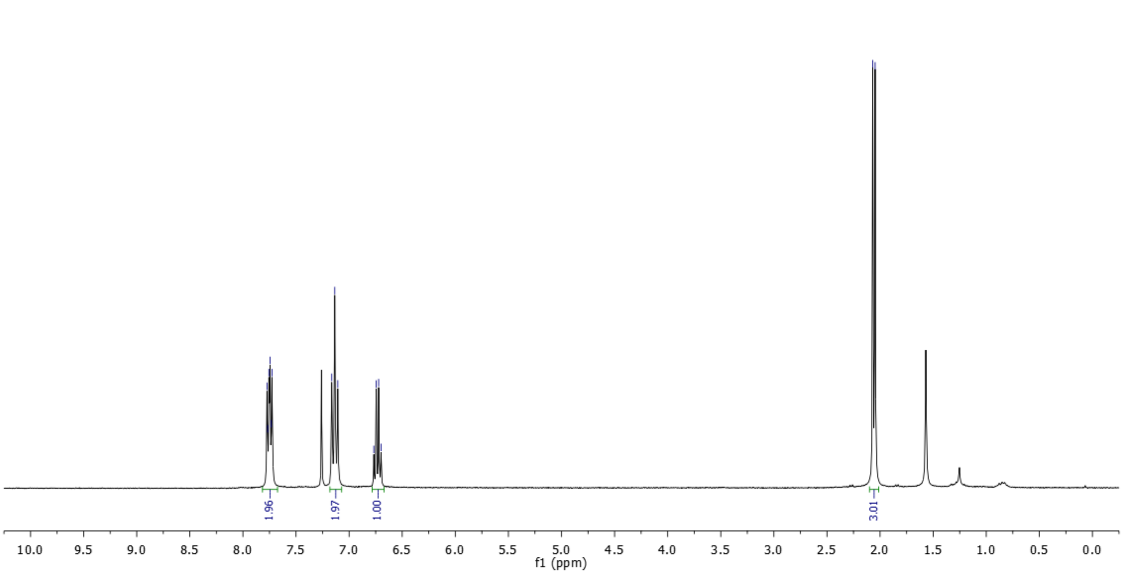

CDCl$_3$, 75 MHz

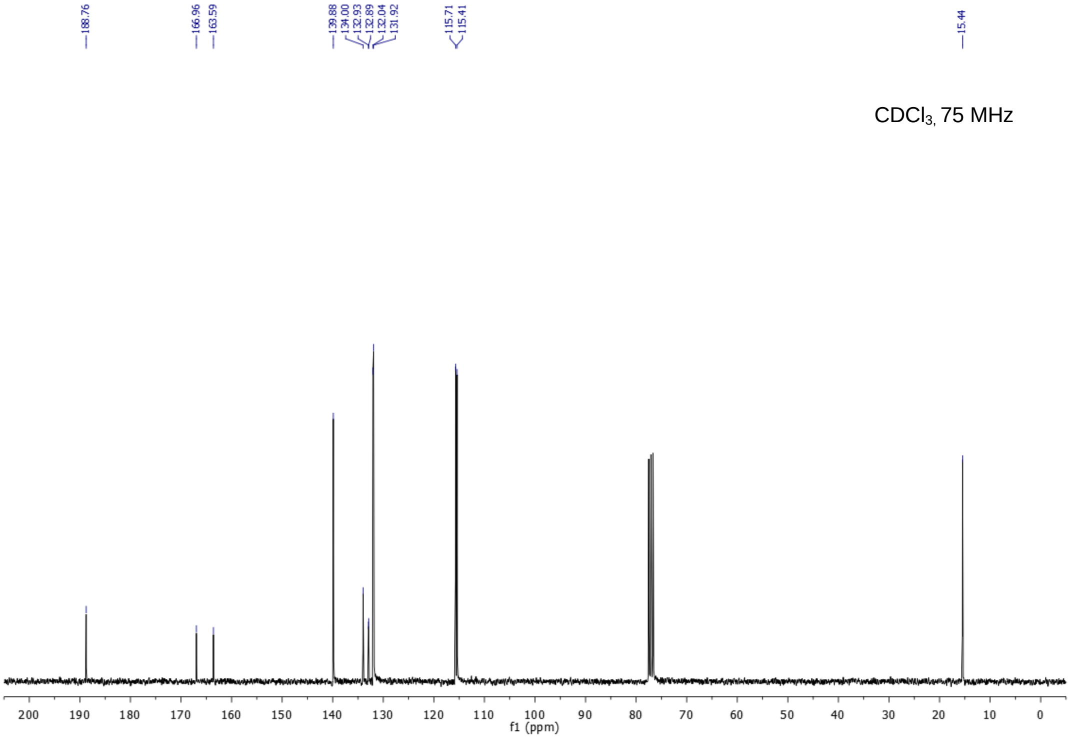

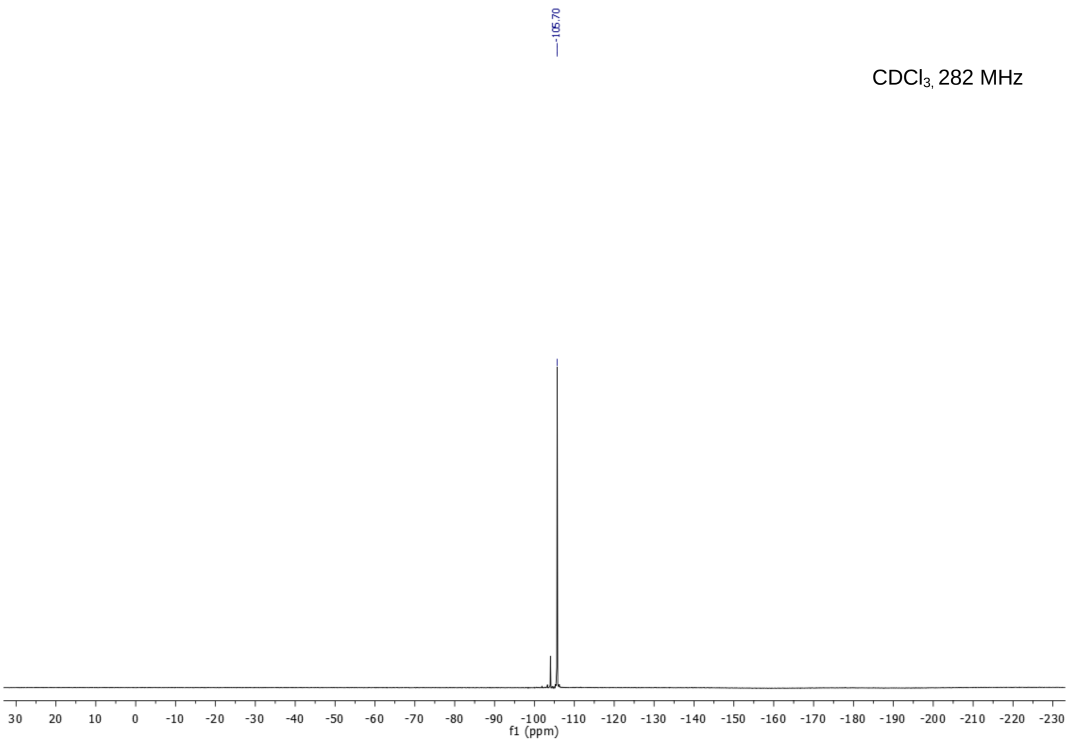

Figure S24. ^{1}H, ^{13}C and 19F NMR spectra ($CDCl_3$) of compound **1e**.

(*Z*)-2-Chloro-1-(3-fluorophenyl)but-2-en-1-one (1f)

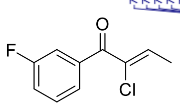

1f

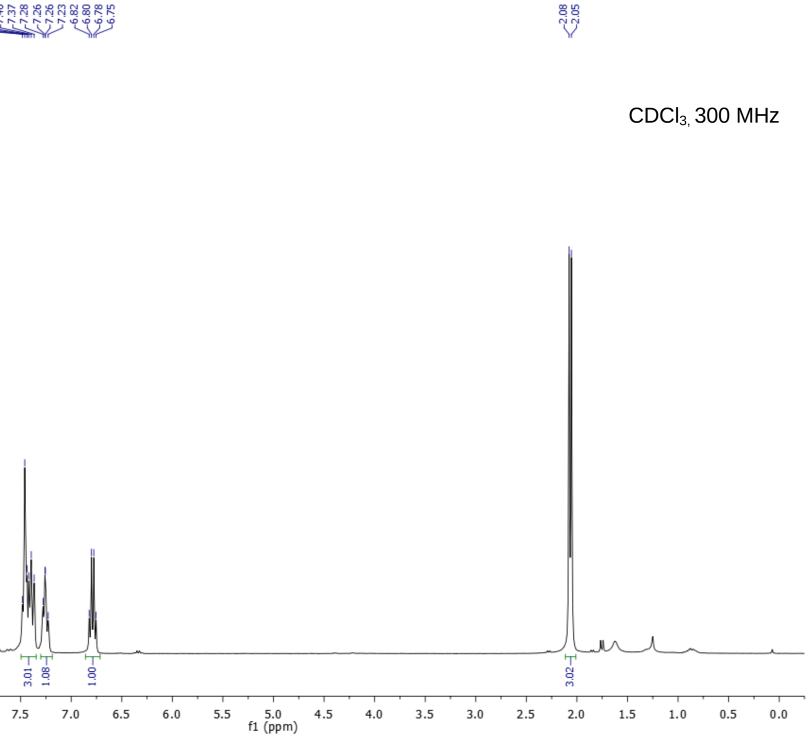

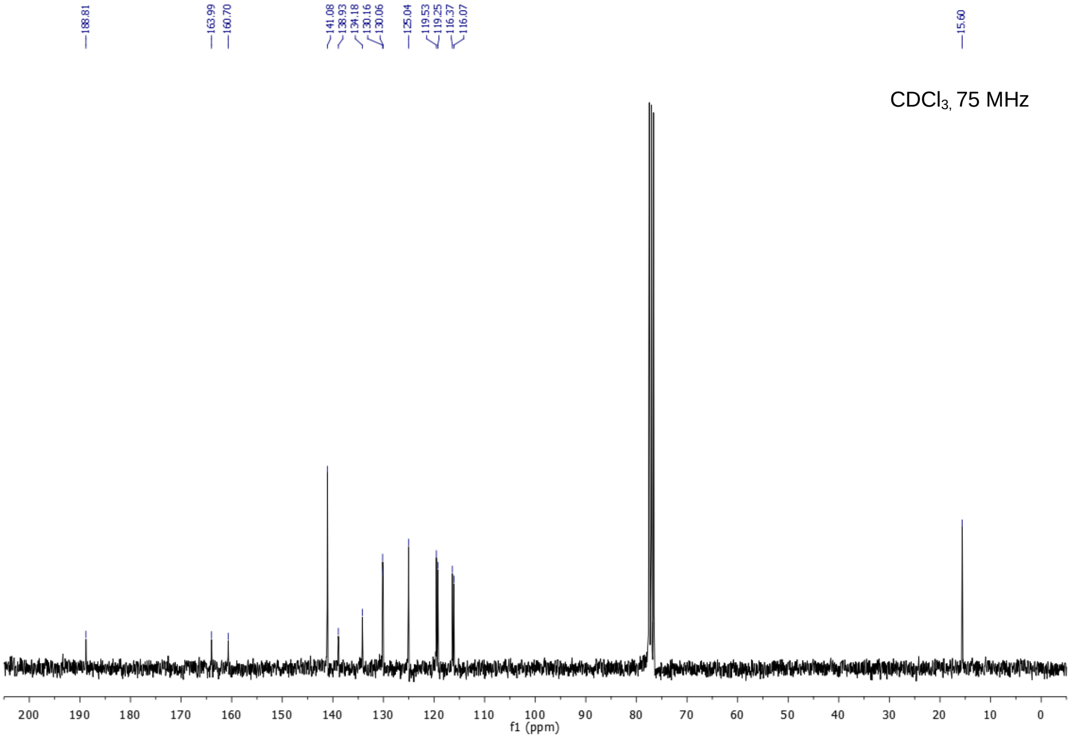

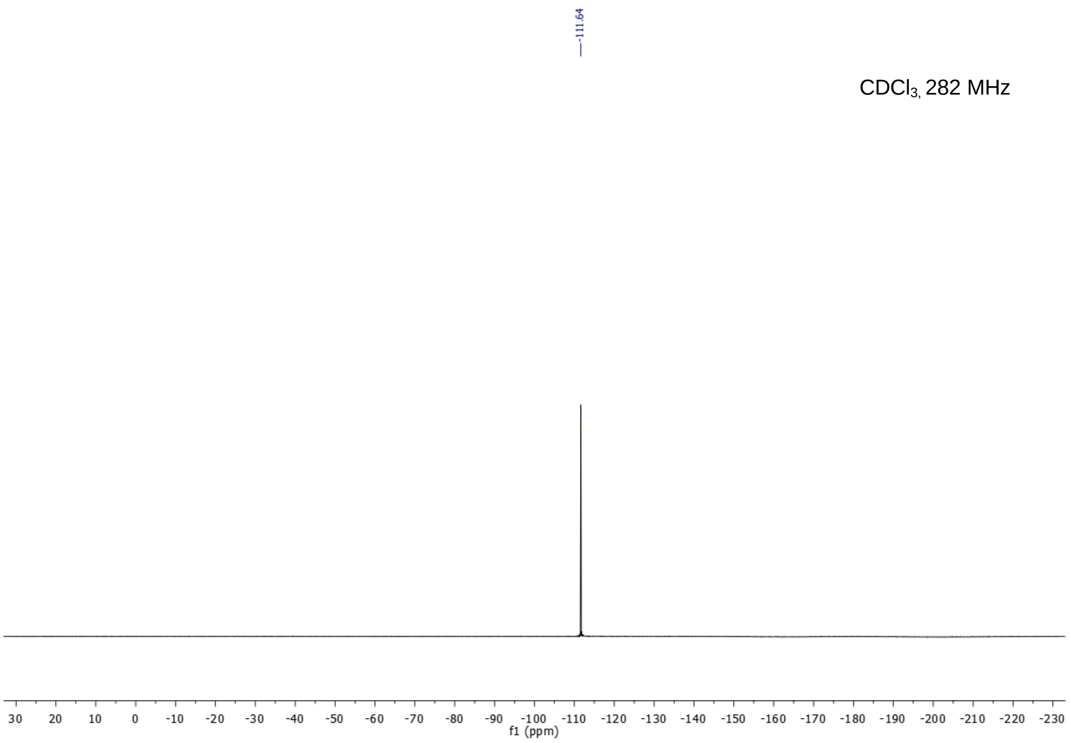

Figure S25. ^{1}H, ^{13}C and ^{19}F NMR spectra ($CDCl_3$) of compound **1f**.

(*Z*)-2-Chloro-1-(2-fluorophenyl)but-2-en-1-one (1g)

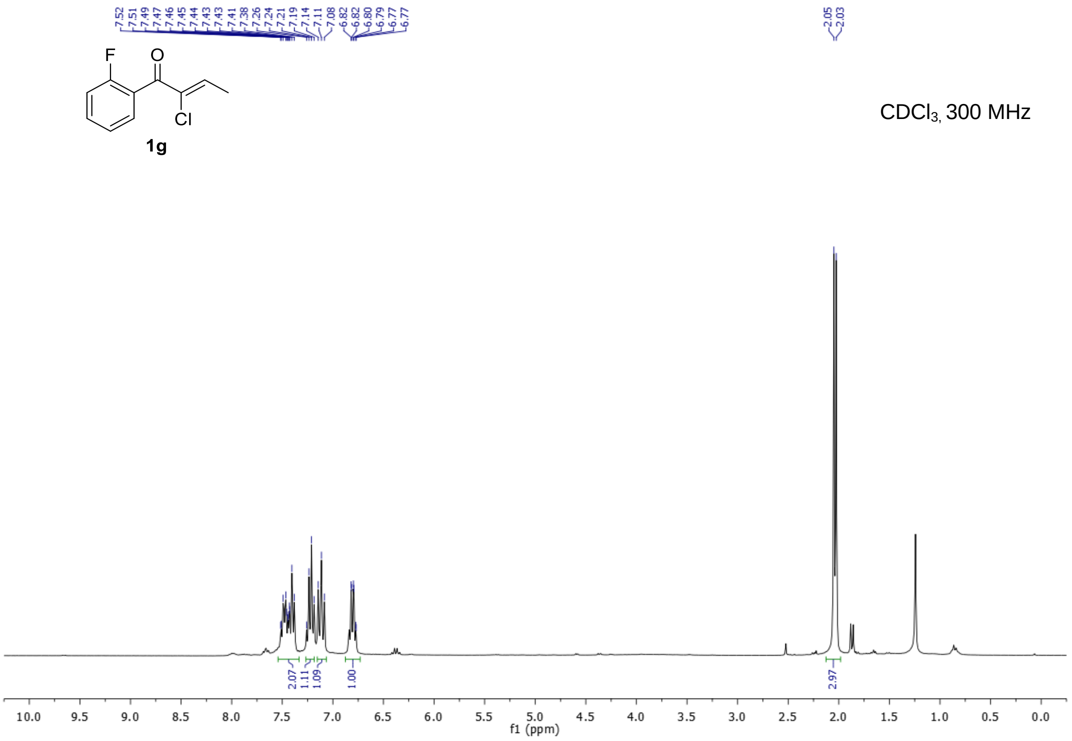

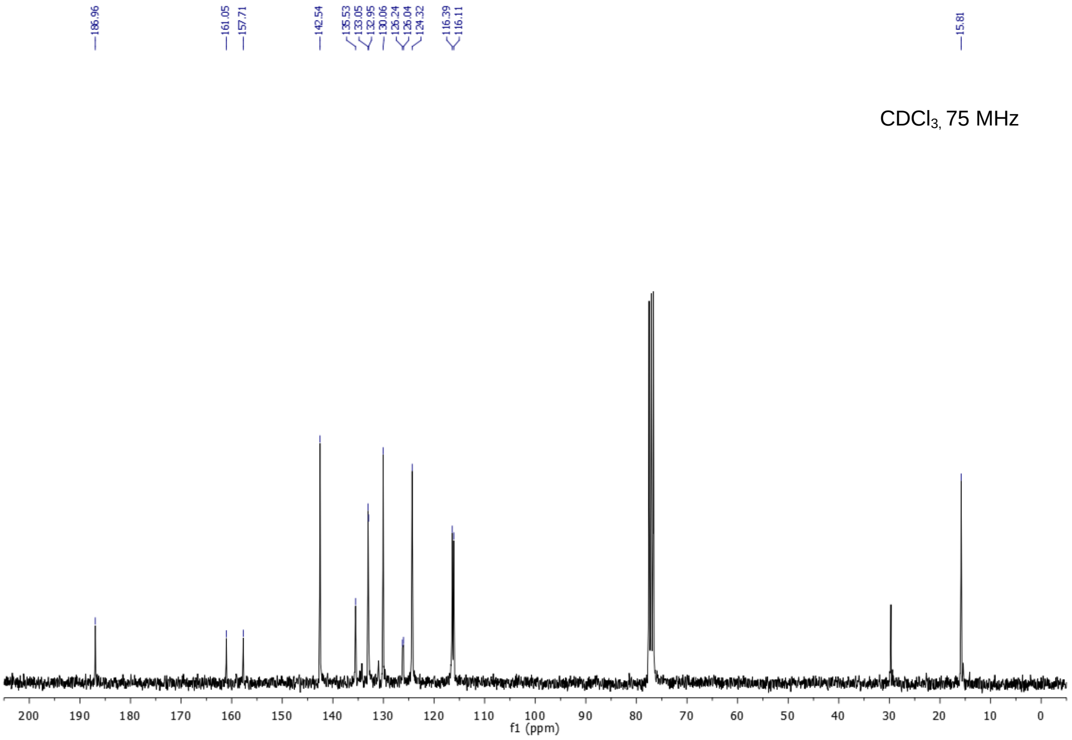

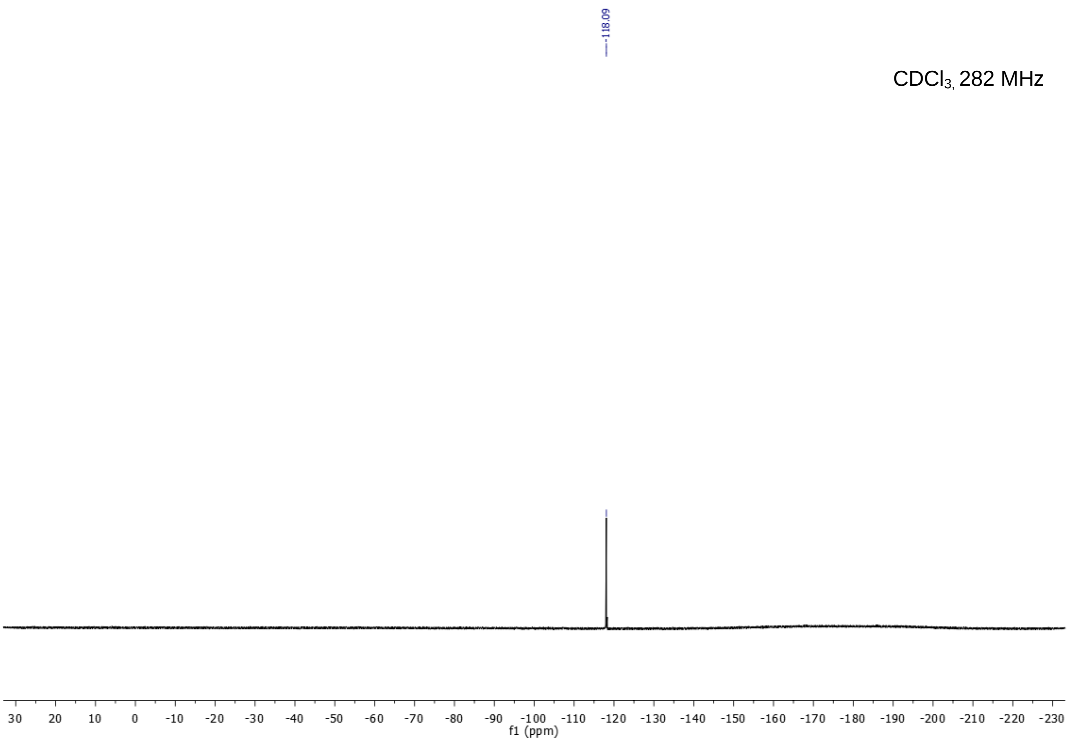

Figure S26. ^{1}H, ^{13}C NMR and ^{19}F NMR spectra ($CDCl_3$) of compound **1g**.

1-(2-Fluorophenyl)butan-1-ol (8g)

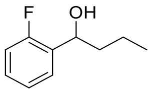

8g

CDCl$_3$, 300 MHz

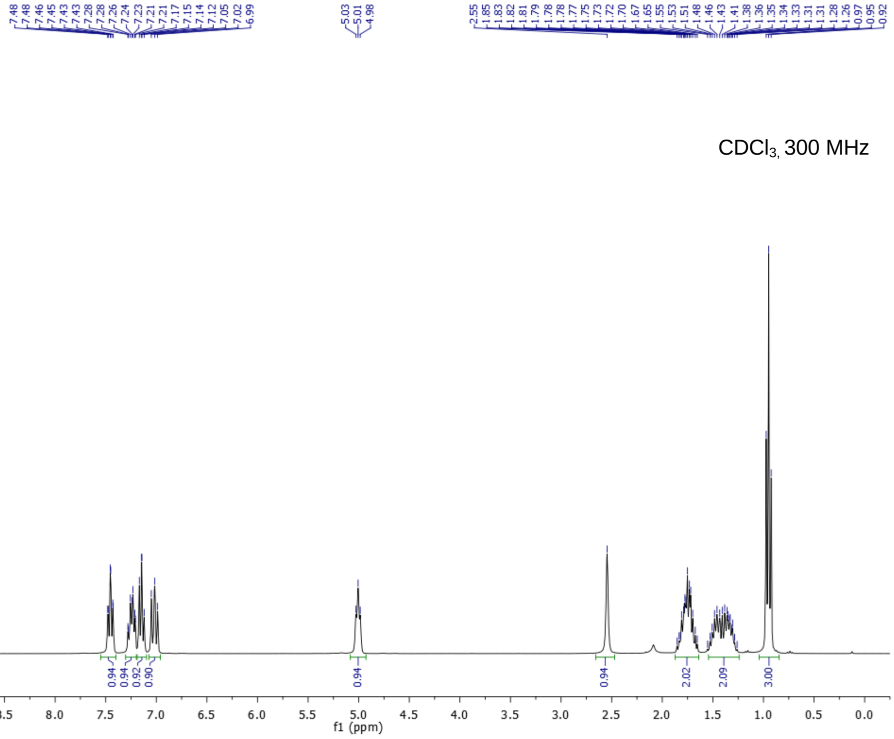

CDCl$_3$, 75 MHz

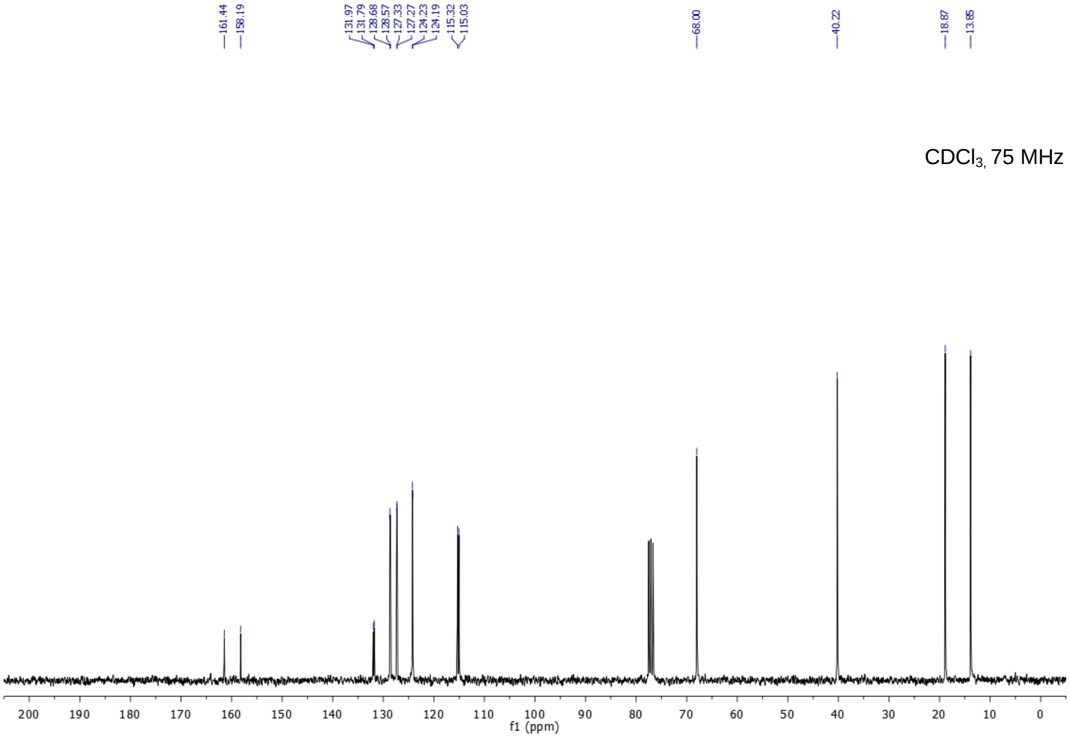

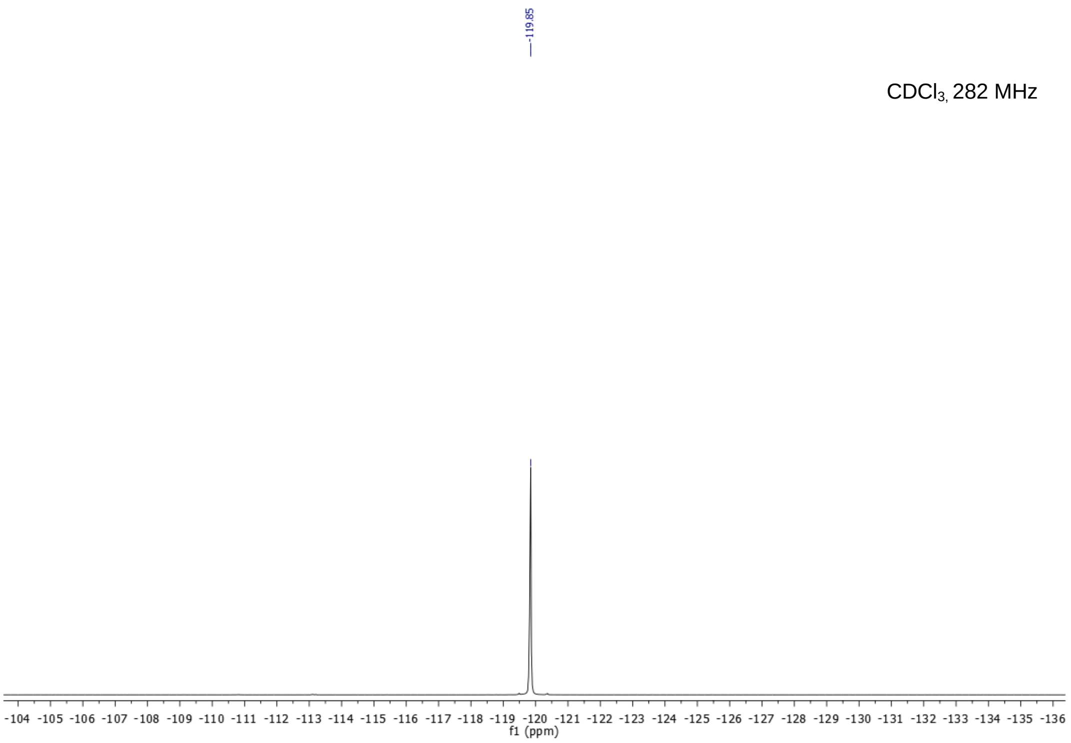

Figure S27. ^{1}H, ^{13}C and 19F NMR spectra ($CDCl_3$) of compound **8g**.

2-Chloro-1-(4-methylphenyl)butan-1-one (2b)

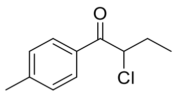

2b

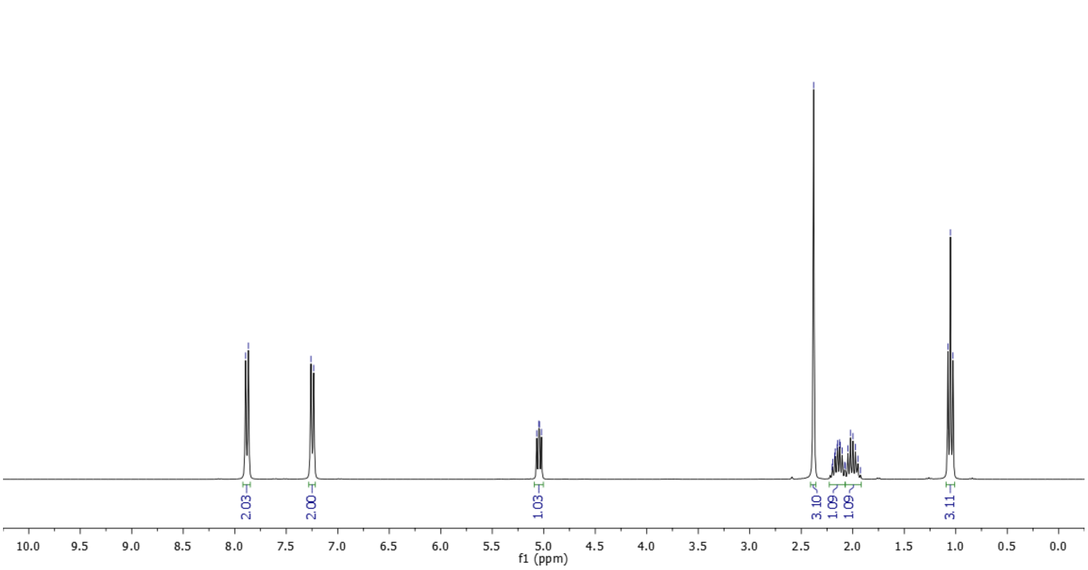

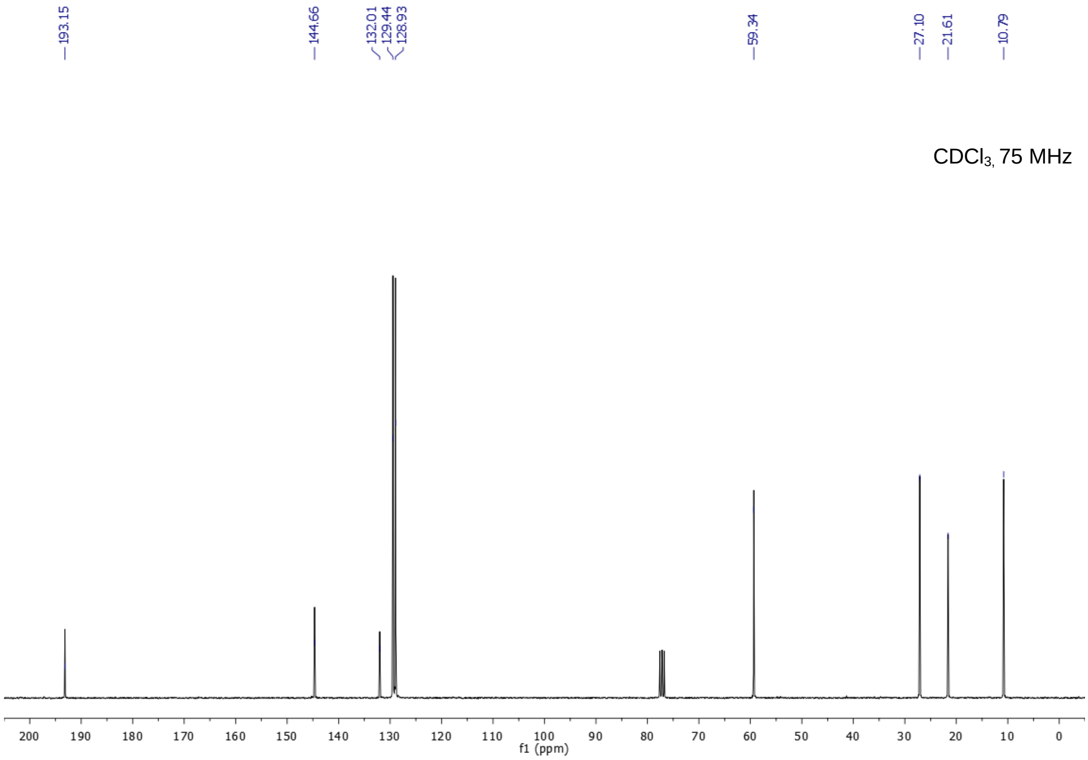

Figure S28. ^{1}H and ^{13}C NMR spectra ($CDCl_3$) of compound **2b**.

2-Chloro-1-(4-chlorophenyl)butan-1-one (2c)

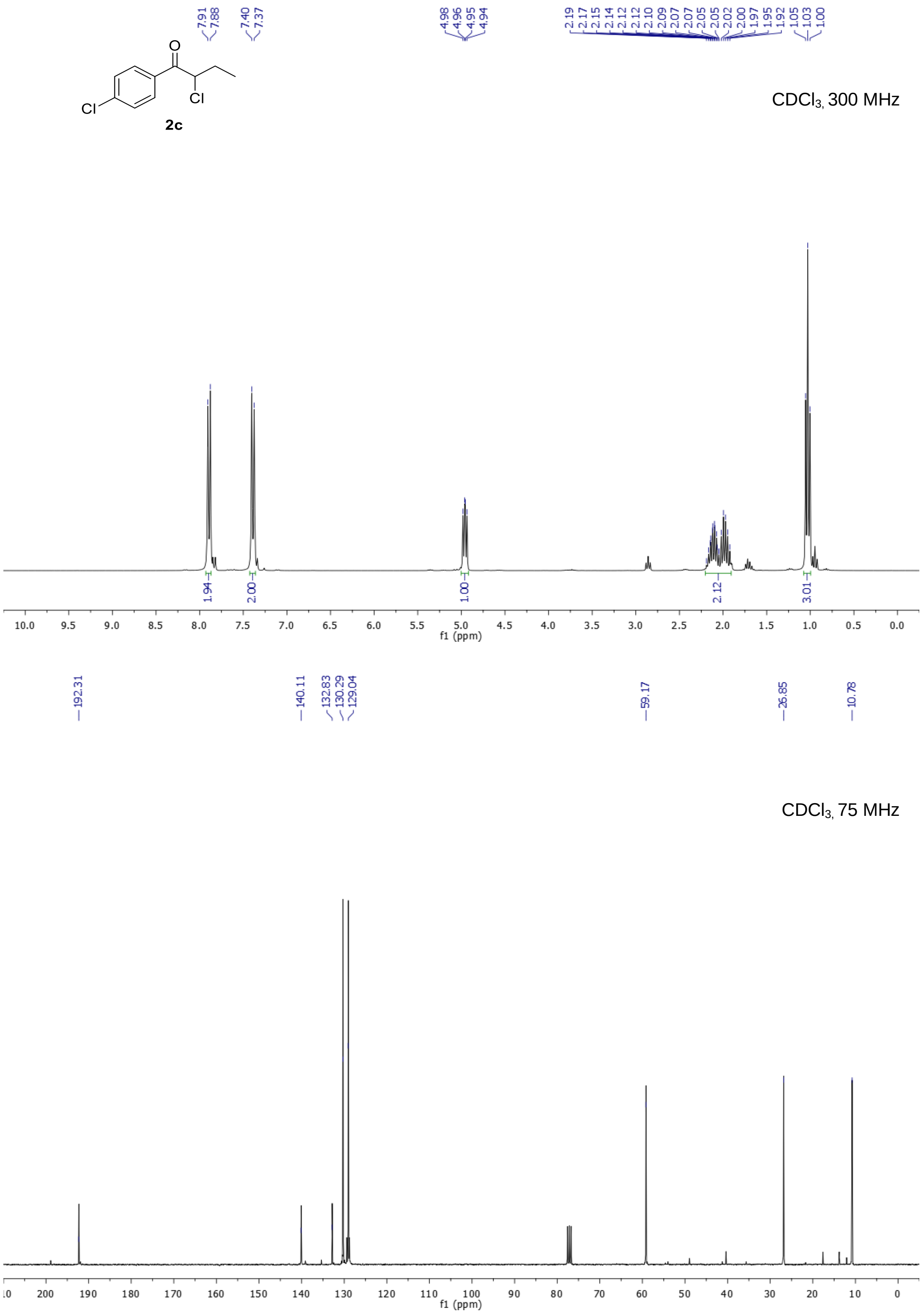

Figure S29. ^{1}H and ^{13}C NMR spectra ($CDCl_3$) of compound **2c**.

1-(4-Bromophenyl)-2-chlorobutan-1-one (2d)

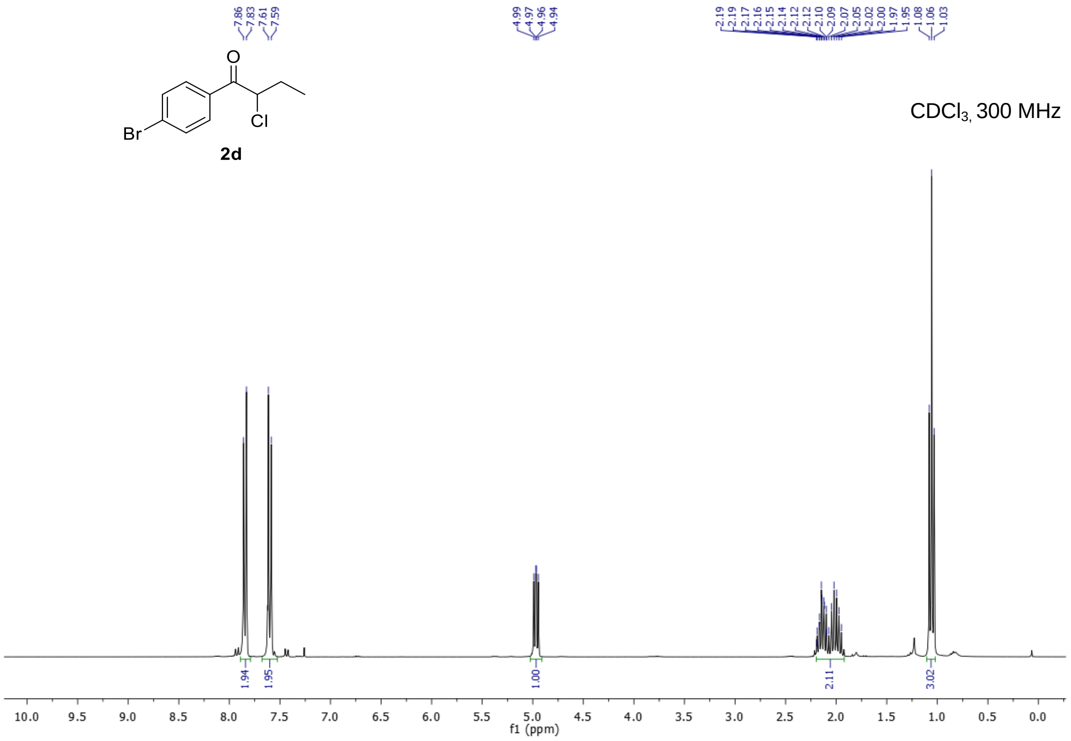

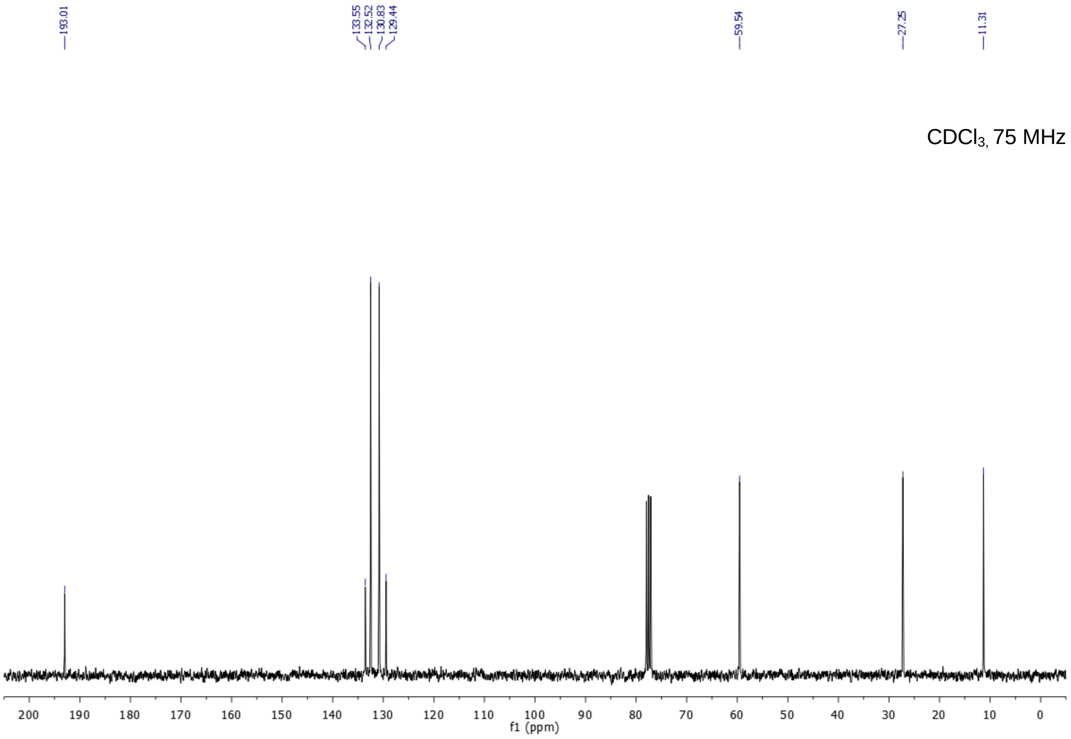

Figure S30. ^{1}H and ^{13}C NMR spectra ($CDCl_3$) of compound **2d**.

2-Chloro-1-(4-fluorophenyl)butan-1-one (2e)

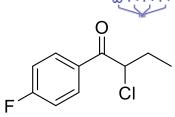

2e

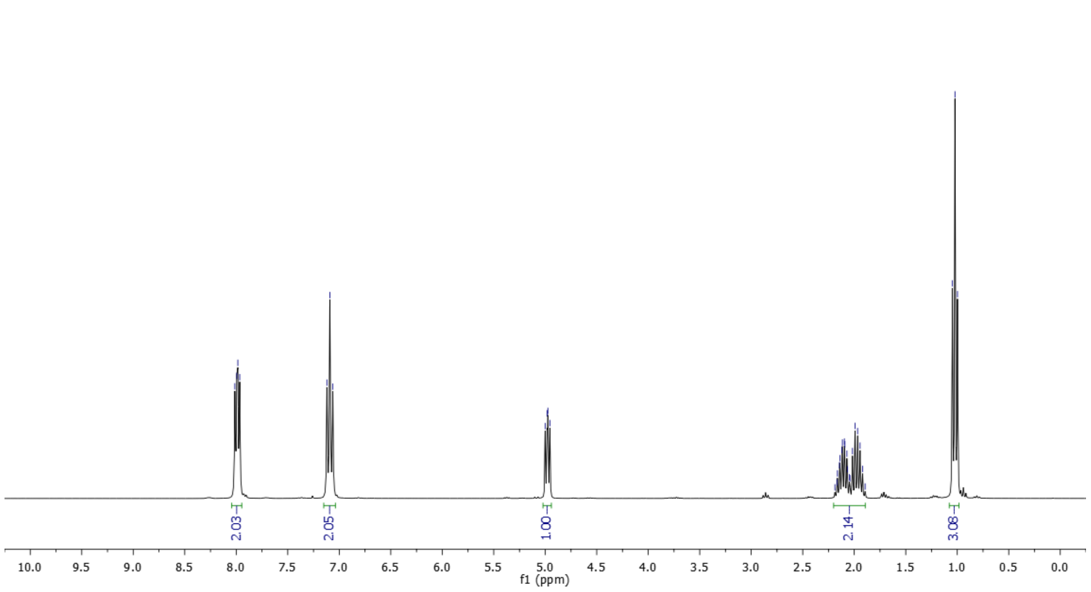

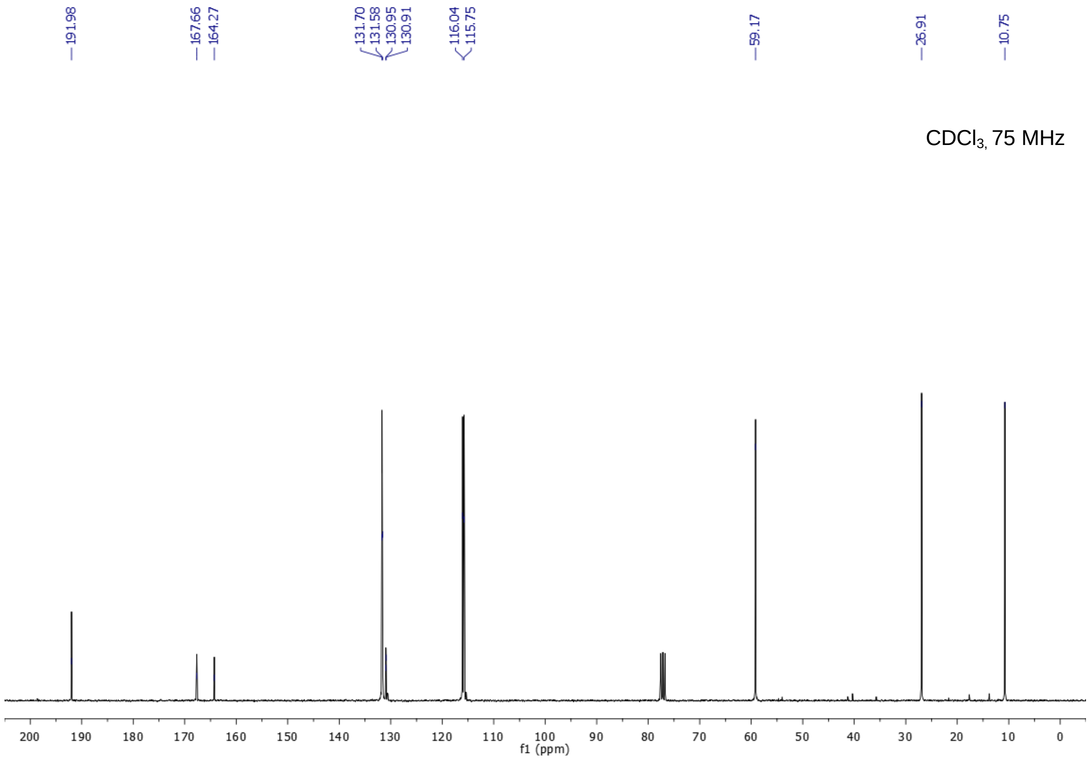

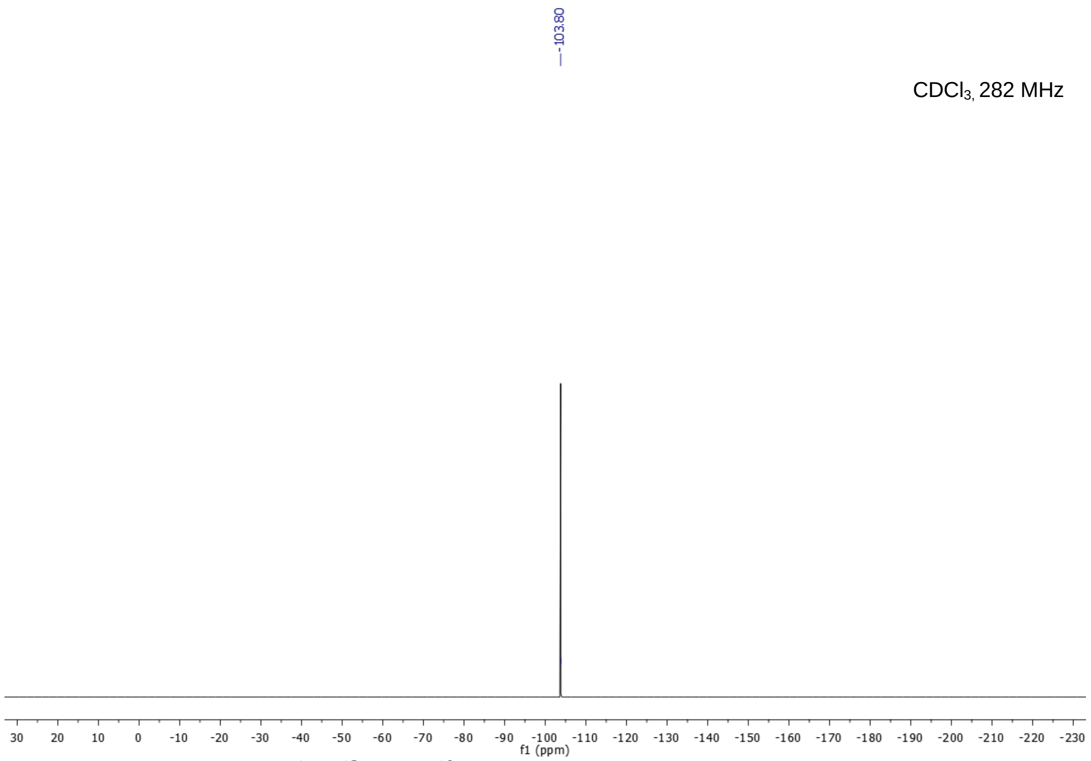

Figure S31. ^{1}H, ^{13}C and ^{19}F NMR spectra ($CDCl_3$) of compound **2e**.

2-Chloro-1-(3-fluorophenyl)butan-1-one (2f)

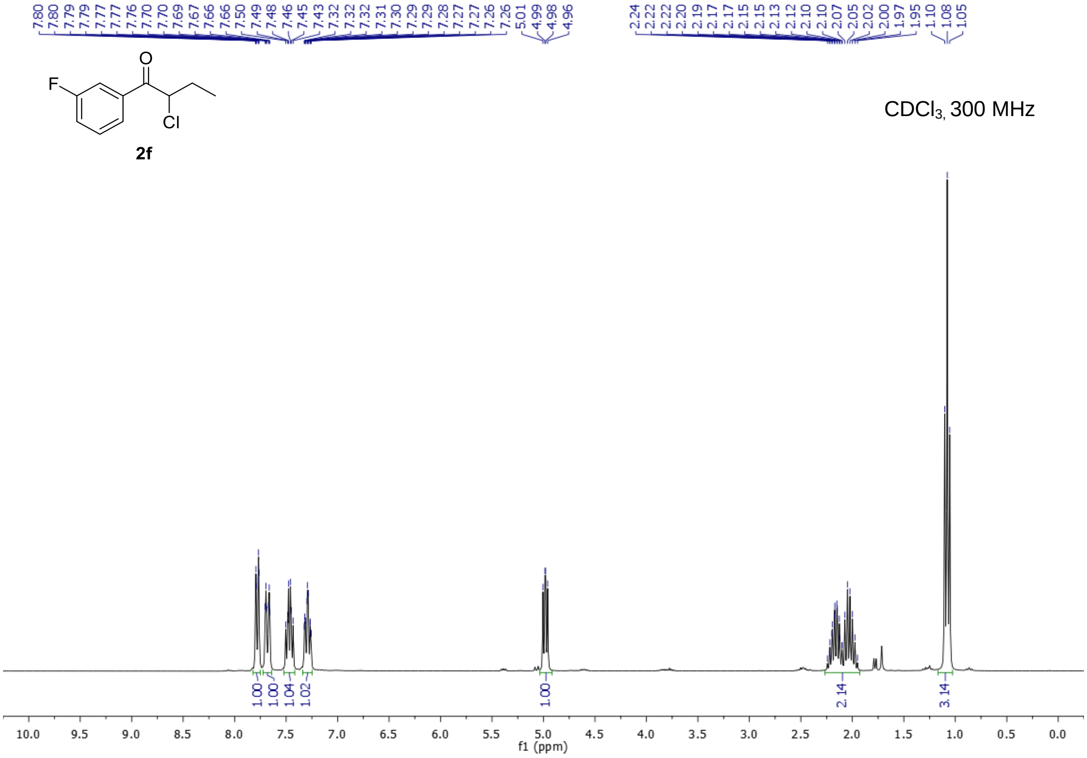

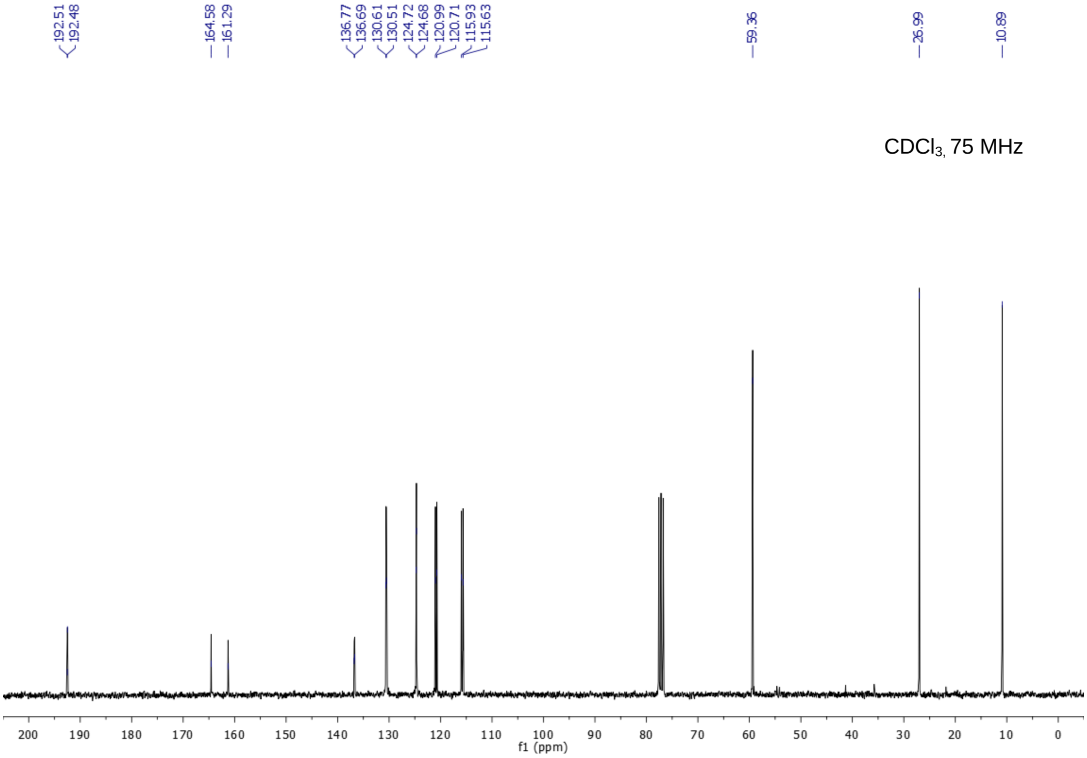

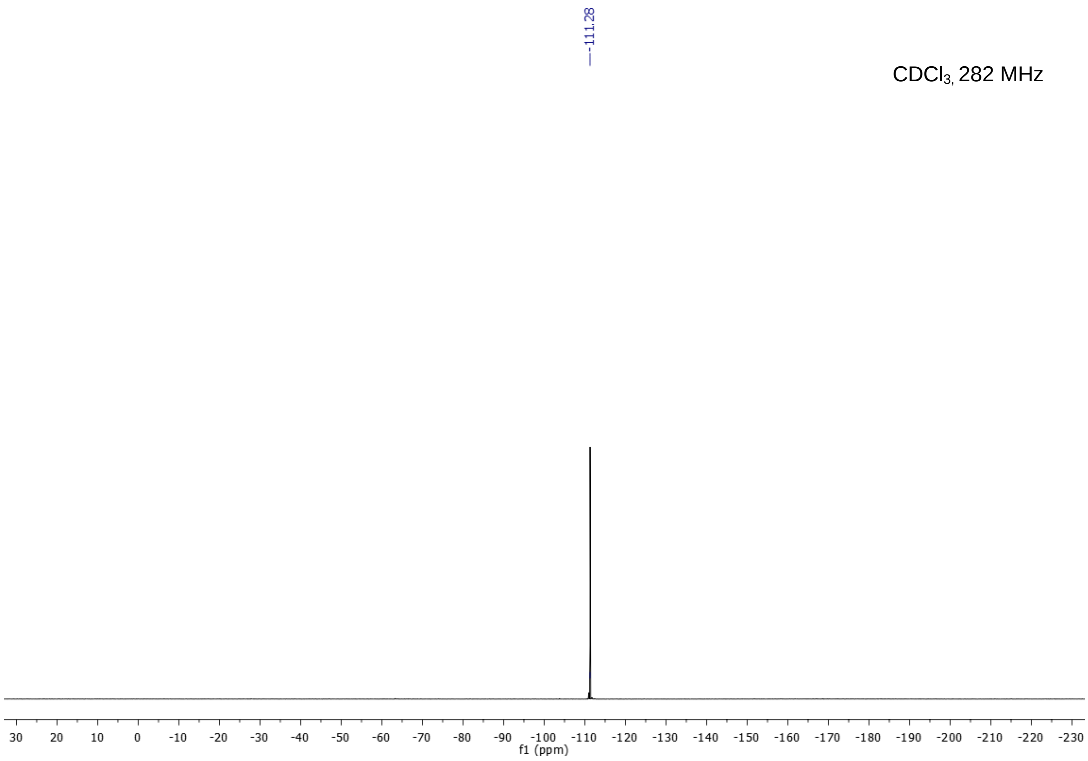

Figure S32. ^{1}H, ^{13}C and ^{19}F NMR spectra ($CDCl_3$) of compound **2f**.

2-Chloro-1-(2-fluorophenyl)butan-1-one (2g)

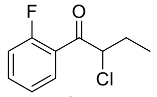

2g

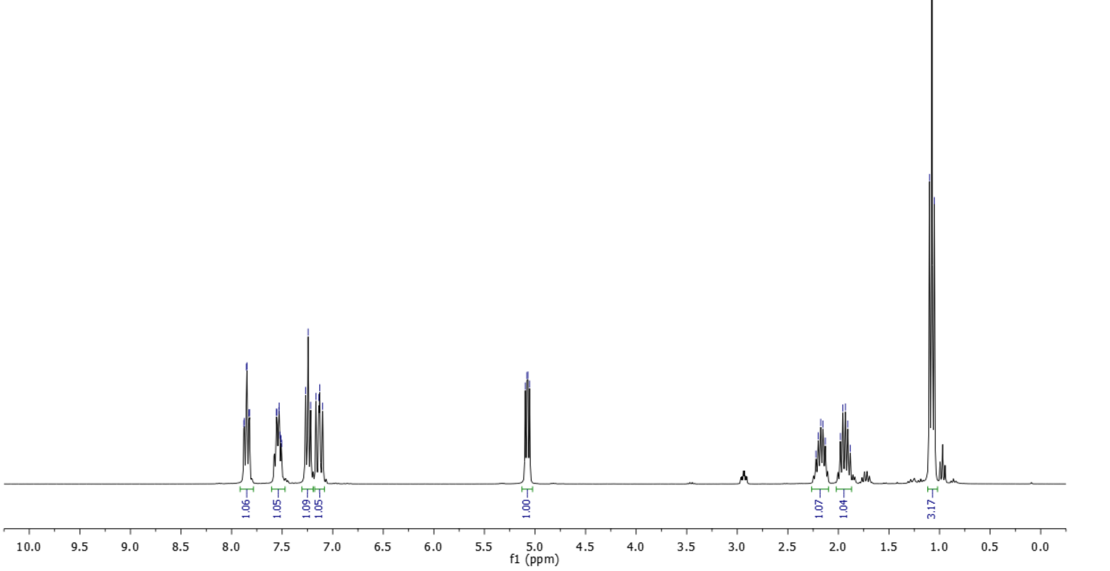

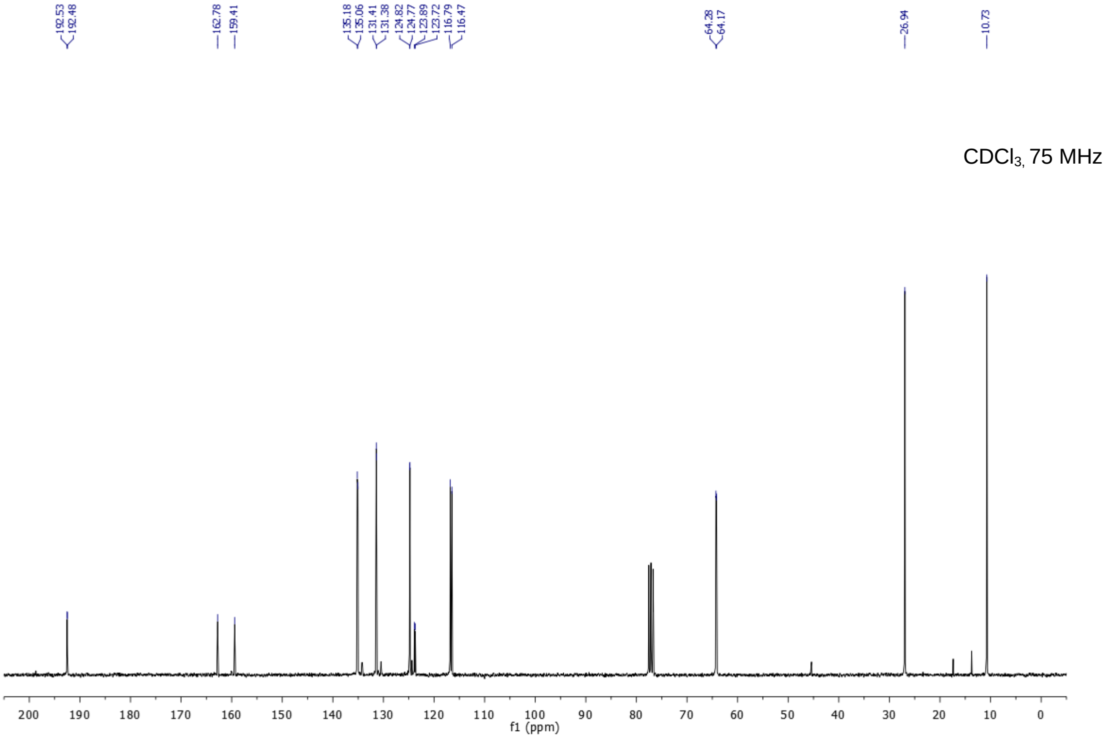

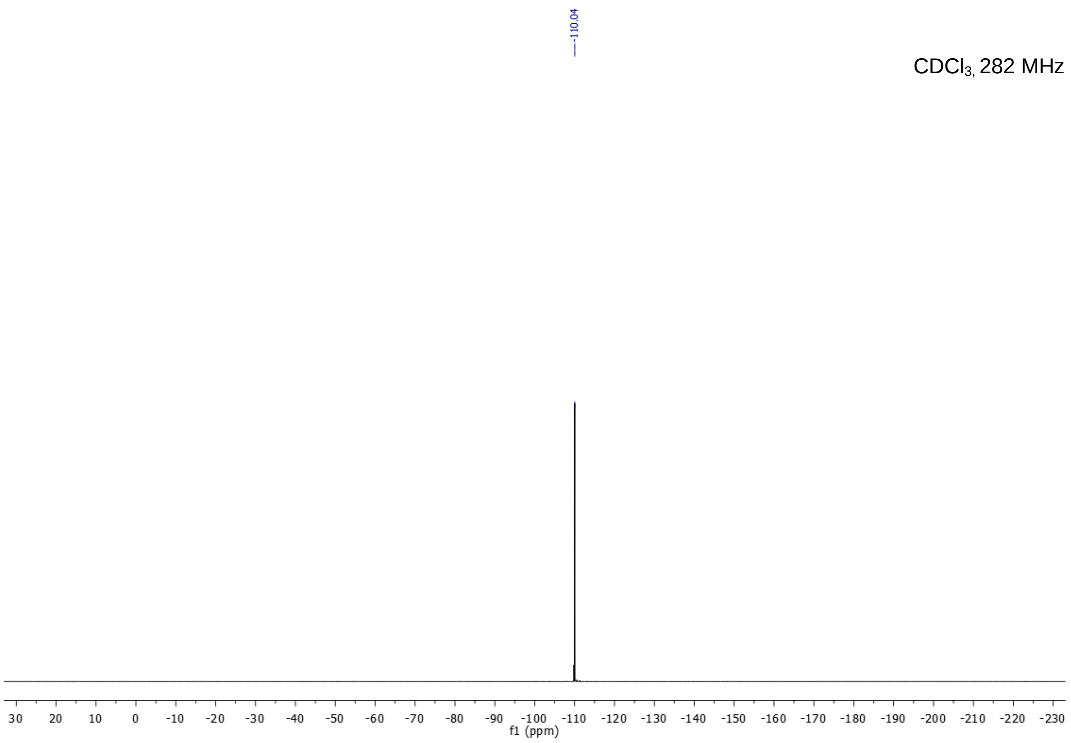

Figure S33. ^{1}H, ^{13}C and ^{19}F NMR spectra ($CDCl_3$) of compound **2g**.

(*Z*)-2-Chloro-1-phenylbut-2-en-1-ol (10a)

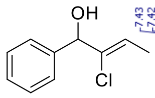

10a

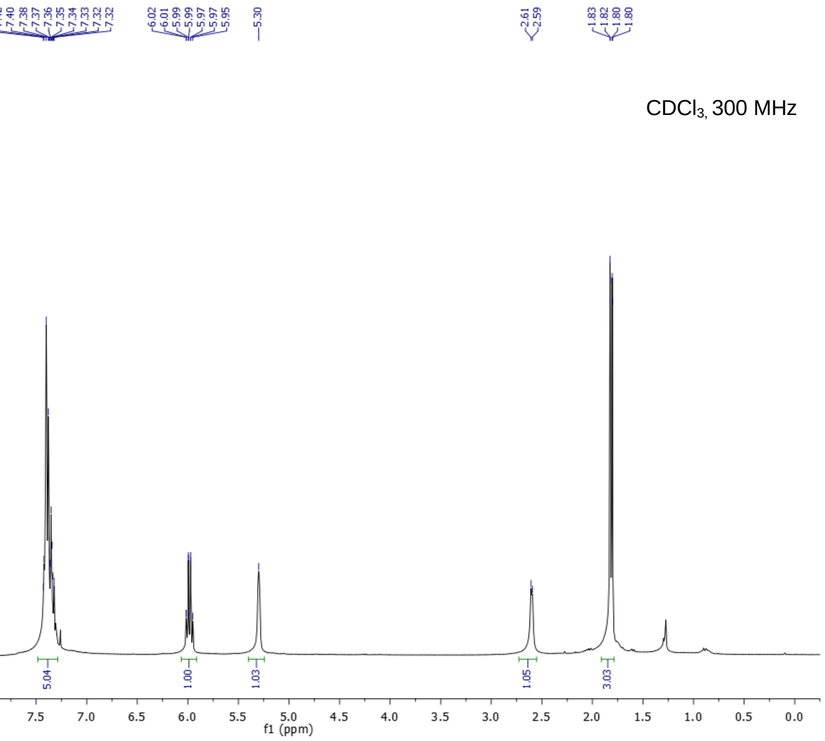

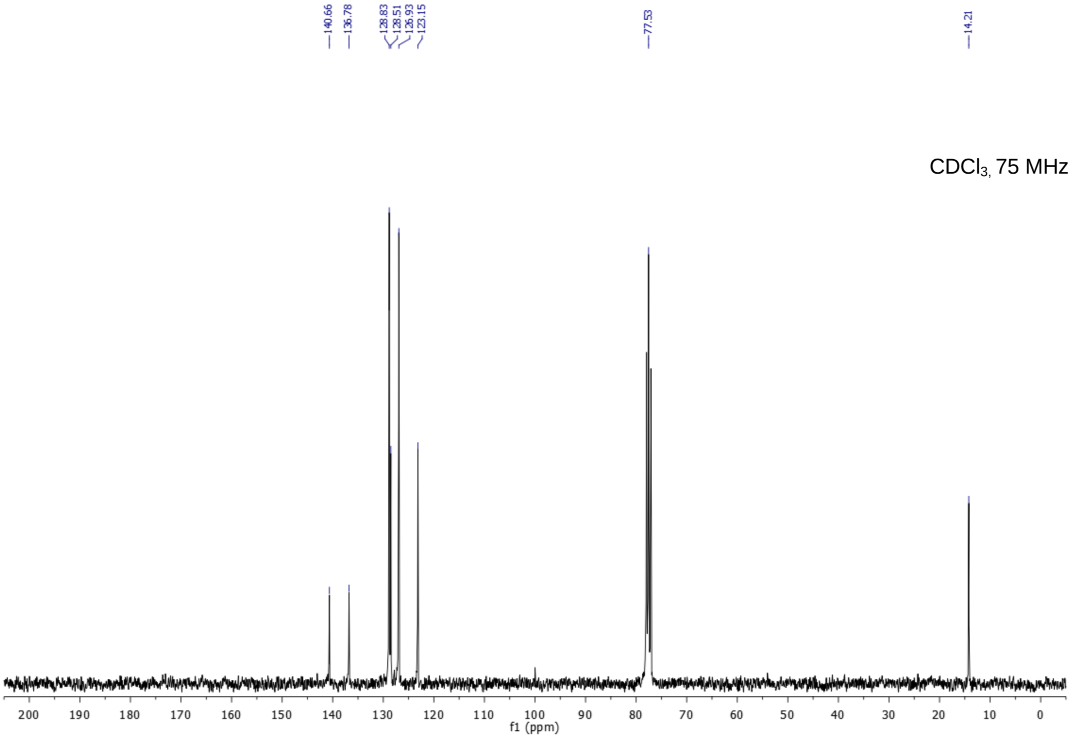

Figure S34. ^{1}H and ^{13}C NMR spectra ($CDCl_3$) of compound **10a**.

(1*R*,2*R*) and (1*S*,2*S*)-2-Chloro-1-phenylbutan-1-ol ((1*R*,2*R*)/(1*S*,2*S*)-3a)

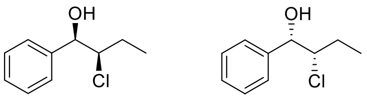

(1*R*,2*R*)-3a (1S,2S)-3a

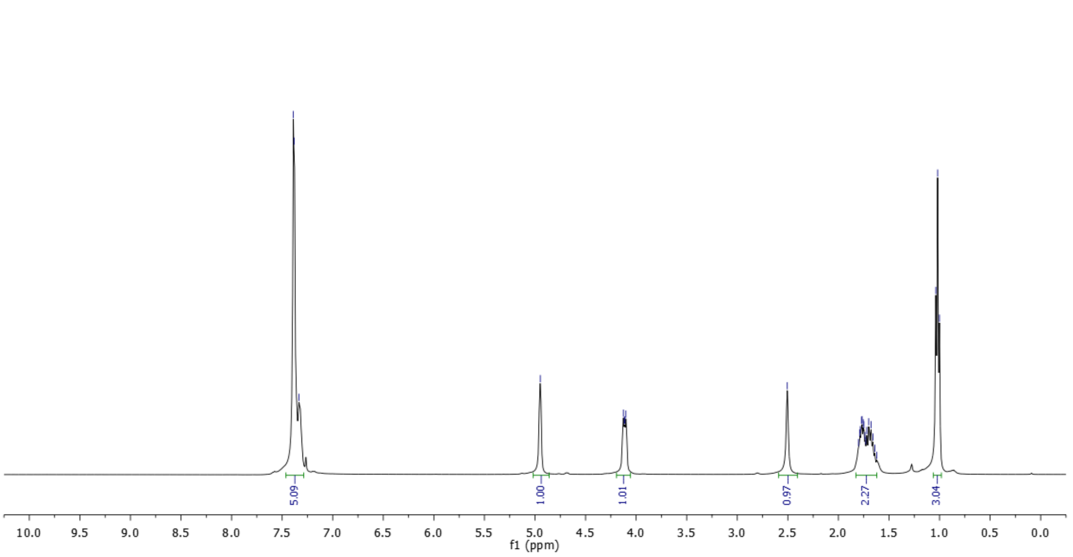

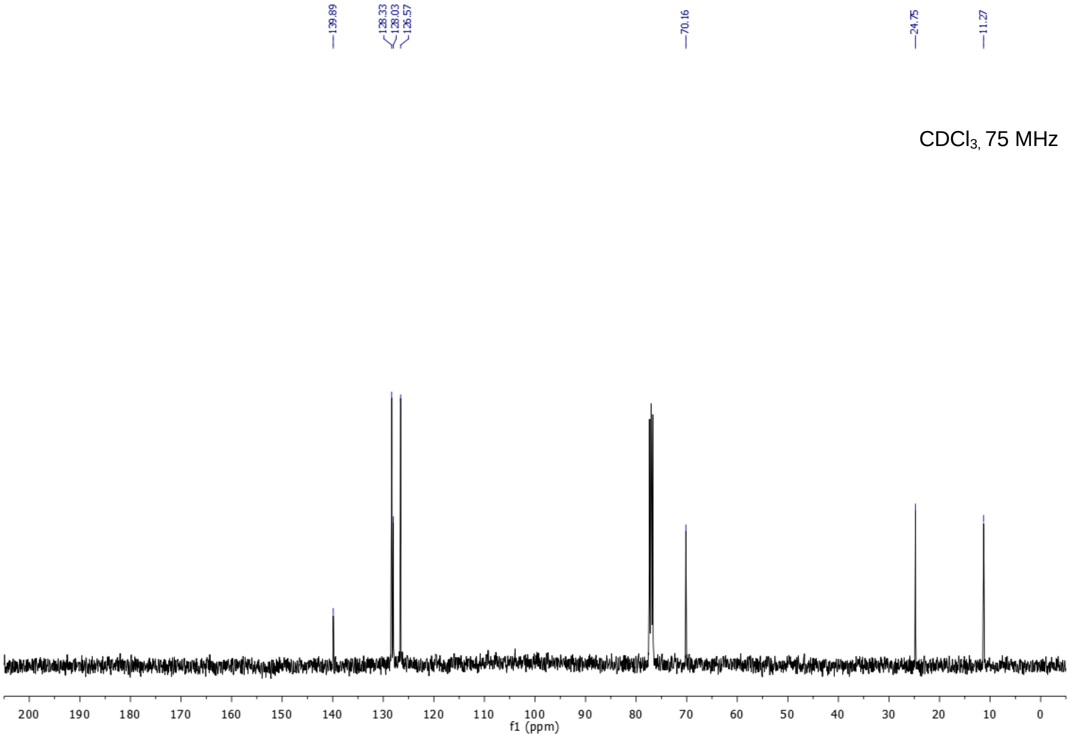

Figure S35. ^{1}H and ^{13}C NMR spectra ($CDCl_3$) of compounds (1*R*,2*R*)/(1*S*,2*S*)-**3a**

(1*S*,2*R*)-2-Chloro-1-phenylbutan-1-ol ((1*S*,2*R*)–3a)

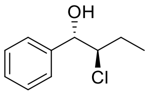

(1*S*,2*R*)-3a

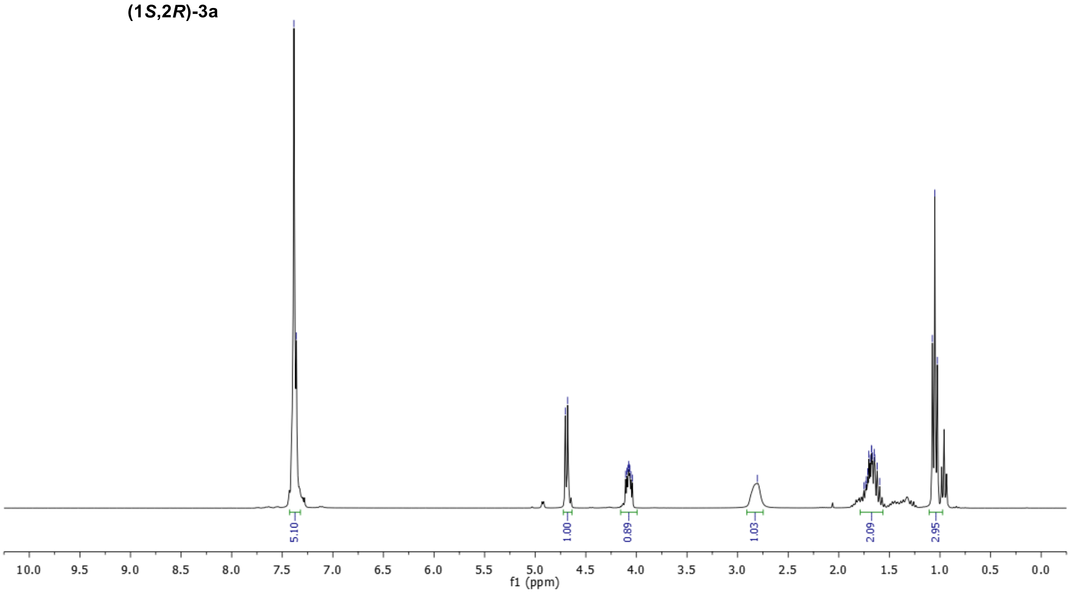

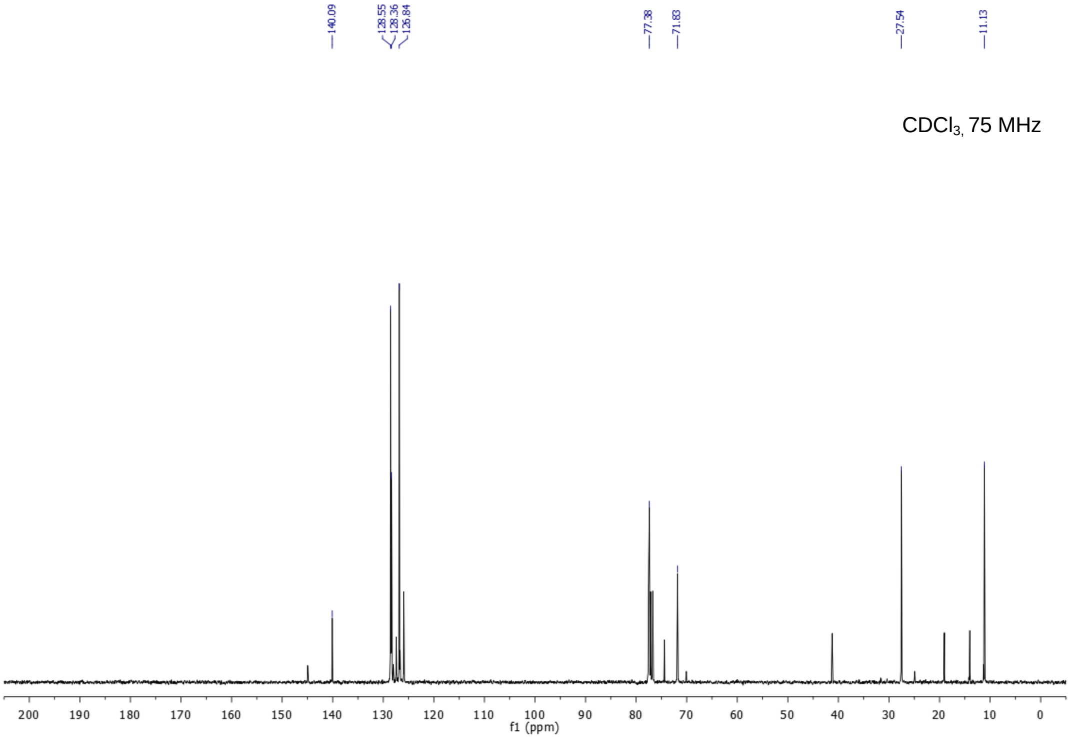

Figure S36. ^{1}H and ^{13}C NMR spectra ($CDCl_3$) of compound (1*S*,2*R*)-**3a**.

(1*R*,2*R*)-2-Chloro-1-(4-methylphenyl)butan-1-ol ((1*R*,2*R*)-3b)

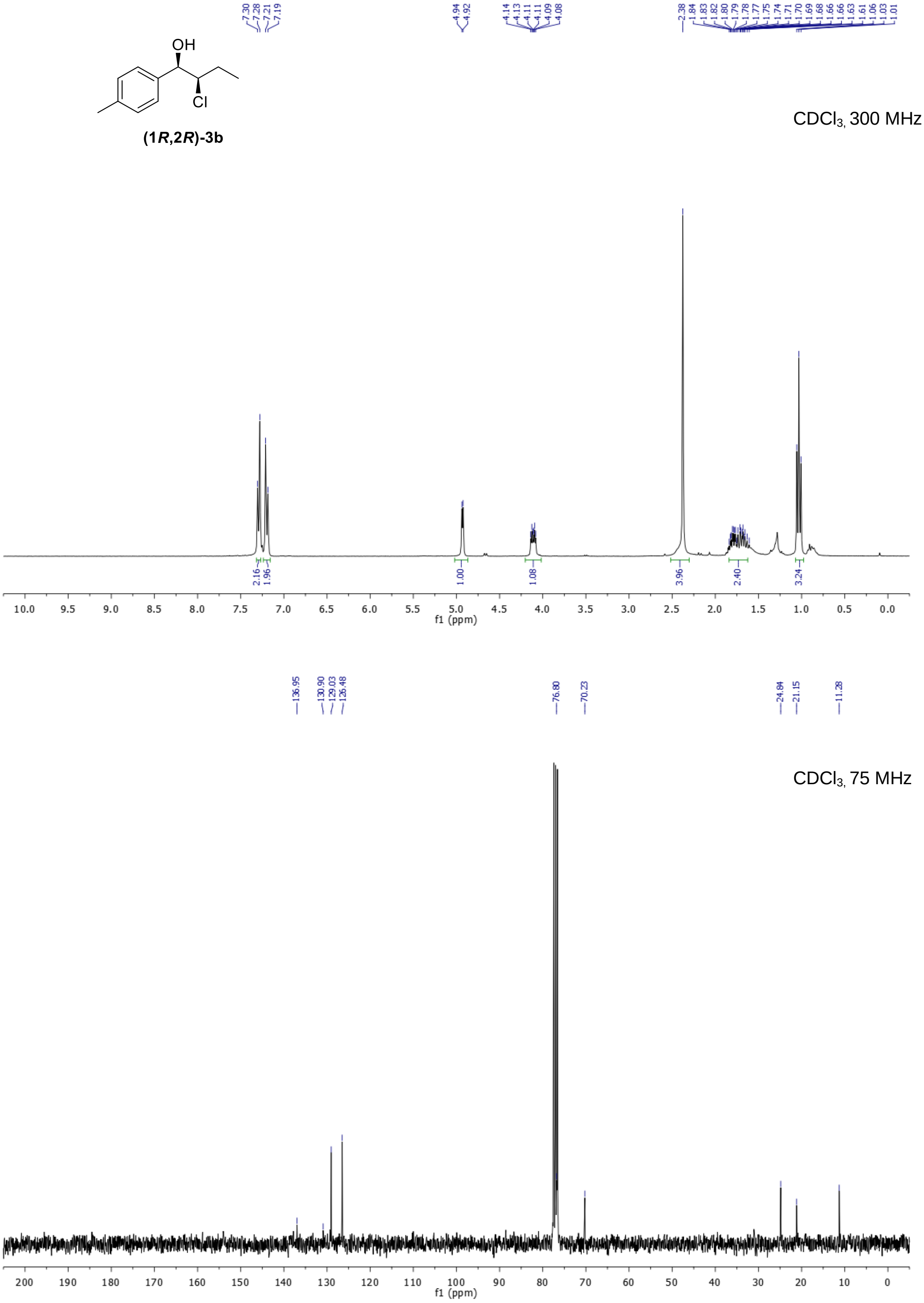

Figure S37. ^{1}H, and ^{13}C NMR spectra ($CDCl_3$) of compound (1*R*,2*R*)-**3b**.

(1*R*,2*S*)-2-Chloro-1-(4-methylphenyl)butan-1-ol ((1*R*,2*S*)-3b)

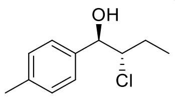

(1*R*,2*S*)-3b

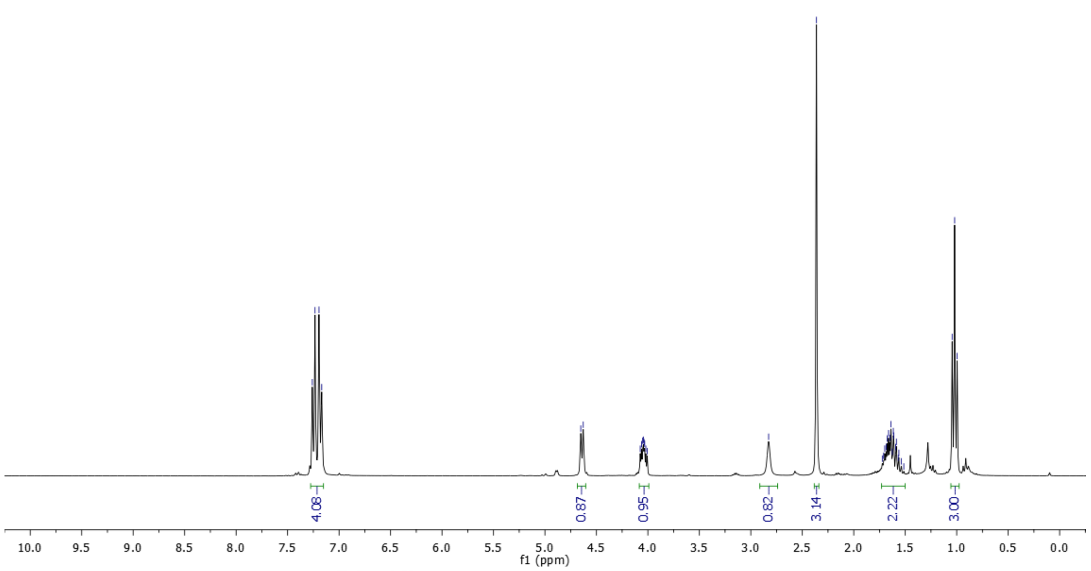

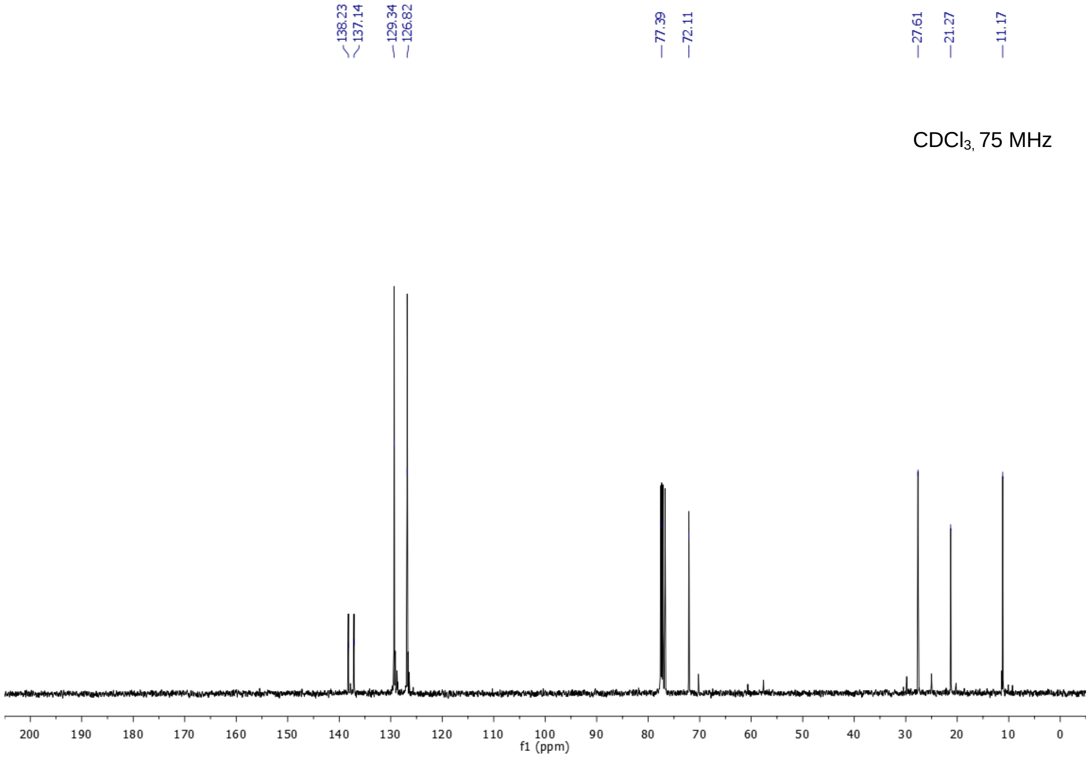

Figure S38. ^{1}H and ^{13}C NMR spectra ($CDCl_3$) of compound (1*R*,2*S*)-**3b**.

(1*R*,2*R*)-2-Chloro-1-(4-chlorophenyl)butan-1-ol ((1*R*,2*R*)-3c)

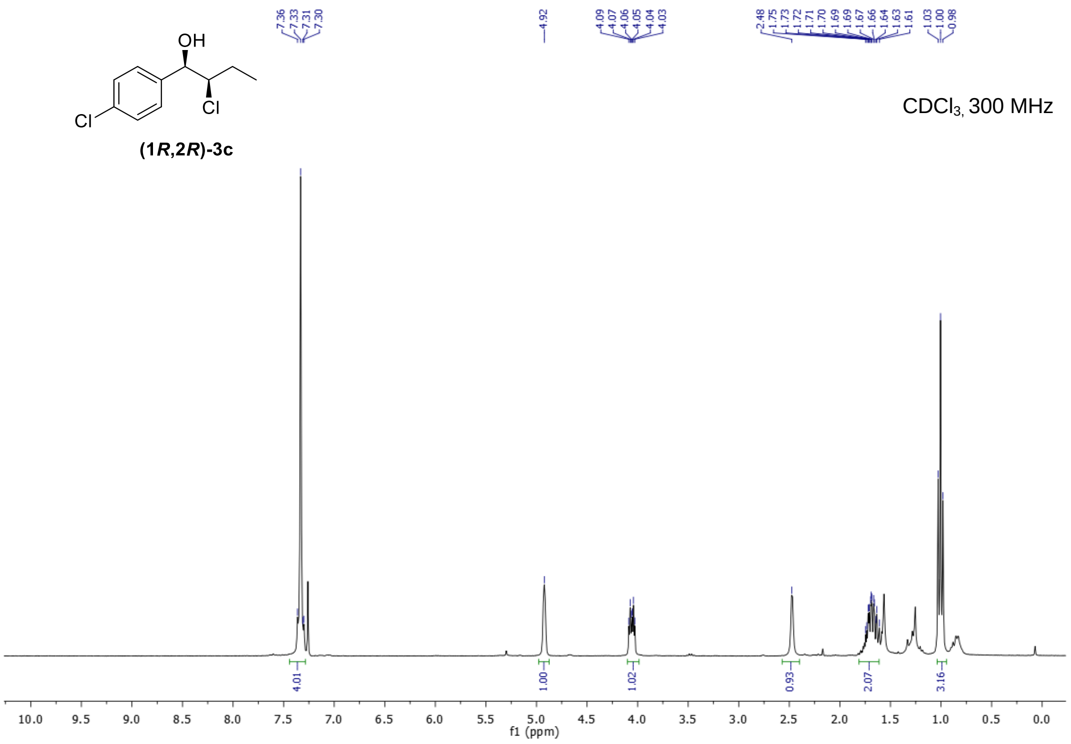

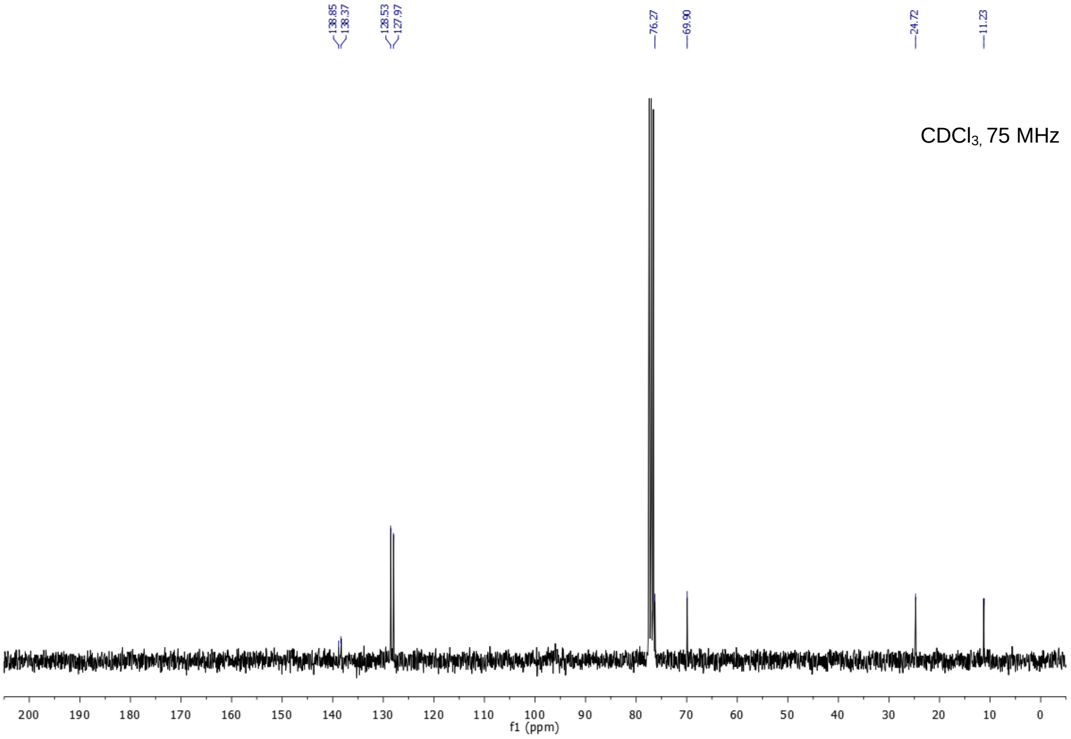

Figure S39. ^{1}H and ^{13}C NMR spectra ($CDCl_3$) of compound (1*R*,2*R*)-**3c**.

(1*S*,2*R*)-2-Chloro-1-(4-chlorophenyl)butan-1-ol ((1*S*,2*R*)-3c)

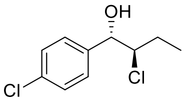

(1*S*,2*R*)-3c

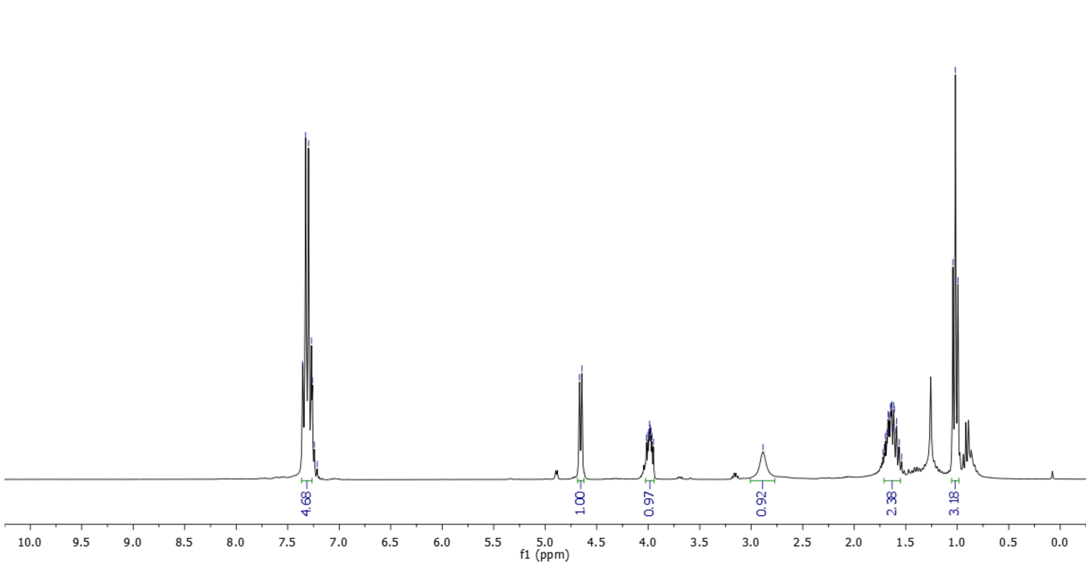

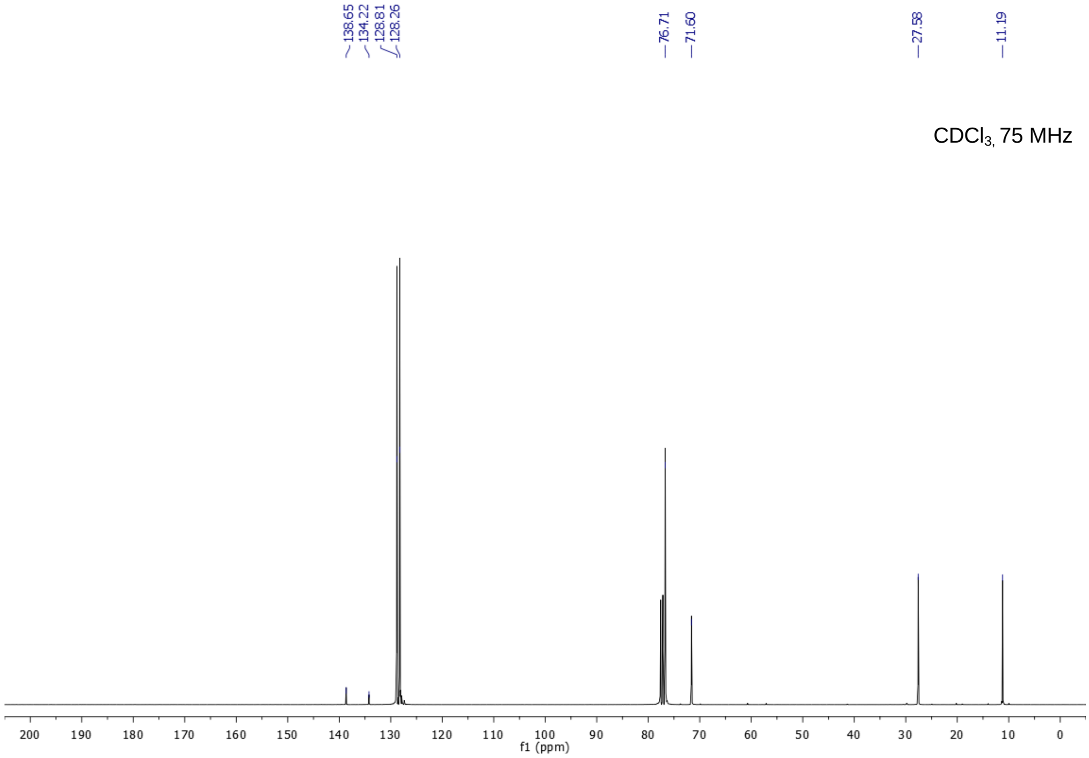

Figure S40. ^{1}H and ^{13}C NMR spectra ($CDCl_3$) of compound (1*S*,2*R*)-**3c**.

(1*R*,2*R*)-1-(4-Bromophenyl)-2-chlorobutan-1-ol ((1*R*,2*R*)-3d)

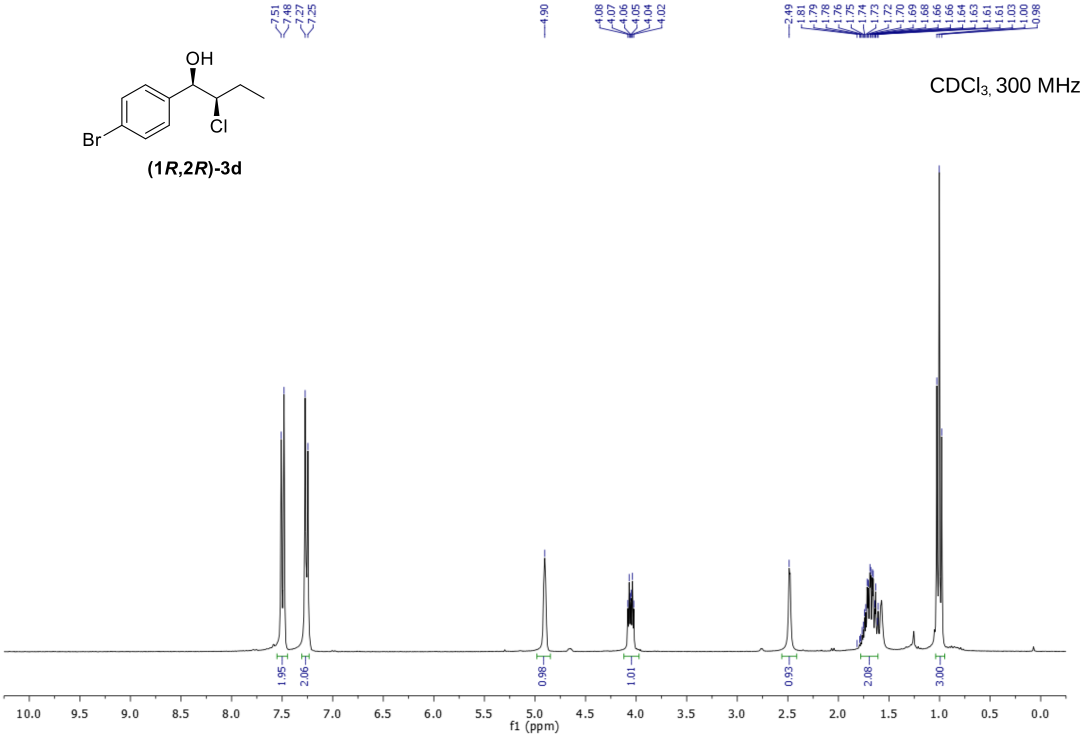

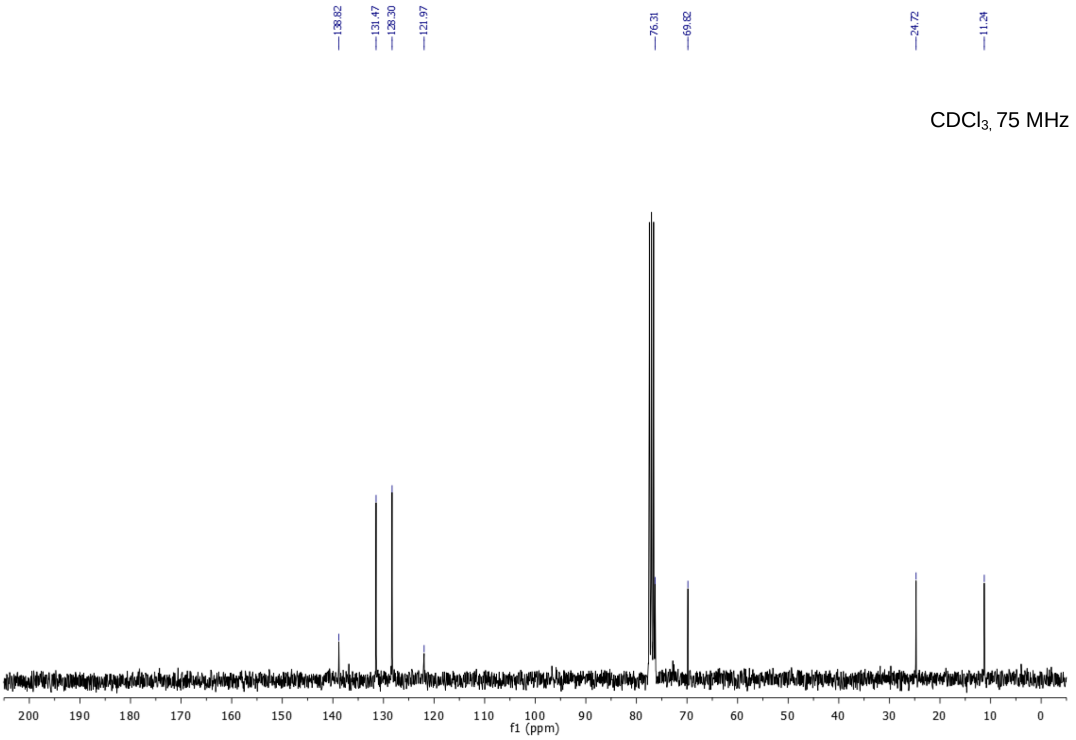

Figure S41. ^{1}H and ^{13}C NMR spectra ($CDCl_3$) of compound (1*R*,2*R*)-**3d**.

(1*S*,2*R*)-1-(4-Bromophenyl)-2-chlorobutan-1-ol ((1*S*,2*R*)-3d)

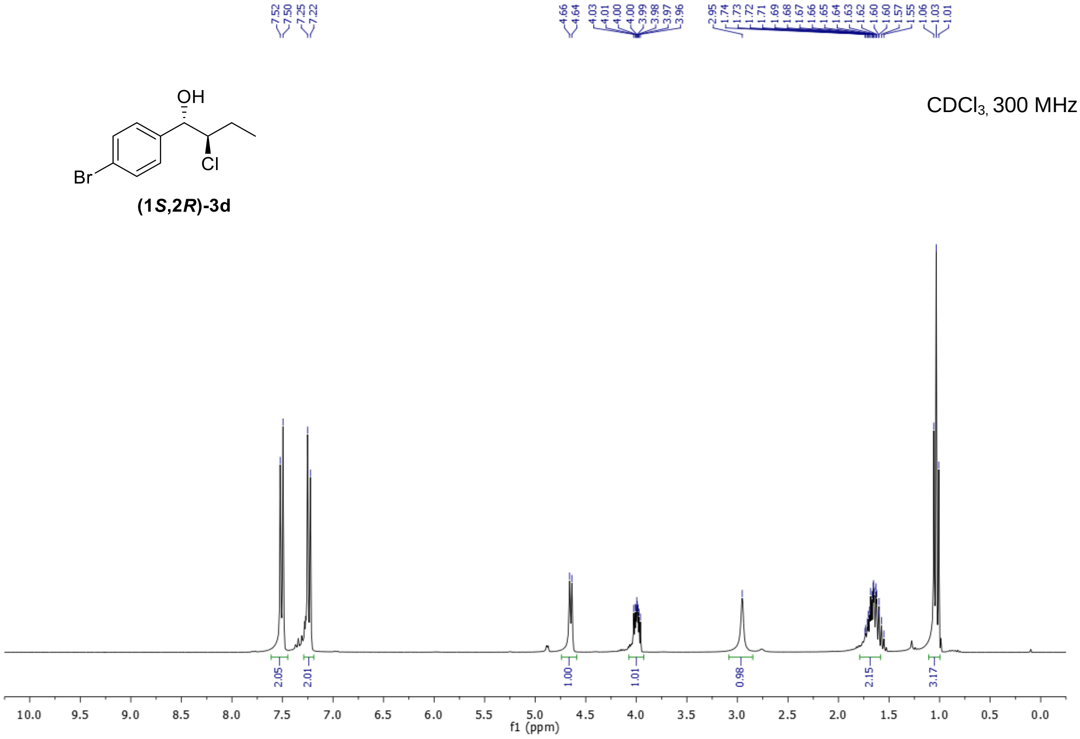

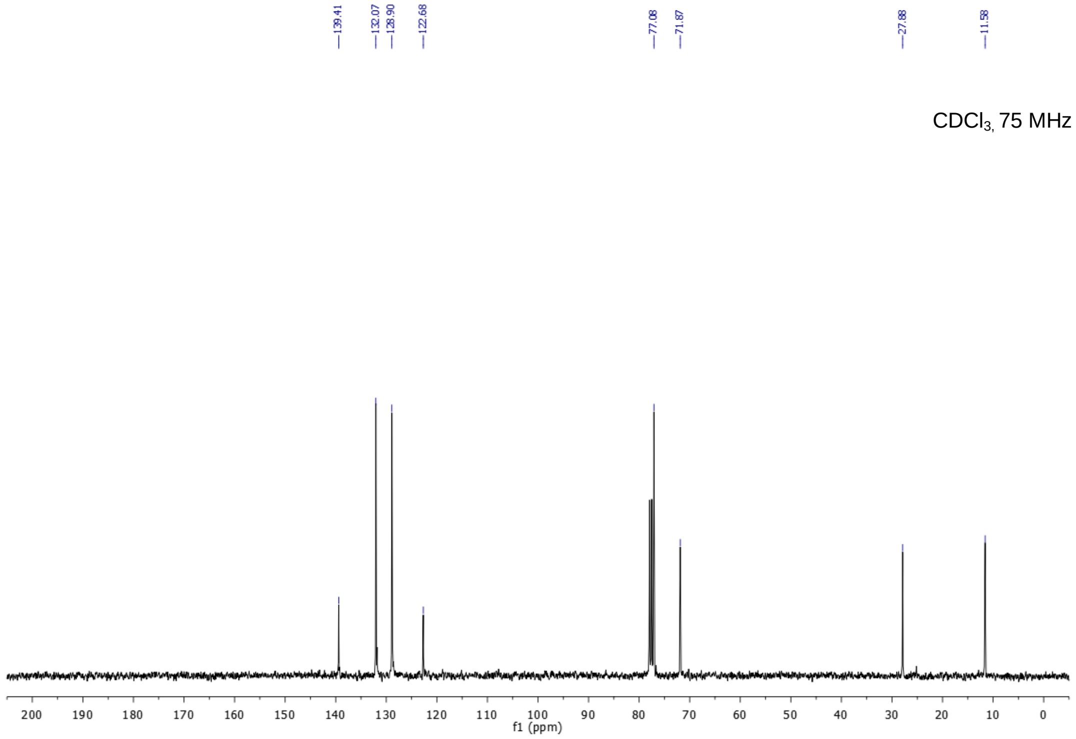

Figure S42. ^{1}H and ^{13}C NMR spectra ($CDCl_3$) of compound (1*S*,2*R*)-**3d**.

(1*S*,2*S*)-2-Chloro-1-(4-fluorophenyl)butan-1-ol ((1*S*,2*S*)-3e)

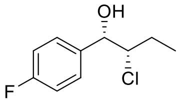

(1*S*,2*S*)-3e

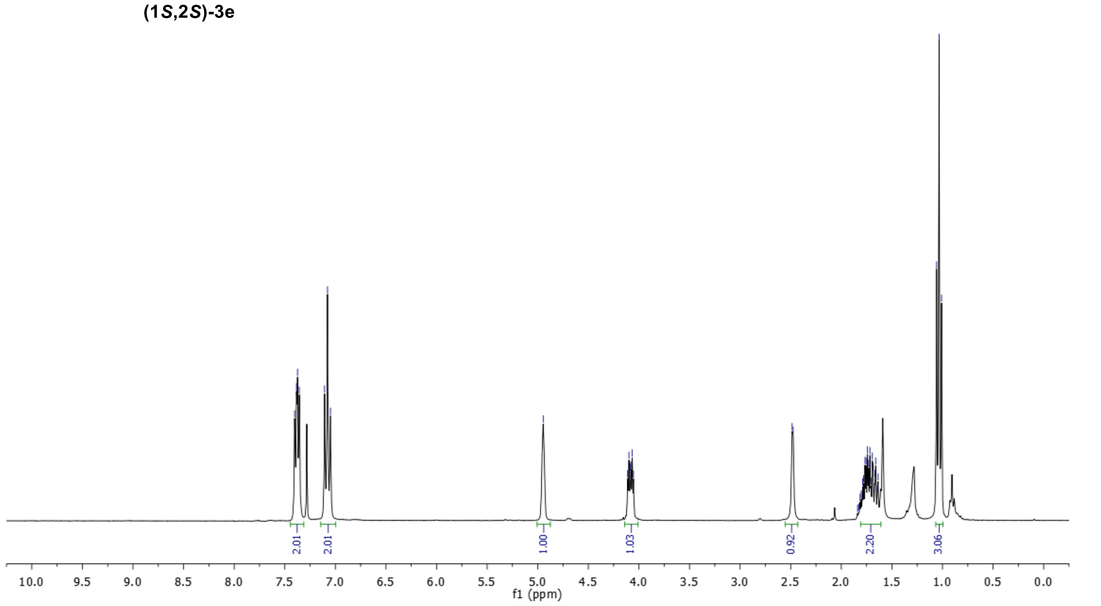

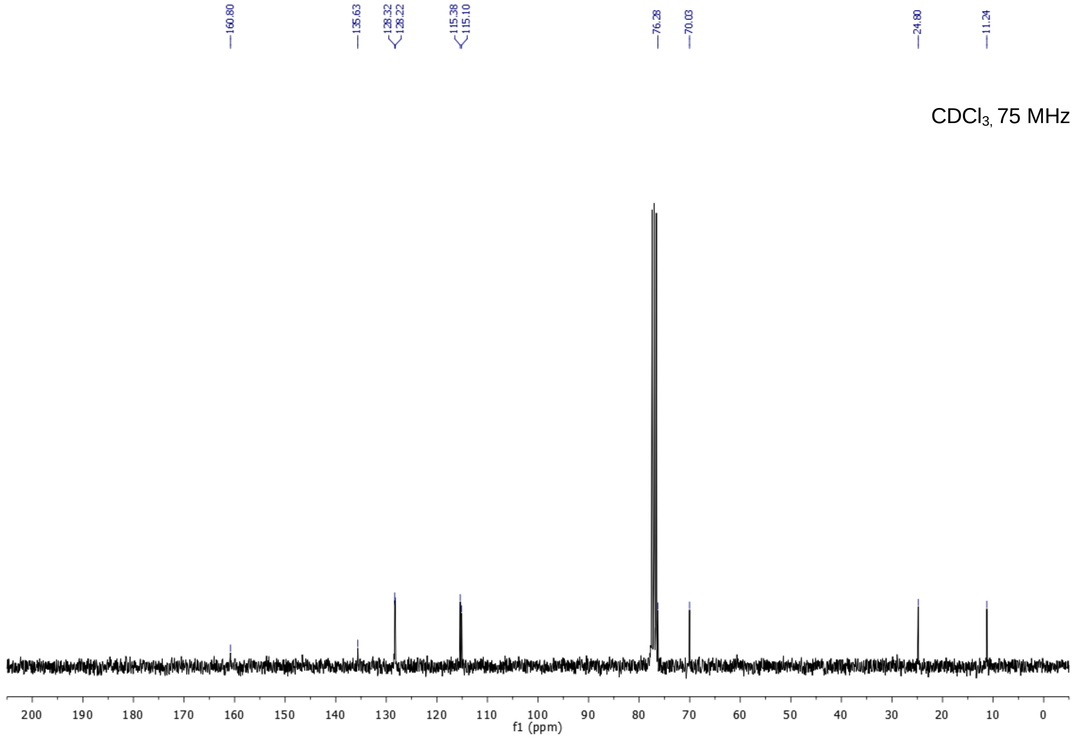

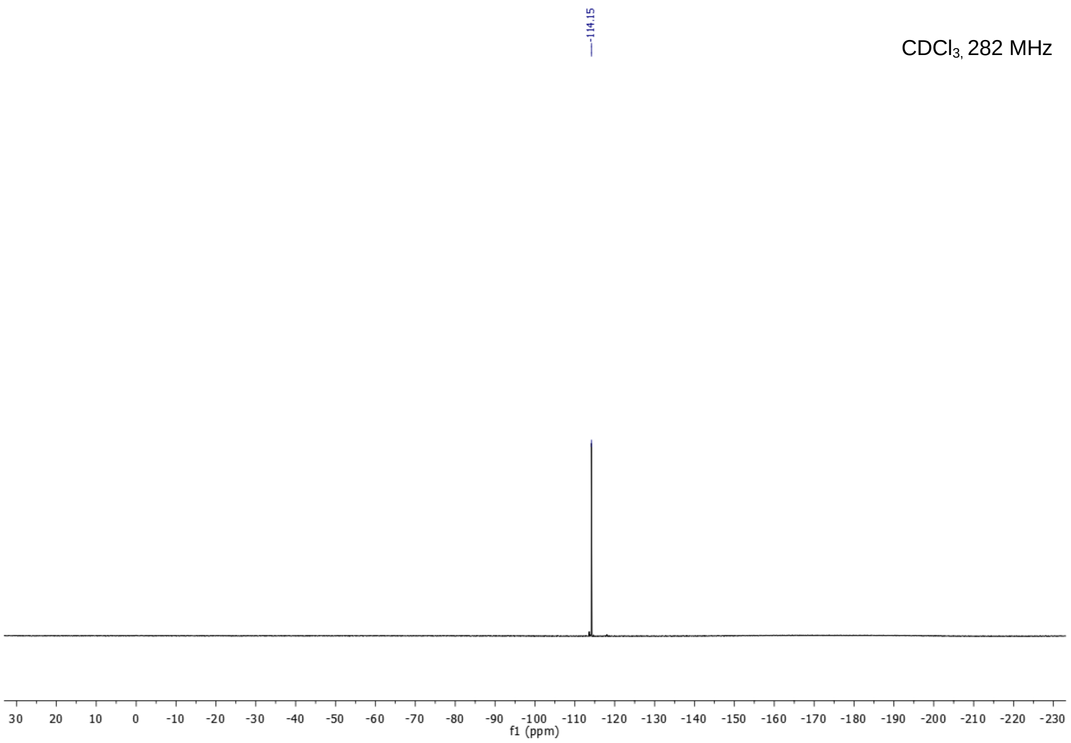

Figure S43. ^{1}H, ^{13}C and ^{19}F NMR spectra ($CDCl_3$) of compound (1*S*,2*S*)-**3e**.

(1*R*,2*S*)-2-Chloro-1-(4-fluorophenyl)butan-1-ol ((1*R*,2*S*)-3e)

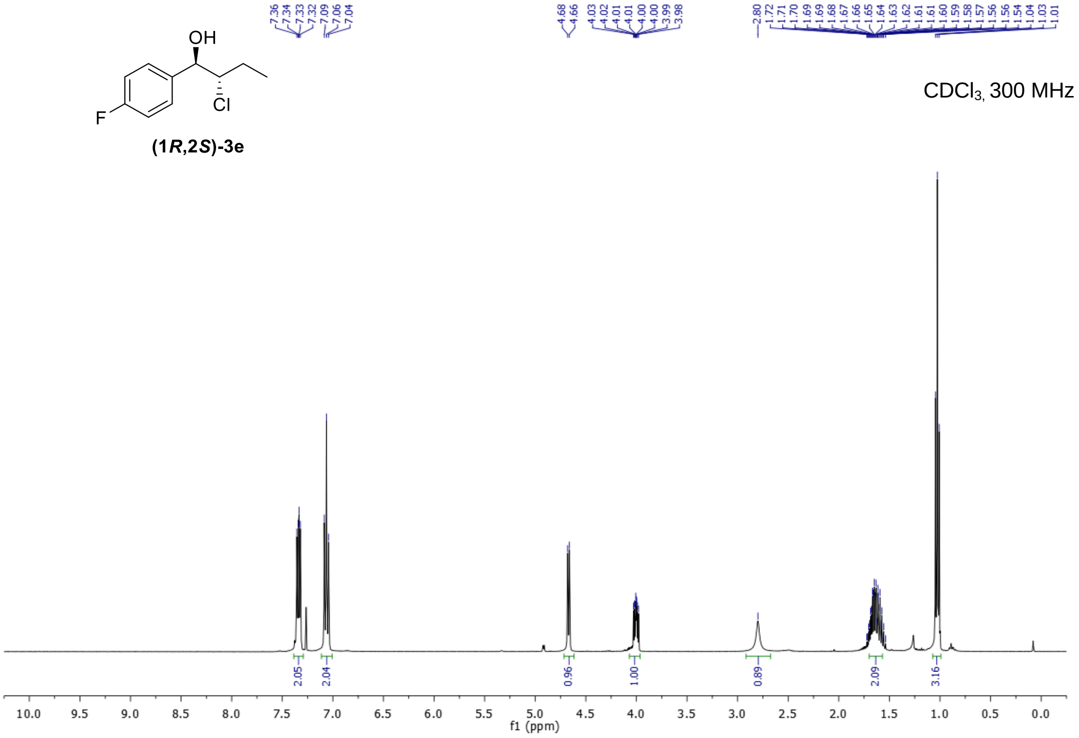

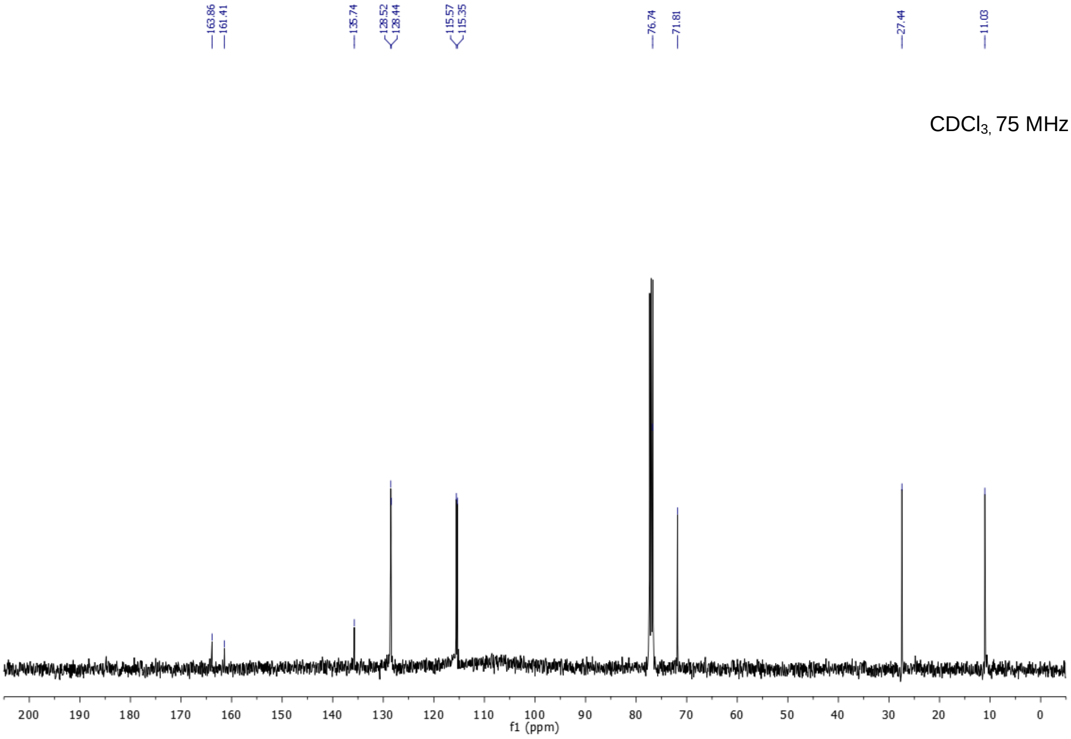

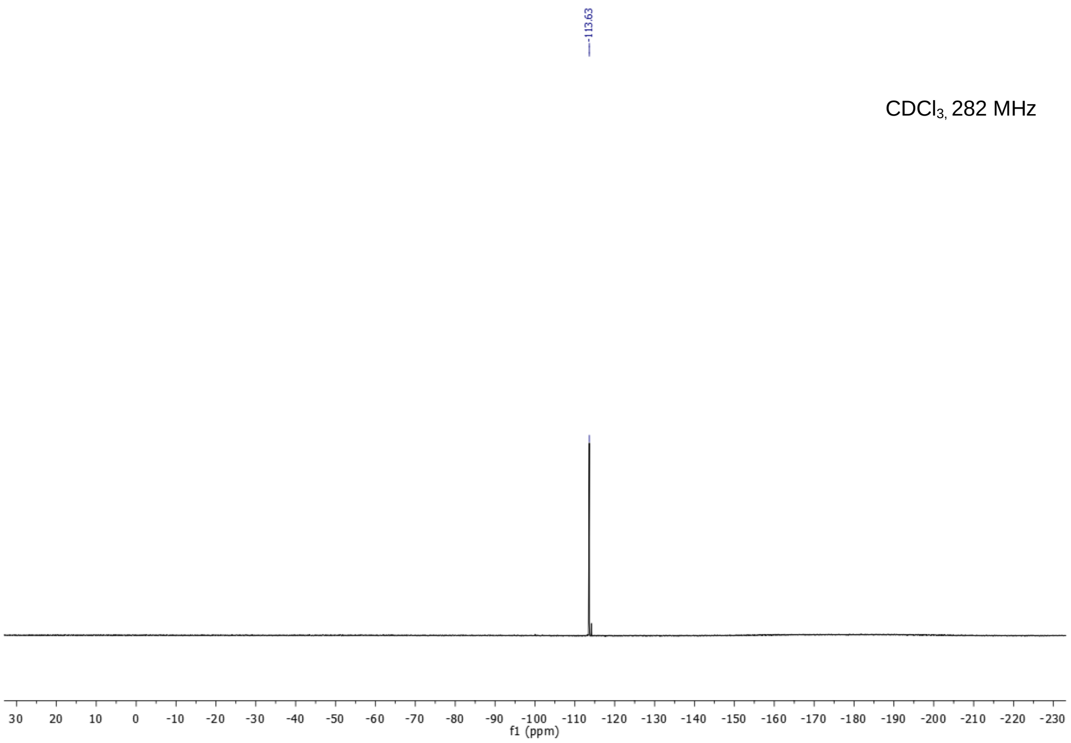

Figure S44. ^{1}H, ^{13}C and ^{19}F NMR spectra ($CDCl_3$) of compound (1*R*,2*S*)-**3e**.

(1*R*,2*R*)-2-Chloro-1-(3-fluorophenyl)butan-1-ol ((1*R*,2*R*)-3f)

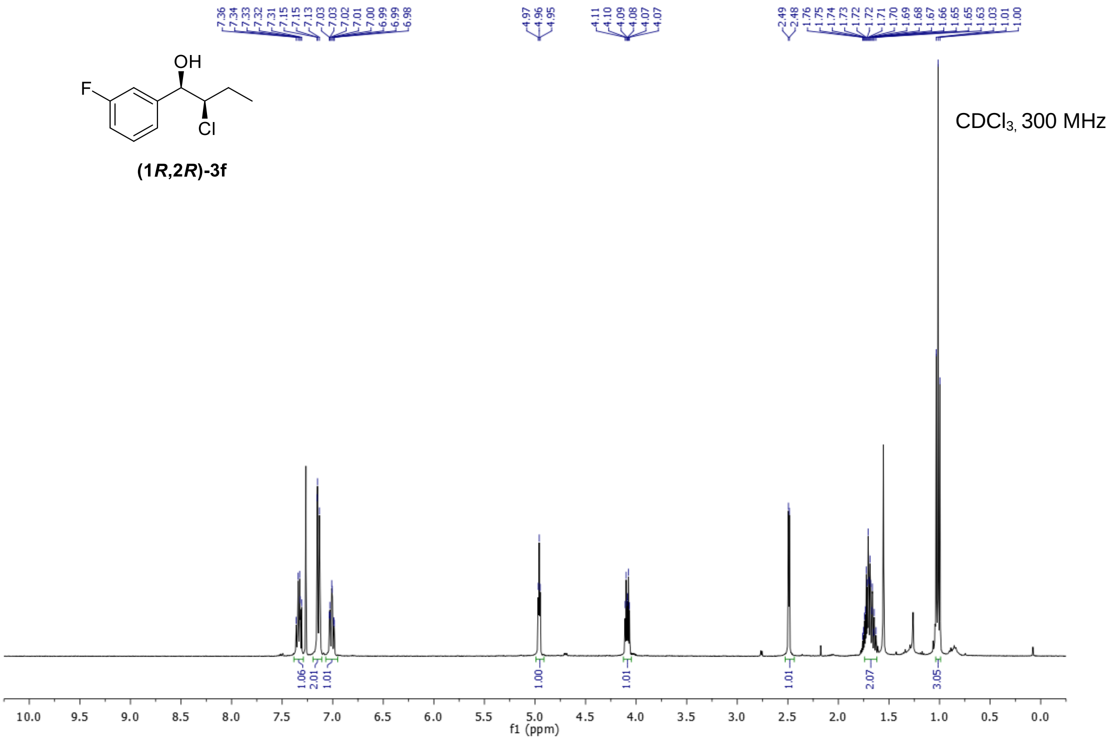

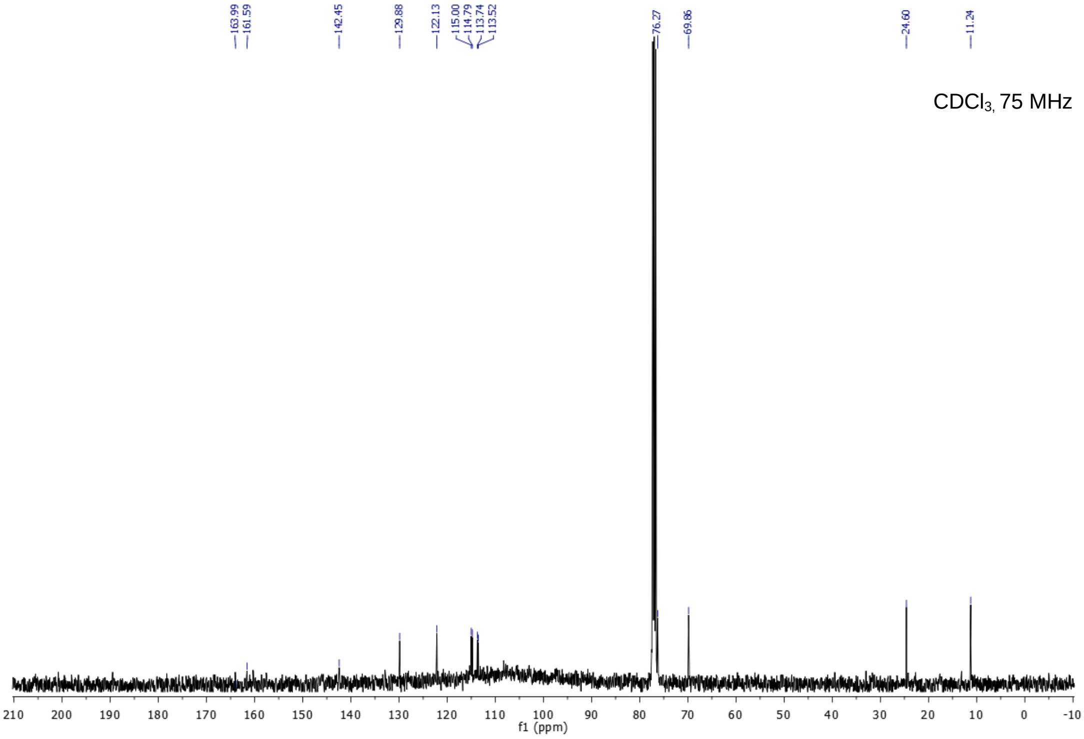

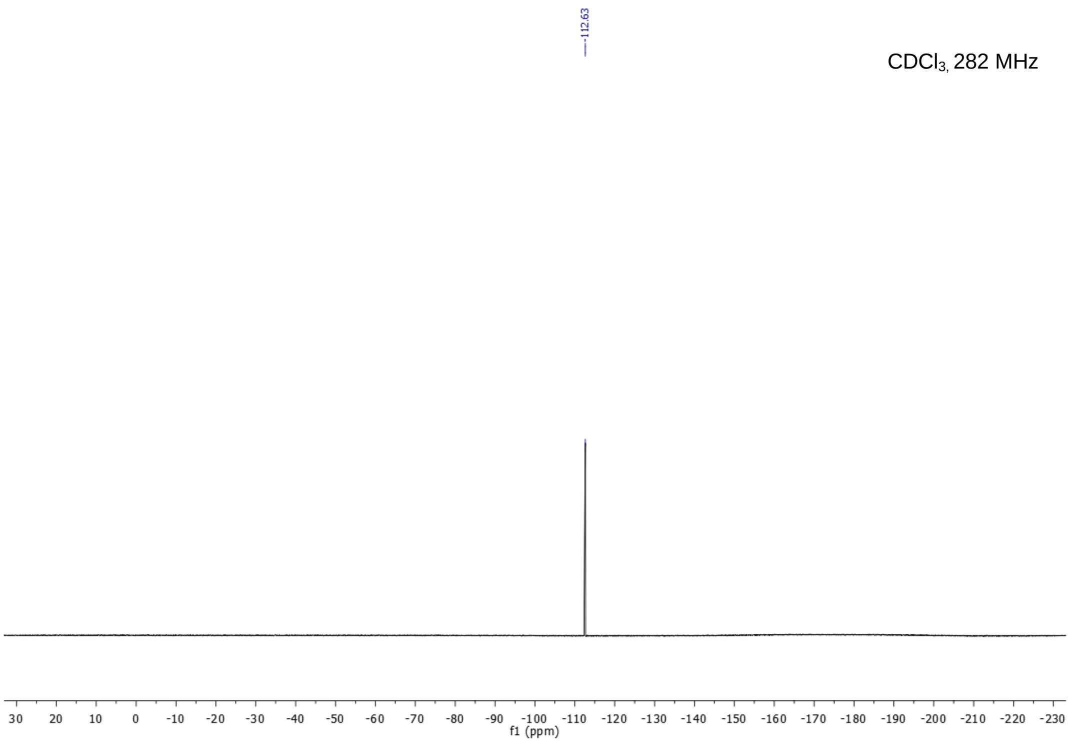

Figure S45. ^{1}H, ^{13}C and ^{19}F NMR spectra ($CDCl_3$) of compound (1*R*,2*R*)-**3f**.

(1*S*,2*R*)-2-Chloro-1-(3-fluorophenyl)butan-1-ol ((1*S*,2*R*)-3f)

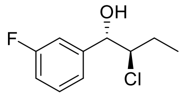

(1*S*,2*R*)-3f

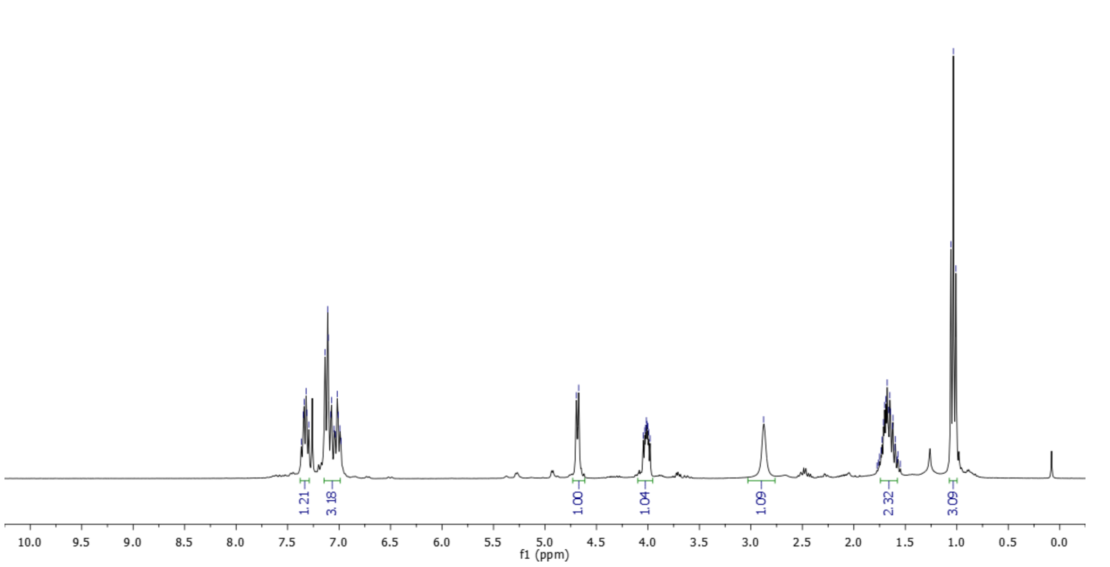

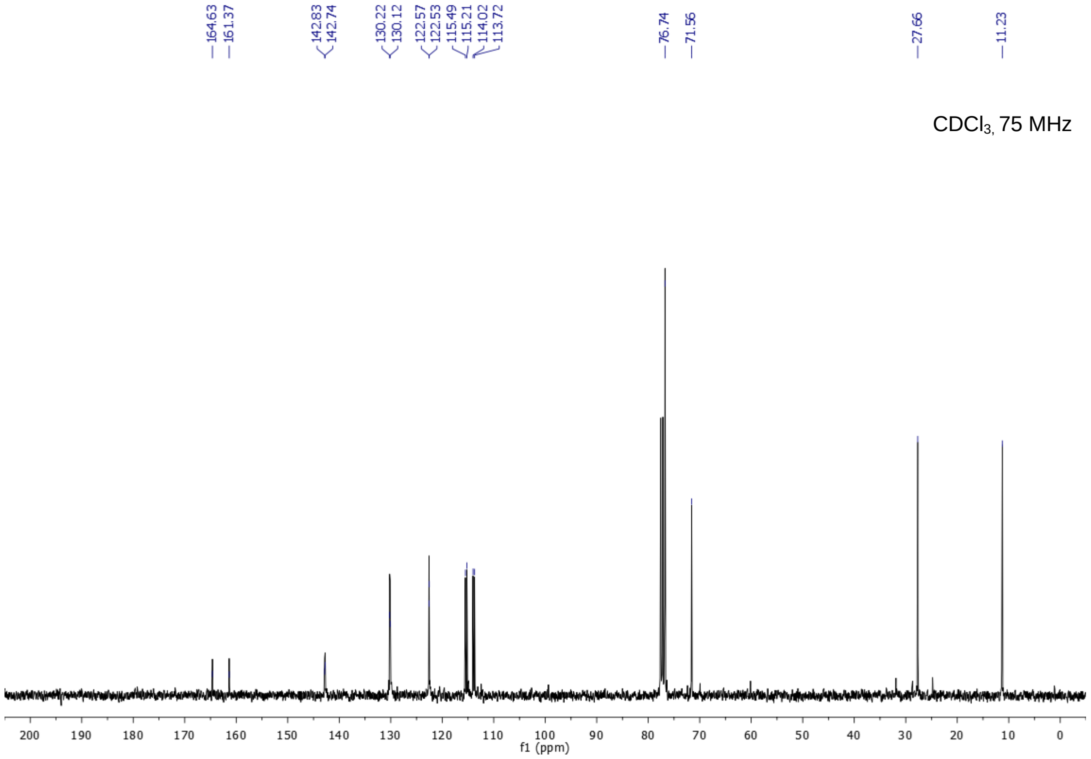

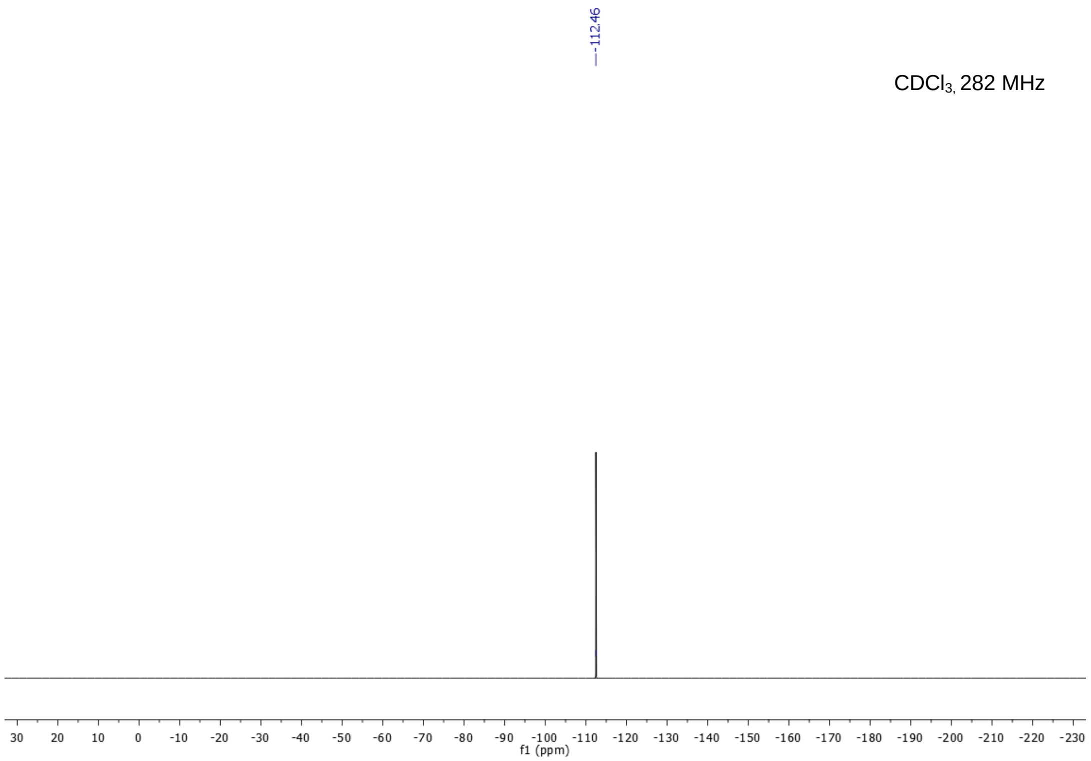

Figure S46. ^{1}H, ^{13}C and ^{19}F NMR spectra ($CDCl_3$) of compound (1*S*,2*R*)-**3f**.

(1*R*,2*R*)-2-Chloro-1-(2-fluorophenyl)butan-1-ol ((1*R*,2*R*)-3g)

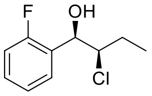

(1*R*,2*R*)-3g

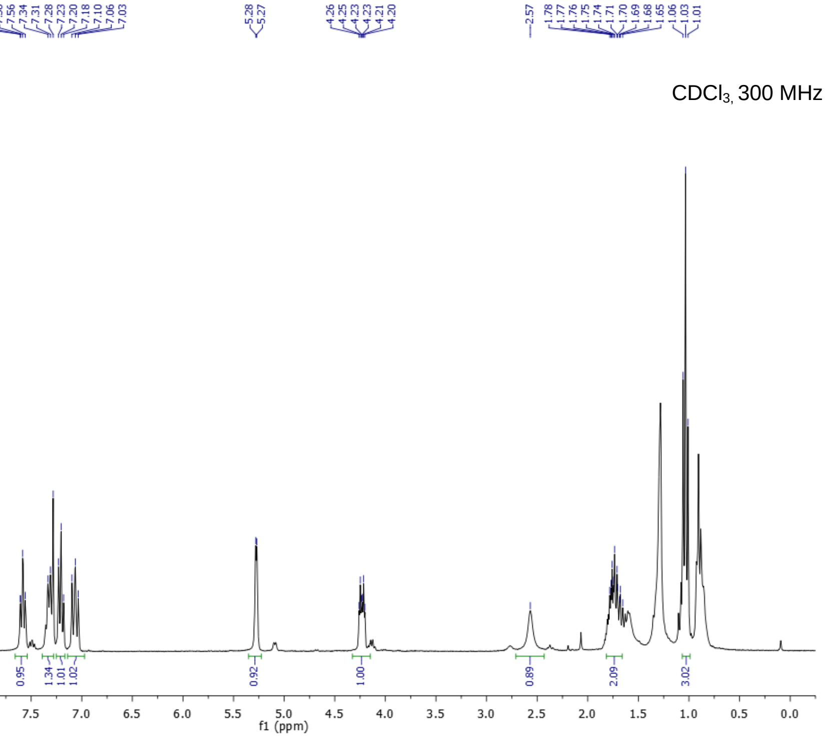

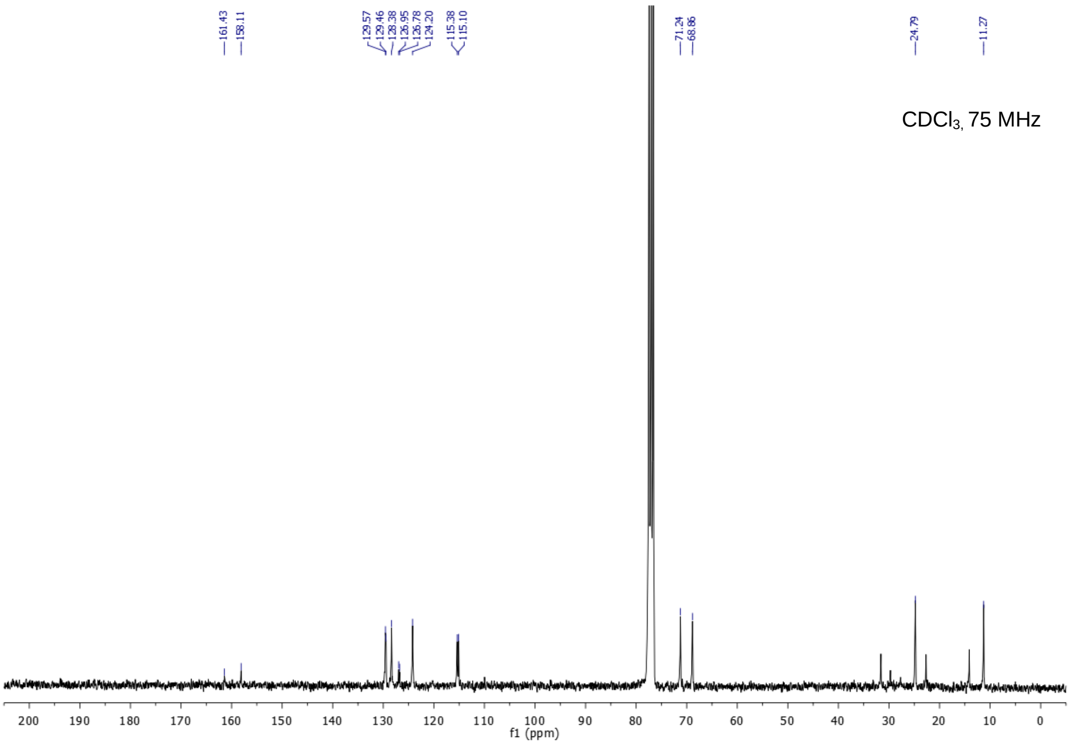

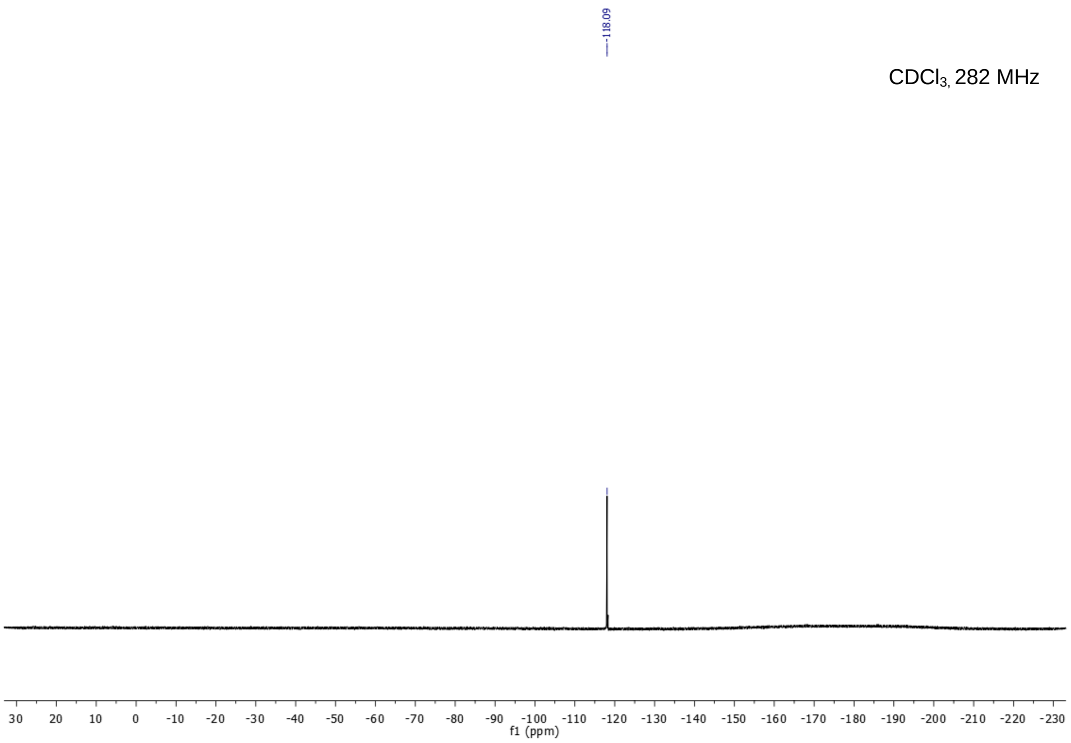

Figure S47. ^{1}H, ^{13}C and ^{19}F NMR spectra ($CDCl_3$) of compound (1*R*,2*R*)-**3g**.

(1*S*,2*R*)-2-Chloro-1-(2-fluorophenyl)butan-1-ol ((1*S*,2*R*)-3g)

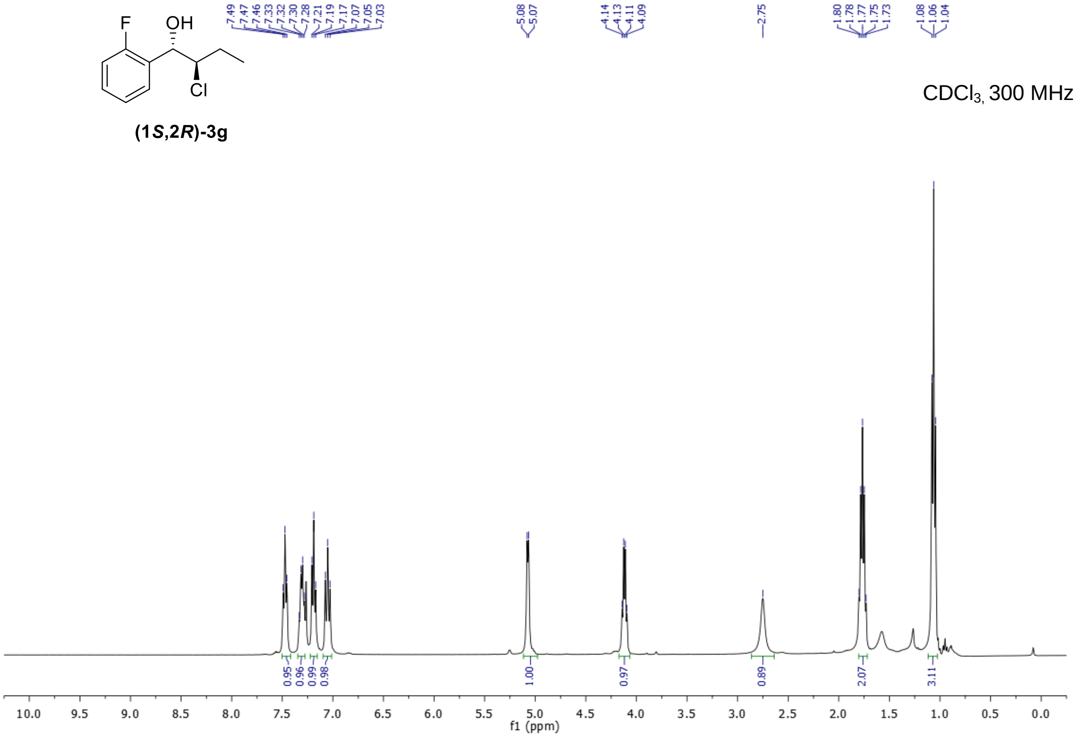

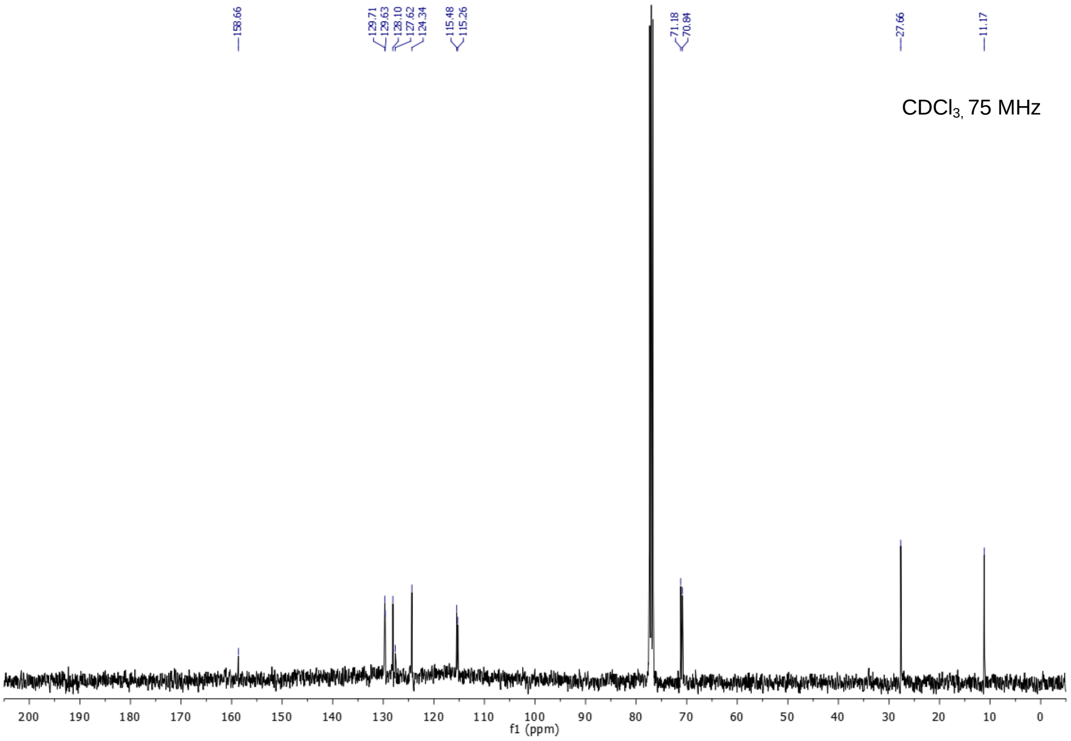

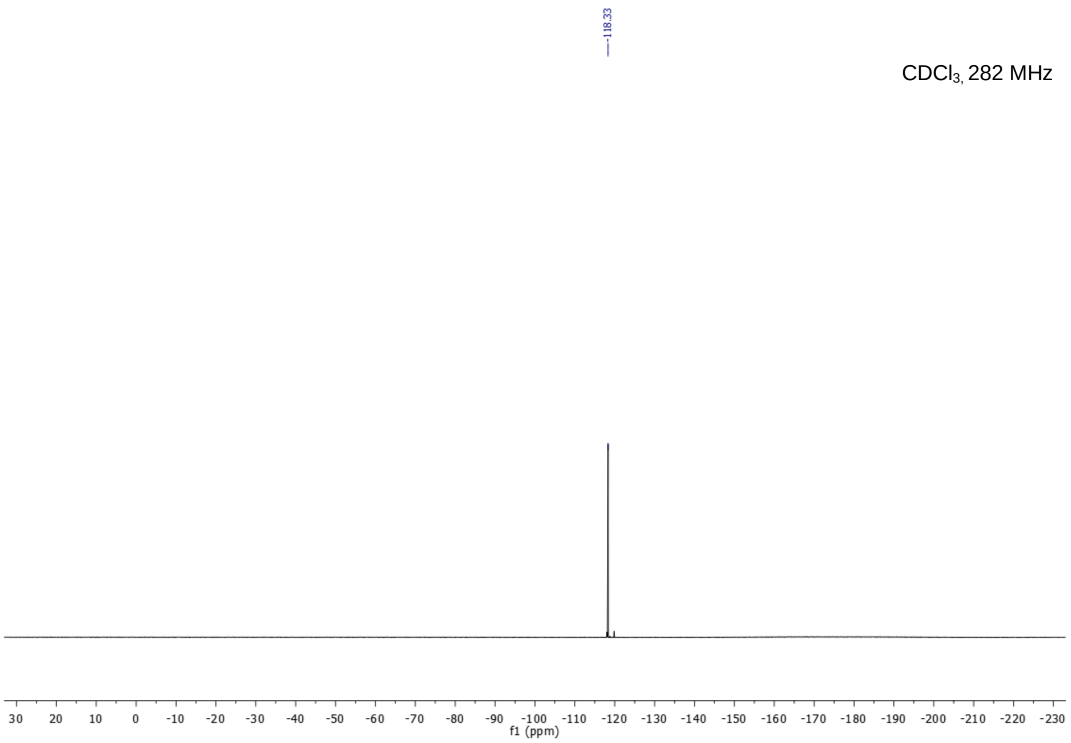

Figure S48. ^{1}H, ^{13}C and ^{19}F NMR spectra ($CDCl_3$) of compound (1*S*,2*R*)-**3g**.
